# Adaptation of *Lactococcus lactis* to high growth temperature leads to a dramatic increase in acidification rate

**DOI:** 10.1038/srep14199

**Published:** 2015-09-21

**Authors:** Jun Chen, Jing Shen, Lars Ingvar Hellgren, Peter Ruhdal Jensen, Christian Solem

**Affiliations:** 1 National Food Institute, Technical University of Denmark, DK-2800 Kgs. Lyngby, Denmark; 2 Department of Systems Biology, Technical University of Denmark, DK-2800 Kgs. Lyngby, Denmark

## Abstract

*Lactococcus lactis* is essential for most cheese making, and this mesophilic bacterium has its growth optimum around 30 °C. We have, through adaptive evolution, isolated a mutant TM29 that grows well up to 39 °C, and continuous growth at 40 °C is possible if pre-incubated at a slightly lower temperature. At the maximal permissive temperature for the wild-type, 38 °C, TM29 grows 33% faster and has a 12% higher specific lactate production rate than its parent MG1363, which results in fast lactate accumulation. Genome sequencing was used to reveal the mutations accumulated, most of which were shown to affect thermal tolerance. Of the mutations with more pronounced effects, two affected expression of single proteins (chaperone; riboflavin transporter), two had pleiotropic effects (RNA polymerase) which changed the gene expression profile, and one resulted in a change in the coding sequence of CDP-diglyceride synthase. A large deletion containing 10 genes was also found to affect thermal tolerance significantly. With this study we demonstrate a simple approach to obtain non-GMO derivatives of the important *L. lactis* that possess properties desirable by the industry, e.g. thermal robustness and increased rate of acidification. The mutations we have identified provide a genetic basis for further investigation of thermal tolerance.

*Lactococcus lactis* (*L*. *lactis*) is a Gram-positive bacterium, belonging to the group of lactic acid bacteria (LAB). The main fermentation product usually is lactic acid, and glycolysis is the main source for energy^1^. *L*. *lactis* is mostly known for its enormous importance in the dairy industry, where more than 100 million tons of milk annually is inoculated with this bacterium for production of various fermented dairy products^2^. Moreover, the many years of safe use has made *L*. *lactis* a favoured candidate for other important applications as well, e.g. as a delivery vehicle for vaccines and therapeutic peptides for human use^3^ and as a production platform for various compounds^4^,^5^,^6^.

*L*. *lactis* experiences many environmental stresses during industrial handling, and it is highly important that that strains used are sufficiently robust to ensure optimal performance under these conditions. Typical stresses are imposed by factors such as heat, oxygen, low pH and high salinity. In cheese production, for instance, during the curdling process, the temperature is often raised to around 40 °C, or even beyond, and here *L*. *lactis* stops growing and lactate production is drastically reduced (personal communication Søren Lillevang, ARLA foods).

Heat stress is one of the most extensively studied topics for *L. lactis* and thermally robust strains are interesting for the industry, not only for robustness issues but also because a high fermentation temperature could have other advantages such as faster acidification, less by-product formation, and a reduced risk of phage infection^7^,^8^,^9^. Many studies have focused on the molecular basis of the heat stress response and especially a large effort has been made to understand the important roles of heat shock proteins (HSPs) and their regulatory mechanisms. The chaperone complexes DnaK-GrpE-DnaJ and GroES- GroEL have considerable importance, not only in protein folding, relocation and assembly^10^, but also for other biological processes such as cell membrane stabilization and DNA replication^10^,^11^ when cells are exposed to heat. Over-expression of HSPs has been demonstrated to improve the thermal tolerance of *L. lactis*. For instance, the over-expression of GroES-GroEL in *L. lactis* significantly improved the thermal tolerance and the tolerance to other stresses^12^.

When the heterologous DnaK from *E. coli* was over-expressed in *L. lactis*, the strain grew at a higher temperature, and a higher specific productivity of lactate was observed^13^. Substantial over-expression of genes, however, can lead to a decline in growth rate, which affects the volumetric productivity^14^, and in addition, for food applications such GMO strains are currently not acceptable in many parts of the world^15^.

Adaptive laboratory evolution (ALE), which allows for the selection of desirable phenotypes through the simulation of natural evolution in a laboratory environment, gradually has become a popular tool for obtaining microorganisms with particular properties. During ALE, genetic variations occur across the chromosome, and beneficial mutations, which are able to improve growth rate, are fixed. ALE also allows for genetic reorganisations towards optimal phenotypes to various conditions without prior knowledge. So far, ALE has been applied for isolating thermo-tolerant mutants of well-characterized platform organisms such as *E. coli* and *S. cerevisiae*^16^,^17^, but the distinctive genetic backgrounds and ecological niches where these organisms thrive, makes it difficult to compare the results obtained with those obtained for an LAB like *L. lactis*. In this study, we have used ALE to isolate a thermo-tolerant *L. lactis* mutant, TM29, which produces lactate at a faster rate at high temperatures. Our starting point was the well-characterized laboratory strain *L. lactis* ssp. *cremoris* MG1363^18^. After exposure to high temperatures in chemically defined medium using a serial-transfer regime, a mutant capable of growing at 40 °C was isolated. Physiological and trancriptomic characterization was conducted at different temperatures. We also identified the mutations accumulated by using Next-Generation Sequencing, which allowed us to reveal the genetic alterations involved in the thermal adaptation. Furthermore, the contribution of the individual mutations to thermal tolerance was evaluated by introducing them into the wild-type background.

## Results

### Physiological characterization of TM29 and MG1363 at different temperatures

**Figure 1:**
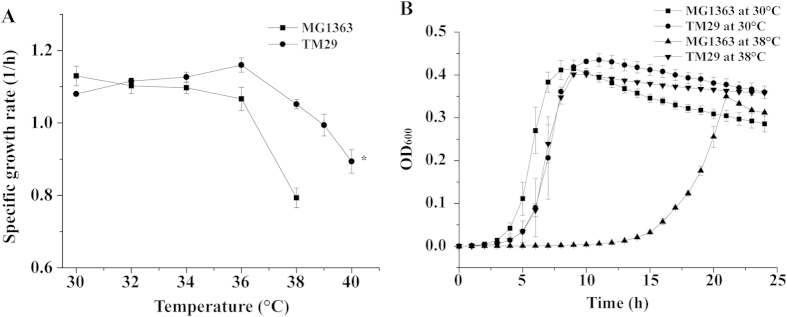
Growth characteristics of MG1363 and TM29 at different temperatures. (**A**) maximum specific growth rate as a function of temperature. Three independent experiments were performed for both strains at different temperatures. *the pre-culture of TM29 was incubated at 37 °C; (**B**) growth curves at 30 °C and 38 °C. The standard deviations were calculated from three independent experiments.

After a long-term thermal adaptation (860 generations), TM29 was isolated. This strain was able to grow at 40 °C, which is ten degrees above the normal optimal growth temperature of the parent strain (Fig. 1A). It was found that the optimum growth temperature of TM29 had been shifted from 30 °C to 36 °C, and the maximum specific growth rate (1.16 ± 0.02 h^−1^) was similar to the optimum of that of its parent, MG1363 (1.13 ± 0.03 h^−1^). Above 34 °C, the growth rate of TM29 was significantly higher than that of MG1363. The growth of MG1363 ceased at 39 °C, where no increase in cell density was observed after 24 hours of cultivation. TM29 could grow at 40 °C continuously, but this required the preculture to be incubated at slightly lower temperatures. Due to this reason, 40 °C was excluded for the following characterization. The growth curves for both MG1363 and TM29 are presented in Fig. 1B at two different temperatures. At 30 °C, MG1363 overall grew slightly faster than TM29. When the temperature was shifted to 38 °C, the exponential growth of TM29 was hardly affected, while MG1363 experienced a long lag-phase and slower growth during the exponential phase.

**Figure 2:**
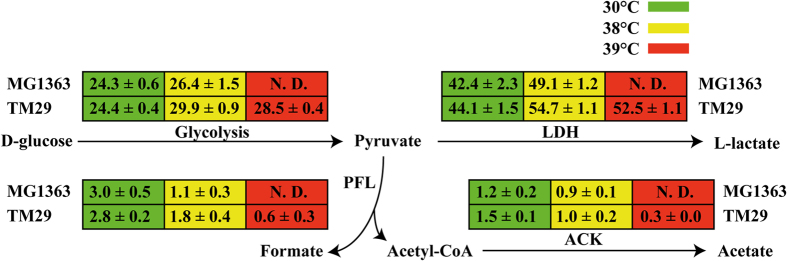
The specific consumption rate of glucose and specific production rate of lactate, formate and acetate at different temperatures in MG1363 and TM29. The unit of flux was mmol per hour per gram cell dry weight. N. D., not determined (no growth) LDH, lactate dehydrogenase; PFL, pyruvate-formate lyase; ACK, acetate kinase.

In Fig. 2, the specific glucose consumption and product formation rates can be seen. The glucose and lactate fluxes increased with temperature for both MG1363 and TM29, but the increase was larger for TM29, that had an approximately 12% higher flux at 38 °C than MG1363. It was also observed that the mixed-acid products formate and acetate were formed in smaller amounts as the temperature increased. At 39 °C, even though the specific production rate of lactate and maximum specific growth rate of TM29 were slightly reduced compared to those at 38 °C, the production rates of the by-products such as acetate and formate significantly declined.

**Figure 3:**
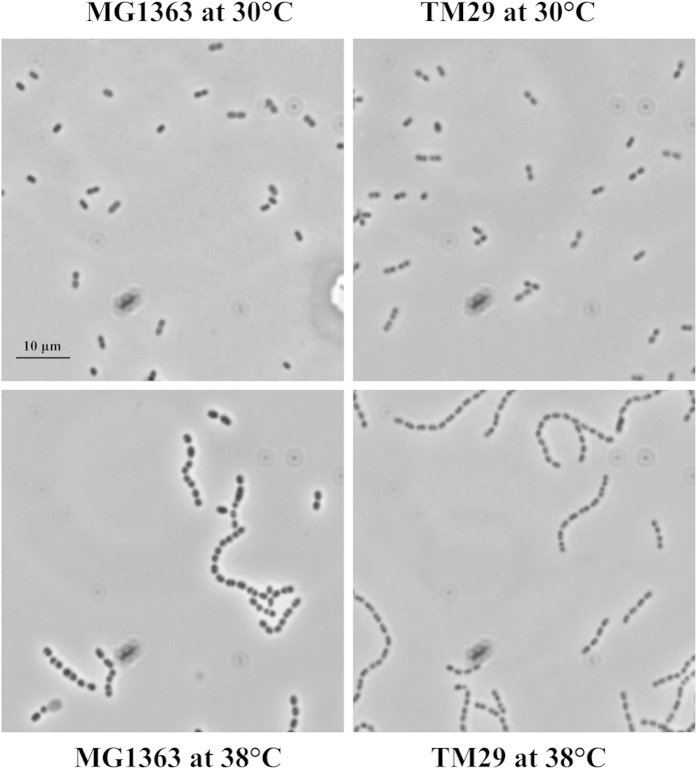
Comparison of cell size between MG1363 and TM29 at different temperatures using a phase-contrast microcope.

Another intriguing observation was that the cell dry weight to OD_600_ ratio decreased with increasing temperature for MG1363, but not for TM29 (Figure S1). Phase-contrast imaging revealed swelling of MG1363 at high temperatures whereas TM29 remained unaffected (Fig. 3).

### Transcriptional analysis of MG1363 and TM29 at different temperatures using DNA microarray

To compare the effect of temperature and mutations on global transcription, a transcriptional analysis was carried out using single-channel microarrays. We used two different comparisons for the significantly differentially expressed genes (fold change ≥1.5; adjust p-value ≤0.05): the response to the elevated growth temperature of MG1363 and TM29 respectively (38 °C vs. 30 °C) and the difference in global transcription between MG1363 and TM29 at high temperatures (38 °C).

When the transcriptional profiles were compared for the individual strains, a total of 758 and 650 genes were found to be differentially expressed in MG1363 and TM29, respectively (38 °C vs. 30 °C) (Table S1 & S2). We found that several heat shock proteins were up-regulated, and this occurred in both MG1363 and TM29. Among these genes, the *clpB,C,E* and *P* are involved in degrading misfolded proteins under stress conditions^19^. The *dnaK*, *grpE*, *groES* and *groEL* encode chaperones that assist refolding of proteins^12^,^13^, and the *hrcA* encodes a heat shock regulator^20^. The *arcA*, *B*, *C1*, *C2*, *D1*, which are involved in the arginine deiminase (ADI) pathway, were also up-regulated in both MG1363 and TM29. Moreover, in response to the elevated growth temperature, 18 regulators were up-regulated in both strains, such as *rmaA*, *rmaB*, *rmaX*, *rmaI*, *rmeB* and *rmeC*. In MG1363 an additional 14 specific regulators and 4 two-component signal transduction regulation genes *llrD*, *llrB*, *kinD*, and *kinE* were up-regulated. It was also noticed that, the riboflavin biosynthesis operon (*ribA*, *B*, *D*, *H*)^21^ was up-regulated, but only in MG1363. Although the higher growth temperature led to a higher glycolytic activity, none of the glycolytic genes were found to be differentially expressed in either MG1363 or TM29. Most of the genes found to be down-regulated in MG1363 and TM29, had a putative status.

When the transcriptional profiles were compared between the two strains at 38 °C (TM29 vs. MG1363), 297 and 271 genes were found to be up-regulated and down-regulated, respectively (Table S3). In order to clarify the function of these differentially expressed genes, gene functional classification by DAVID^22^ was applied using GO terms (Biological Process). Only one term was over-represented, namely “transport” (41 genes; *p*-value = 3.4E-5) for the up-regulated genes, and no terms were found over-represented for the down-regulated genes. In “transport”, most of the genes were found to encode diverse carbohydrate, amino acid and ion transporters (Table 1).

### Sequencing the genome of the mutant

In order to identify the mutations responsible for the thermal tolerance of TM29, its genome was sequenced. By comparing to the published *L. lactis* MG1363 sequence^23^,^24^ it was possible to determine all the mutations, which are listed in Table 2. A total of 15 mutations, consisting of 13 single-nucleotide polymorphisms (SNP), 1 single-nucleotide indel (DIP) and one large deletion (11K bases), were identified. The large deletion was found to be a fragment consisting of 10 genes (*llmg_1349–llmg_1358*), and it was flanked by two imperfectly matched DNA repeats (reference position: 1321034–1322303; 1333535–1334779, 1249 bp; 99% identity). Sanger sequencing confirmed that the excision had occurred as a consequence of homologous recombination between these repeats.

Most single-base mutations occurred in protein coding regions, but two intergenic ones were also observed. One intergenic mutation (Reference position: 403714) located in the CIRCE (Controlling Inverted Repeat of Chaperone Expression) element, a binding site of repressor proteins preceding the *groESL* operon^25^, and the other (Reference position: 1164619) preceded the CDS of RibU, a riboflavin transporter, and this mutation was located in an uncharacterized region upstream the ribosome binding site^26^. One intriguing observation was that mutations in *rpoC*, encoding the RNA polymerase *β*’ subunit, occurred twice within 200 bp on the chromosome.

### Assessing the contribution of individual mutations to tolerance at elevated temperatures

**Figure 4:**
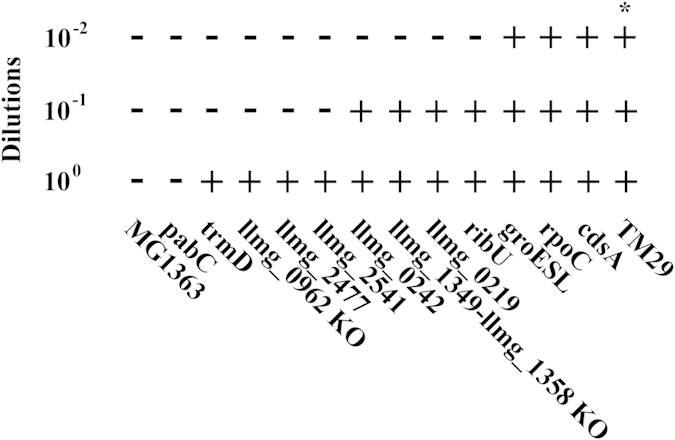
Growth performance of the allele replaced mutants, and the knockout mutants at 39 °C. Growth performance was assessed by occurrence of growth in serially diluting cultures with a pre-determined number of cells at the beginning, and registering growth after 48 hours of incubation at 39 °C. The test was repeated for four times. 10^0^, 10^−1^ and 10^−2^ represents 0, 10 and 100 times’ diluted cultures respectively. Occurrence of growth in different dilutions was indicated by “+”, and no growth was indicated by “−”. The detailed procedure is described in the Methods. *Growth occurred in the 10^−2^ dilution within 24 hours.

Genome sequencing and microarray analysis revealed the changes in the mutant at a genomic and transcriptional level, but did not provide a direct link between these and the improved fitness at elevated temperatures. To analyse the contribution of the individual mutations, each of them was introduced into MG1363, and the detailed genetic backgrounds of these strains are listed in Table S4. As some of these mutants containing the single mutation exhibited unstable growth at 39 °C, the thermal tolerance was, instead, assessed by a growth assay involving serially diluted cultures (10 times’ dilutions), and MG1363 and TM29 were included as benchmarks. Figure 4 shows that the occurrence of growth for each allelic replaced mutant at 39 °C, where no growth was observed for MG1363 and the *pabC* mutant in the lowest dilution 10^0^. Most of the other mutations were found to improve the thermal tolerance of MG1363 at 39 °C, where growth occurred in the dilution 10^−1^ or 10^−2^ (10 times and 100 times diluted compared to 10 °). Especially, the mutations in *cdsA* and *rpoC* and the mutation preceding *groESL* had a significant impact on the thermal tolerance compared to the other mutations, and these mutants enabled growth in the 10^−2^ dilution after 48 hours. However, none of these single mutations were able to render MG1363 as tolerant as TM29, and the growth of TM29 occurred in the 10^−2^ dilution within 24 hours.

### The effect of the SNPs preceding *groESL* and *ribU*

**Figure 5:**
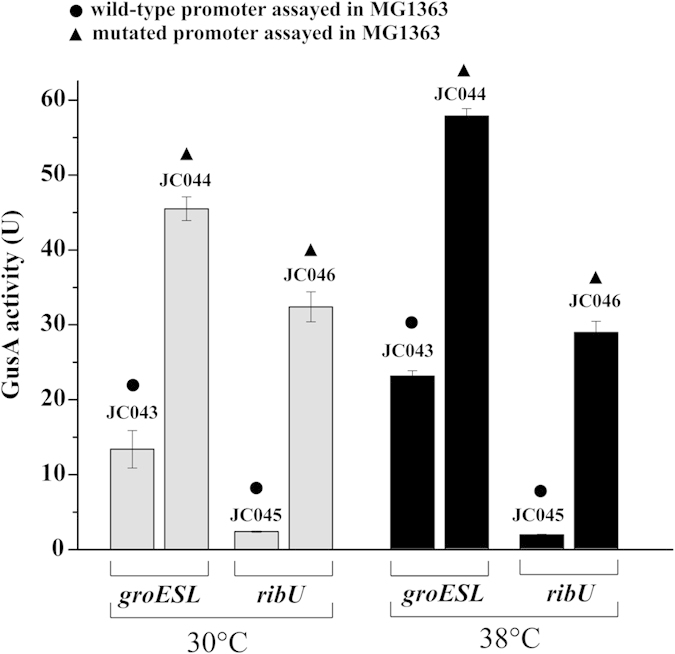
Assessment of the strength of native and mutated *groESL* and *ribU* promoters at 30 °C and 38 °C using *gusA* transcriptional fusions. The results are based on three biological replicates. The strain numbers are referred in Table S4.

Two SNPs occurred just upstream the CDS of *groESL* and *ribU*, and these were suspected to alter gene expression. To substantiate this, the wild-type and mutated promoters were fused to *gusA*, and β-glucuronidase activities determined as a measure for promoter strength. Figure 5 shows that the presence of the mutation upstream *groESL* resulted in a more than two-fold higher expression in an MG1363 background at both 30 °C and 38 °C when compared to the wild-type promoter.

**Figure 6:**
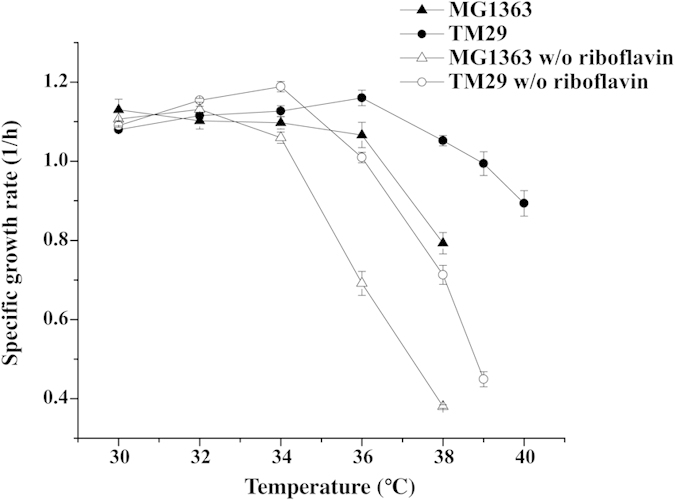
The maximum specific growth rate as a function of temperature for MG1363 and TM29 in medium with or without riboflavin.

Similarly, the mutation upstream *ribU* resulted in six-times over-expression at both low and high temperatures. Riboflavin is one of the prototrophic nutrients, which is generally not required for growth of *L. lactis*^21^. However, the situation could be different at elevated temperatures. In order to investigate whether riboflavin is important for growth at elevated temperatures, both strains were grown in medium with or without riboflavin at different temperatures. Figure 6 shows that the growth of both MG1363 and TM29 were significantly reduced above 34 °C without riboflavin, but TM29 was still able to grow faster than MG1363.

### The mutations in *rpoC* affect global transcription and the fatty acid composition

Mutations in *rpoC* have previously been reported to result in numerous changes to the transcriptional profile in other organisms, and could in principle affect a variety of phenotypes^27^,^28^. To determine whether the *rpoC* mutations obtained in this study had the same pleiotropic effect, the effect of the *rpoC* mutations on expression of several of the transporter genes, that were found to be up-regulated in TM29, was examined in a wild-type background (MG1363 with the *rpoC* mutations = JC030) using RT-qPCR. The outcome can be seen in Table 3. It can clearly be seen that several of these genes are up-regulated in both TM29 and JC030 at 38 °C when compared to MG1363 at 38 °C, thus demonstrating the pleiotropic effect of the *rpoC* mutations under these conditions.

**Figure 7:**
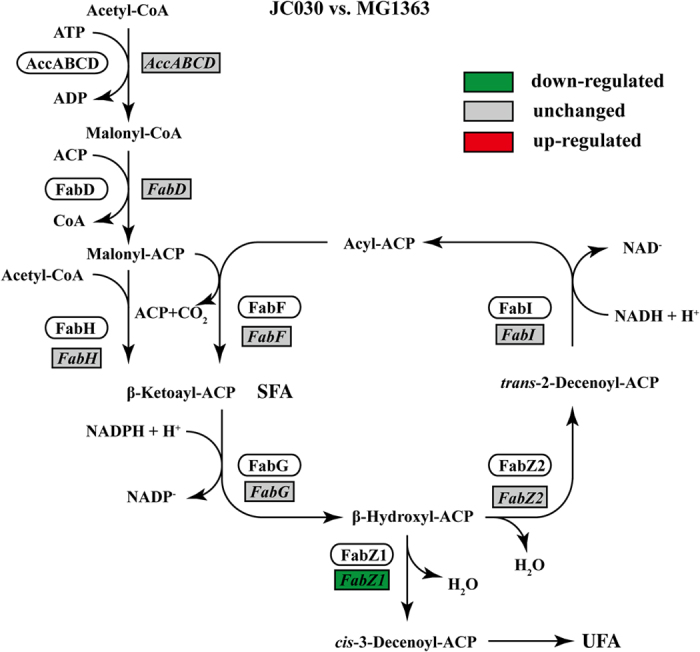
Change in expression level of genes in the fatty acid biosynthesis cluster between the *rpoC* allele replaced mutant and MG1363 at 38 °C (JC030 vs. MG1363). For each condition, three independent biological samples were used. The metabolism pathway was constructed based on the results from previous studies^30^,^31^. ACP, acyl carrier protein; SFA, saturated fatty acid; UFA, unsaturated fatty acid.

A higher proportion of saturated fatty acids in the cell membrane is known to result in a reduced proton permeability, which is highly important for bacteria thriving at high temperatures^29^. For this reason the cell membrane fatty acid composition was determined in MG1363 and TM29. A comparison of cell membrane fatty acid composition at 38 °C revealed significantly lower amounts of unsaturated fatty acids (UFA) in TM29 when compared to MG1363 (Table 4). The difference was found to be mainly due to the presence of less C18:1 (unsaturated) and more C14:0 and C16:0 (saturated) fatty acids in the mutant. The fatty acid composition was further determined for the *rpoC* mutant, JC030, because of its high thermal tolerance and because the pleiotropic nature of *rpoC* mutations in principle could affect gene expression in fatty acid metabolism^27^,^28^. Table 4 shows that the mutations in *rpoC* were able to alter the fatty acid composition in MG1363. Among the gene cluster involved in fatty acid metabolism, RT-qPCR analysis revealed that the mutations in *rpoC* caused a significant down-regulation of *fabZ1* (1.5 fold, *p*-value = 0.000), which encodes a bifunctional dehydratase/isomerase in the fatty acid metabolism in *L. lactis*^30^,^31^, in the mutant, while the other genes were not affected (Fig. 7). FabZ1 catalyses the conversion of β-hydroxyl-ACP into *cis*-3-decenoyl-ACP, which is the precursor for elongation of unsaturated fatty acids.

## Discussion

In this study we successfully isolated a thermo-tolerant *L. lactis* mutant, TM29, using ALE. We observed in this study that a high fermentation temperature could result in faster lactate production and less formation of formate and acetate, an effect that has been observed previously^8^,^9^,^32^. Our results demonstrate that lactate production in *L. lactis* can be further improved through thermal adaptation as the growth rate is increased.

At 38 °C the mutant produced lactate 12% faster than its parent MG1363. Previous studies have suggested that additional energy requirements for maintenance and enhanced interactions between enzymes and metabolites at high temperatures, rather than self-regulation of glycolysis, could be the cause of this increased metabolic flux^8^,^33^,^34^. Our results are in agreement with this, as the expression of the glycolytic genes was maintained at the same level irrespective of temperature, and no mutations that could explain the increased glycolytic flux at high temperatures were revealed by genome sequencing.

Besides affecting glycolysis, a high temperature also can affect macromolecular assembling, cell membrane stability and other biological processes, which could hamper growth^8^,^9^,^34^. We found that growth rate and glycolysis were uncoupled at high temperatures, and a significantly reduced growth rate was found for MG1363 above 36 °C. This affected the volumetric productivity of lactate negatively, which has been observed previously^35^. In contrast, TM29 grew nearly twice as fast as its parent MG1363 at 38 °C, and the maximum growth temperature, the lactate flux and the end-product composition were also significantly improved. TM29 is capable of growing at 39 °C and 40 °C where growth of MG1363 ceased at 39 °C. However, pre-culturing at a lower temperature was required for TM29 to grow at 40 °C. This pre-incubation probably allows for growth and survival at a non-permissive temperature based on induction of molecular chaperones prior to the elevation of temperature^12^. The acquisition of this phenotype was most likely caused by the ALE, where the temperature was gradually increased.

Maintaining membrane integrity and intracellular environment is essential for normal cell functions, and the swelling that we observe for MG1363 at high temperatures (Fig. 3) combined with reduced growth and a large ATP demand clearly demonstrate that MG1363 is struggling with this. This observation can explain why a high concentrations of osmolytes improve heat tolerance^36^,^37^, since a high concentration of osmolytes would prevent swelling at high temperatures, and thus allow the cells to maintain vital functions. The ability of TM29 to maintain its cell size correlates well with the acquisition of thermal tolerance, but the mechanism involved is still unknown.

Genetically, a total of 14 single nucleotide variations (SNV) and 1 gene deletion were identified in TM29 after 860-generations of adaptation at high temperatures. This corresponds to a SNV rate of 1.63 per 100 generations which is similar to what has previously been reported for adaptations under other stress conditions (1.13 to 2.13 per 100 generations)^38^. A few intergenic mutations, which resulted in alteration in gene expression, were found to play important roles for the thermal tolerance in the mutant.

One interesting mutation significantly improving the thermal tolerance at 39 °C was a single-base substitution in one of the palindromic arms of the CIRCE element (TTAGTACTC-N9-GAGTGCTAA) upstream *groESL*, which encodes a chaperonin system. The CIRCE element, which is a negative *cis*-acting element that is involved in regulation of several heat shock genes via the repressor protein HrcA, is prevalent in Gram-positive bacteria^39^. Site-specific single-base substitution in the arms of CIRCE elements or removal of the CIRCE result in a significant up-regulation of downstream chaperone genes^25^,^40^. This is exactly what we observed in this study, where the substitution C → T (Reference position: 403714) caused a more than two-times overexpression of the genes *groESL* in MG1363. Chaperones are important for thermal tolerance, as they assist in refolding or degradation of the misfolded proteins that appear more frequently at high temperatures. In a thermally adapted *E .coli* strain, which was able to grow at 3 °C above the normal maximum, mutations caused an extreme up-regulation of GroESL (16-fold overexpression), which was central to the thermal tolerance^41^. However, in our study we find a more modest overexpression of GroESL, which only contributes to a part of the thermal tolerance. This difference could be the result of different regulatory mechanisms between *E. coli* and *L. lactis*, and where *L. lactis* relies on negative control, and *E. coli* uses several sigma factors that are involved in positive control mechanisms^39^.

In thermal adaptation, membrane transport processes are expected to be of considerable importance. *L. lactis*, and in particular the dairy isolates, rely heavily on various transport processes, as they are heterotrophic for various nutrients such as amino acids and vitamins^42^. Regulatory mutations that cause over-expression of substrate specific transporters have been reported in previous studies of *L. lactis* strains adapted to different environments^43^. We found another interesting mutation upstream the gene encoding the riboflavin transporter (*ribU*) that resulted in constant over-expression. The mutation was located in an uncharacterized sequence that is between the ribosome-binding site and the RFN element (FMN riboswitch) of *ribU*^26^. We found that the riboflavin biosynthesis operon was up-regulated in MG1363 at high temperatures, which could indicate riboflavin starvation (Table S3). Recently we reported^44^ that *L. lactis* starves for riboflavin at high temperatures, and that this results in an inefficient biosynthesis of FAD affecting the FAD-dependent enzymes pyruvate dehydrogenase (PDH) and NADH oxidase (NOX). This then impacts on fatty acid biosynthesis and causes a general oxidative stress condition that has direct consequences for *L. lactis* in terms of hampered growth, in particular in the absence of riboflavin. When the riboflavin transporter RibU was overexpressed in *L. lactis*, biosynthesis of FAD was improved, which had a positive effect on the growth at high temperatures^44^. By comparing the growth of MG1363 and TM29 at different temperatures with or without riboflavin in the medium we were able to confirm the important role of this compound for growth at high temperatures (Fig. 6).

We also found two mutations in *rpoC*, encoding the RNA polymerase *β*’-subunit, that rendered a significant thermal tolerance to *L. lactis*. When these mutations were introduced in a wild-type background, growth at 39 °C was improved just as for the *groESL* mutation. Large-scale alterations to gene expression profiles are usually caused by genetic changes in a few essential transcriptional regulators. Mutations in *rpoC* have also, for example, been observed in other ALE studies conducted on different organisms^27^,^28^, where the mutations lead to altered transcription and adaptation to the new environment. In our case we were indeed able to demonstrate that many of the differentially expressed genes in TM29 were also differentially expressed in the strain where the *rpoC* mutations were introduced in a wild-type background (Table 3). Among these genes *arcD1* encodes the arginine/ornithine antiporter, which is involved in arginine deiminase (ADI) pathway^23^. Arginine is metabolized through the ADI pathway and is coupled to ATP production which could contribute to thermal tolerance by boosting the proton extrusion carried out by the F_0_F_1_—ATPase^45^, and it has previously been found that the ADI pathway is induced in LAB when the temperature is increased^46^. *GlcU* encodes the sole non-PTS glucose permease in *L. lactis*.^23^,^47^, and the up-regulation of *glcU* could lead to an improved uptake of glucose that is required to support the additional energy demand for maintenance at high temperatures^34^. *Llmg_1993* encodes an amino acid transporter^23^, and it is known that amino acid utilization is one of the factors that limit high-temperature growth of bacteria^48^. *Nha*, *pacL*, *cibO* and *cibQ* encode different ion transporters^23^, and ion transporters are probably important for heat stress response, as they are normally induced under thermal stress conditions^49^,^50^.

Moreover, it was found that the *rpoC* mutant had a significantly reduced amount of UFA in the cell membrane. This could lead to growth at high temperatures without an excessive increase in membrane fluidity and loss of membrane function. The *cis* double bond in UFA reduces acyl-chain packing in the membrane, and thereby decrease van der Wahl-interactions between the chains and the consequence is an increase in membrane fluidity^51^. The amount of fatty acids with the capacity to disturb acyl-chain packing in the cell membrane is therefore regarded as one of the crucial factors for growth at high temperatures. Since membranes with a high content of these are less capable of maintaining a low ion permeability at high temperatures they are less capable of maintaining the various essential ion gradients^52^. In *L. lactis*, the involvement of *fabZ1* in fatty acid desaturation was recently suggested^30^. Among all the gene clusters in fatty acid biosynthesis, *fabZ1* was uniquely down-regulated by the mutations in *rpoC* (Fig. 7), which supports the observed phenotype.

The pleiotropic effects of the *rpoC* mutations demonstrate that RNA polymerase could serve as a potential regulatory hub, as suggested by Conrad, T. M. *et al.*^27^, in rewiring the transcription network to heat stress in *L. lactis*. Such regulation is not well understood in *L. lactis*, and to gain further understanding a global transcriptional analysis of strains with mutated *rpoC* in a wild-type background could be helpful.

One causal variation was found to be a large gene rearrangement, which resulted in the deletion of an operon consisting of genes from *llmg_1349* to *llmg_1358*. Large gene rearrangements usually involve transposable elements (Insertion sequence; IS) that generate insertions and deletions through recombination between homologous elements in *L. lactis* during ALE^53^. The cause of excision in this case was two large flanking DNA repeats, which, however, most likely resulted from the sex factor promoted gene excision^54^. Four genes in this operon (*llmg_1350*-*llmg_1353*) encode putative tellurium resistance proteins and cAMP-binding protein CABP1, and the others encode mostly hypothetical ones. Because of the scarcity of prior researches, it is difficult to find the connection between inactivation of tellurium resistance and heat tolerance. Recently, it has been reported that mutations in the gene *GdpP*, encoding the c-di-AMP phosphodiesterase, render *L. lactis* MG1363 the ability to grow at high temperatures, which indicates the probable involvement of such a secondary messenger in stress response^55^.

The mutation in *cdsA* significantly improved the thermal tolerance as did the mutations that affected *groESL* and *rpoC* (Fig. 4). CDP diglyceride synthetase catalyzes the synthesis of CDP-diacylglycerol (CDP-DG) from cytidine triphosphate and phosphatidate in both prokaryotes and eukaryotes. CDP-DG is the important intermediate at the branch point for synthesis of glycerophospholipids such as phosphatidylethanolamine (PE), phosphatidylglycerol (PG), and cardiolipin (CL)^56^. Thus, *cdsA* could limit the overall metabolism of head groups rather that alter the composition. It has been found that mutations in enzymes catalyzing steps after CdsA can result in both a thermally sensitive metabolism and have a general effect on growth^57^,^58^,^59^. Although thermal sensitivity of CdsA has not been reported, considering the central role of this enzyme in the glycerophospholipid metabolism, a thermally stable CdsA could be important for high temperature growth of *L. lactis*.

For the other genes, which play minor roles in the thermal tolerance, *llmg_2477* and *llmg_2541* encodes lysine specific permease and cation transporting ATPase, respectively. They could be involved in the aforementioned transport process for nutrients supplement and ion homeostasis. The role of *TrmD*, *llmg_0962*, *llmg_0242* remains elusive due to lack of prior knowledge or unclear annotation.

In this study, we have successfully demonstrated that thermo-tolerant *L. lactis* mutants can be isolated through ALE. The mutant had interesting and industrially relevant properties such as improved growth rate, higher specific production rate of lactate and less formation of formate and acetate. The application of NGS and allelic replacement allowed us to identify the mutations responsible for the phenotype of the mutant at high temperatures. The mutations identified were found to influence molecular chaperones, cell membrane, and nutrient and iron transporters. None of these single mutations were able to completely reproduce the thermo-tolerant phenotype observed for the end-point mutant, which suggests that the decisive factors dictating thermal tolerance in *L. lactis* are multiple.

## Methods

### Bacterial strains and plasmids.

The bacterial strains and plasmids used in this study are listed in Table S4.

### Growth media and growth conditions

*E. coli* strains were grown aerobically in Luria-Bertani (LB) broth^60^. *L. lactis* was cultivated in chemically defined SALN medium (pH 7.0) with an additional 40 mM of MOPS^44^, or M17^61^ with 0.2% glucose. The end pH of *L. lactis* strain growing in the SALN medium is usually around 6.5 due to the addition of MOPS and limited amount of glucose. Growth experiments were performed in filled 300 ml glass bottles with screw caps. Optical density was measured at OD_600_ on a UV-1800 UV-Vis spectrophotometer (Shimadzu). Experiments were started at OD_600_ = 0.001 using overnight cultures growing exponentially at same temperatures as growth experiments. Slow magnetic stirring was used to keep the cultures homogeneous. The maximum specific growth rates (μ_max_) were calculated using optical density data, which were collected during exponential growth. For measurement of growth curves, overnight cultures were 1/1000 time’s diluted into fresh medium. 200 μl cultures were transferred into a honeycomb 2 plate (Oy Growth Curves Ab Ltd). Incubation and OD_600_ measurement were performed on a Bioscreen-C Automated Growth Curves Analysis System (Oy Growth Curves Ab Ltd). Shaking was performed prior to each measurement for keeping homogeneity.

### Metabolite determination and flux calculations

The concentration of glucose, lactate, formate and acetate were determined on an Ultimate 3000 high-pressure liquid chromatography system (Dionex) equipped with an Aminex HPX-87H column (Bio-Rad) and a Shodex RI-101 detector (Showa Denko K.K.). The column oven temperature was set at 30 °C and a mobile phase of 5 mM H_2_SO_4_ with a flow rate of 0.5 ml/min was used. Samples collected during exponential growth (OD_600_ = 0.1, 0.2, 0.3, 0.4, 0.5 and 0.6) were analysed. For flux calculations, cell density was correlated to the corresponding cell mass of the wild-type and mutant strain at different temperatures.

### Procedure for adaptive evolution

The evolution was started from *Lactococcus lactis* MG1363^18^ and conducted using a serial-transfer regime. Cultures were statically incubated in test tubes in a temperature controlled water bath, and optical density was visually followed. When the culture entered the stationary phase, one ml culture was transferred into a new test tube with 9 ml fresh medium, which corresponds to 3.32-generations of growth in each tube. The procedure was also illustrated in Figure S2. The transfer frequency varied from several hours to more than one day depending on growth rate, and the number of transfers was noted down. Each week, a culture sample was saved in 25% glycerol at −80 °C in order to track the evolution. The evolution temperature was adjusted in a stepwise manner. Cells were adapted at 38 °C for 120 generations, 39 °C for 345 generations and 40 °C 395 generations. In total, cells were adapted at high temperatures for 860 generations. Culture from the final tube was streaked on a plate, one single colony was isolated, and the clone was designated as TM29.

### Microarray analysis

Custom single-channel DNA microarrays for *L. lactis* MG1363 with probes for all annotated genes in the published sequence were designed using OligoWiz^62^ and chips were ordered from Agilent.

Cells were cultivated in bottles under the same condition as the growth experiment. Four independent biological samples were harvested during exponential growth (OD_600_ = 0.3) and mixed with 10% of an ice-cold “stop solution” (5% equilibrated phenol, pH = 7.4, in ethanol) and centrifuged at 6000 g at 4 °C for 10 min. The supernatant was discarded and pellet was washed with ice-cold distilled water once and stored at −80 °C.

For RNA isolation, cells were mixed with 200 μl Solution I (0.3 M sucrose and 0.01 M NaAc, pH 4.8) and 200 μl Solution II (2% SDS and 0.01 M NaAc, pH4.8). Then, 400 μl hot phenol (equilibrated with NaAc, pH 4.8) was added into the mixture and disruption was performed using acid-washed glass beads (106 μm) (Sigma) with a FastPrep (MP Biomedicals). The lysate was centrifuged at room temperature and the water phase was extracted by phenol/NaAc twice followed by phenol/NaAc/chloroform (phenol/NaAc:chloroform = 1:1) once. RNA was precipitated by ethanol and dissolved in DEPC-treated water. The integrity of RNA was determined by a 2100 BioAnalzyer (Agilent).

cDNA synthesis, Cy3 labeling and purification were performed using a Stratagene FairPlay III Microarray Labeling Kit (Agilent) with 5 μg total RNA. A NanoDrop 1000 (Agilent) was used for quantitating cDNA. Hybridization was performed in an Agilent Hybridization Chamber (G2534A) for 17 h at 65 °C. Slides were scanned on an Agilent Microarray Scanner (G2565BA). Raw data were extracted by an Agilent Features Extraction Software according to the manufacture’s specifications.

Data processing and analysis were performed using R^63^ and Bioconductor^64^. To select differentially expressed genes, two-sample testing was performed using linear models and empirical Bayes methods from the LIMMA package^65^. Significantly differentially expressed genes (adjusted *p*-value < 0.05; fold-change >1.5) were chosen for GO analysis. GO analysis was carried out by terms of biological progress (GOTerm_BP_3, Benjamini adjusted P-Value ≤ 0.05) using DAVID^66^ and the total GI numbers (2434 CDSs) *of L. lactis* MG1363 were used as the reference. The microarray data have been deposited in the Gene Expression Omnibus (GEO) database (Series entry: GSE27698).

### RT-qPCR

First strand cDNA was synthesized from 3 μg total RNA using the Maxima First Strand cDNA Synthsis Kit (Thermo Fisher Scientific) according to the manufacture’s specifications. Primers and probes (Table S5) for RT-qPCR were designed using PrimerQuest (Integrated DNA Technologies). The RT-qPCR reaction mixture contains 1X Maxima Probe/ROX qPCR Master Mix (Thermo Fisher Scientific), 500 nM primers, 250 nm probe and 10 ng cDNA template. The *pheS and alaS* housekeeping genes were used as the references. For each condition, three biologically independent samples were used and the reactions were carried out in triplicate on an Mx3005P qPCR system (Agilent). Cycling conditions for all amplifications were one cycle of 95 °C for 10 min and 40 cycles of 95 °C for 15 s, 60 °C for 30 s, and 72 °C for 30 s. For the RT-qPCR data, an average cycle threshold (*C*_*T*_) value was calculated from the triplicate reactions. Amplification efficiency and statistical analysis were performed using REST 2009^67^.

### Genome sequencing and mutation discovery

Genomic DNA of the mutant was purified using DNeasy Blood & Tissue Kit (Qiagen) and the quality was checked by DNA electrophoresis and NanoDrop 1000 (Thermo Fisher Scientific) analysis. Genome sequencing was performed by Macrogen. The procedure described briefly: 2 μg genomic DNA was randomly sheared using a nebulizer (Illumina) and the ends were repaired using polynucleotide kinase and Klenow enzyme. The 5′-ends of the DNA fragments were phosphorylated and a single adenine base was added to the 3′-ends using Klenow exo^+^ (Illumina). Following ligation of a pair of Illumina adaptors to the repaired ends, the DNA was amplified in 10 cycles, using adaptor primers (Illumina), and fragments of around 150 bp were isolated using agarose gel electrophoresis. Sequencing libraries were quantified with a 2100 BioAnalyzer DNA 1000 chip (Agilent) as well as the Picogreen fluorescence assay (Invitrogen). Cluster generations were performed on an Illumina cluster station using 11 pmol of sequencing libraries. A total of 38 cycles of sequencing were carried out using the Illumina Genome Analyzer IIx system according to the manufacturer’s specifications. CLC Genomics Workbench was used for mapping the reads, SNP and DIP detection and identification of genomic rearrangement using the published genome sequence of *L. Lactis* MG1363^23^,^24^ as the reference.

### Allelic replacement for allele containing SNP and gene knockout

A non-replicating *oroP* containing integration vector was used for allelic replacement and gene knockout^68^. SNPs position contained homologous sequences or upstream and downstream regions of target genes for knockout were amplified from the chromosomal DNA of MG1363 and TM29 and cloned onto pCS1966 using restriction enzymes. The resulting plasmids were transformed into *L. lactis* and successful integrations were selected on GM17 plates using erythromycin. Counter-selection was performed on SAL (SA-medium with lipoic acid) plates containing 50 μg/ml 5-fluoroorotic acid. Successful allelic replacement or knockout was confirmed by Sanger Sequencing. The primers used in this study are listed in Table S6. Chromosomal DNA isolation, DNA manipulation and transformation for *E. coli* and *L. lactis* were performed as previously described^69^.

### Test of thermal tolerance in serial dilutions

The serial-dilution test for thermal tolerance was conducted according to the previous report, where liquid medium was used in this study instead of agar plates^55^. Approximately 2E10^4^ cells, which were from an overnight culture, were inoculated into a 1.5 ml centrifuge tube containing 500 μL medium, which results in an initial OD_600_ = 0.0002. A series of 10-times dilutions were carried out and resulted in tubes containing 10^−1^ and 10^−2^ times the cells in the first tube. Tubes were statically incubated at 39 °C for 48 hours. Afterwards, the tubes were centrifuged at 20,000 g for 1 min and occurrence of growth was determined by the presence of visible cell pellets. The procedure was also illustrated in Figure S3.

### β-Glucuronidase activity assay

β-Glucuronidase activities were determined by the procedure described by Miller^70^ and modified by Israelsen *et al.*^71^, except that para-nitro-β-glucuronic acid (Biosynth AG) was used as the substrate^72^. The activity is given in Miller units per mg cell dry weight and calculated as described by Miller^70^.

### Membrane fatty acid analysis

Cells were harvested during exponential growth and washed with distilled water twice. Pellets were resuspended and treated with 20 mg/ml lysozyme for 30 min at 30 °C. Cell lysis was performed in a French press (Thermo Fisher Scientific) at 20,000 psi at 4 °C twice for each sample. Lipid extraction from the homogenate was based on the Bligh & Dye-procedure. Briefly 0.8 volumes of the homogenates was mixed with 1 volume CHCl_3_ and 2 volumes MeOH, and extracted overnight at 4 °C. Phase-separation was induced by the addition of 1 volume 0.73% NaCl (aq) and 1 volume CHCl_3_, and the organic phase was retrieved after mixing and centrifugation of the sample. Fatty acids were trans-methylated in borontriflouride and fatty acid composition was analyzed as earlier described^73^. Fatty acid methylesters (FAME) were separated on a 50-m Supelco SP-2380 column (Sigma-Aldrich) in a HP 6890 gas chromatograph (GC), in split mode using He as carrier gas. GC settings were: injector temperature 250 °C, split ratio 20:1, carrier flow 1.2 mL/min, detector temperature 270 °C, air flow in detector 400 mL/min, hydrogen flow 30 mL/min. FAME were separated using a temperature program starting at 50 °C and rising to 140 °C at 30 °C/min; this temperature was kept for 2 min, hereafter the temperature was raised to 220 °C at 2 °C/min and the oven was kept at 220 °C for 5 min before the temperature was raised to 250 °C at 10 °C/min. The final temperature was kept for 17 min. FAME were identified using authentic standards (Nu-Check Prep).

## Additional Information

**How to cite this article**: Chen, J. *et al.* Adaptation of *Lactococcus lactis* to high growth temperature leads to a dramatic increase in acidification rate. *Sci. Rep.* **5**, 14199; doi: 10.1038/srep14199 (2015).

## Supplementary Material

Supplementary Information

## Figures and Tables

**Table 1:** The list of up-regulated transporter genes in the GO term “transport” (TM29 vs. MG1363 at 38 °C).

**Gene name**	**logFC**	**Adj.P.Val**	**Annotation**
*llmg_1747*	0.6	1.9E-02	Amino acid permease
*lmrC*	0.6	1.0E-03	Multidrug resistance protein C
*rgpC*	0.6	1.1E-04	Capsule polysaccharide export inner-membrane protein RgpC
*copA*	0.6	1.3E-02	Copper/potassium-transporting ATPase
*ptnD*	0.7	4.7E-02	PTS system, mannose-specific IID component
*glnP*	0.7	4.9E-04	Glutamine ABC transporter permease and substrate binding protein
*mntH*	0.7	1.4E-02	Putative proton-dependent manganese transporter group C beta
*oppC2*	0.7	9.8E-04	Oligopeptide transport system permease protein oppC2
*eriC*	0.7	2.1E-04	Putative chloride channel protein
*rbsD*	0.7	1.2E-04	D-ribose pyranase
*llmg_1086*	0.8	1.1E-03	Similar to cation (Calcium) transporting ATPase
*oppB2*	0.8	4.0E-04	Peptide transport system permease protein oppB2
*dinF*	0.8	3.5E-05	Damage-inducible protein DinF
*llmg_1016*	0.8	1.7E-05	Cationic transporter
*secY*	0.8	3.7E-05	Protein translocase subunit SecY
*llmg_2541*	0.8	2.3E-05	Cation transporting ATPase
*rbsC*	0.9	1.9E-02	Ribose transport system permease protein RbsC
*lysQ*	0.9	3.2E-05	Amino-acid permease lysQ
*pacL*	1.0	3.9E-04	Cation-transporting ATPase, E1-E2 family
*cydD*	1.0	9.7E-07	Cytochrome D ABC transporter ATP binding and permease protein
*llmg_0375*	1.0	5.4E-05	Amino acid permease
*cydC*	1.1	2.7E-06	Cytochrome D ABC transporter ATP binding and permease protein
*llmg_0268*	1.1	3.5E-06	ABC transporter ATP binding protein
*pmrA*	1.1	2.3E-05	Multidrug resistance efflux pump
*llmg_0989*	1.1	8.8E-06	ABC transporter ATP binding and permease protein
*llmg_0870*	1.1	1.6E-05	Transporter
*ypbC*	1.2	5.3E-05	Putative membrane protein
*llmg_2011*	1.2	3.8E-05	Putative amino acid permease
*pbuX*	1.3	2.3E-05	Xanthine/uracil permease
*glcU*	1.3	2.2E-07	Putative glucose uptake protein glcU
*llmg_0269*	1.3	5.3E-05	ABC transporter ATP-binding and permease protein
*cbiO*	1.3	5.7E-08	Putative cobalt ABC transporter ATP-binding protein
*comGB*	1.3	1.6E-07	Putative competence protein ComGB
*llmg_1474*	1.4	9.9E-06	Putative voltage gated chloride channel
*comGC*	1.4	2.6E-08	Putative competence protein ComGC
*llmg_1426*	1.5	3.0E-06	Sucrose-specific PTS system IIBC component
*comGA*	1.6	5.5E-09	Putative competence protein ComGA
*cbiQ*	1.6	3.3E-09	Putative cobalt ABC transporter permease protein
*arcD1*	1.7	4.7E-07	Arginine/ornithine antiporter
*nha*	2.0	3.9E-06	Na^+^/H^+^ antiporter
*llmg_1993*	2.1	1.8E-07	Hypothetical transporter

**Table 2:** Mutations identified in the temperature-adapted mutant TM29.

**Reference**^a^	**Variation**^b^	**Changed**	**Gene**	**Protein**	**Amino acid**
208637	SNP	A→G	*llmg_0219*	Putative membrane protein	Thr→Ala
230889	SNP	G→A	*llmg_0242*	Probable transcriptional regulatory protein	Gly→Arg
403714	SNP	C→T	Intergenic		
598835	SNP	C→T	*pabC*	Putative aminodeoxychorismate lyase	Pro→Ser
905914	SNP	G→A	*trmD*	tRNA (guanine-N(1)-)-methyltransferase	Val→Ile
927877	SNP	T→A	*llmg_0962*	Transcriptional regulator, araC family	Cys→Stop
945240	DIP	—→G	*tnp904*	Transposase for insertion sequence IS904F	Frameshift
1078271	SNP	A→G	*pfk*	6-phosphofructokinase	Silent mutation
1164619	SNP	C→G	Intergenic		
1966122	SNP	C→T	*rpoC*	DNA-directed RNA polymerase subunit beta′	Gly→Arg
1966323	SNP	C→T	*rpoC*	DNA-directed RNA polymerase subunit beta′	Glu→Lys
2358022	SNP	G→A	*cdsA*	Phosphatidate cytidylyltransferase	Pro→Leu
2434453	SNP	G→A	*llmg_2477*	Lysine specific permease	Ala→Ser
2497054	SNP	G→A	*llmg_2541*	Cation transporting ATPase	Ala→Thr
1322282-			*llmg_1349-*		
	DEL	—		—	—
1334514			*llmg_1358*		

^a^Reference position refers to the genome sequence of *L. lactis* MG1363 (GenBank accession # AM406671).

^b^SNP, single nucleotide polymorphism; DIP, deletion/insertion polymorphism; DEL, large deletion.

**Table 3:** List of representative up-regulated genes in both TM29 and JC030 compared with MG1363 at 38 °C.

**Gene**	**Expression ratio**^a^ **Microarray**	***p*-value**	**Expression ratio**^a^ **qPCR**	***p*-value**
	**TM29 vs. MG1363**		**JC030 vs. MG1363**	
*arcD1*	3.14	0.00	1.96	0.03
*cbiO*	2.48	0.00	1.39	0.03
*cbiQ*	3.10	0.00	1.39	0.03
*glcU*	2.45	0.00	1.36	0.03
*llmg_1993*	4.26	0.00	2.20	0.00
*nha*	3.86	0.00	2.23	0.00
*pacL*	2.00	0.00	3.16	0.00

^a^fold change.

**Table 4:** Cell membrane fatty acid composition for MG1363, TM29 and JC030 at high temperatures.

**Fatty acid**	**MG1363**	**TM29**	**JC030**
	**Composition%**		
C14:0	6.4 ± 0.2	10.4 ± 0.1	11 ± 0.4
C14:1	0.8 ± 0.0	0.6 ± 0.0	0.6 ± 0.1
C16:0	19.3 ± 1.2	25.7 ± 0.4	28.1 ± 0.3
C16:1	1.3 ± 0.0	0.8 ± 0.0	0.8 ± 0.0
C16:1Δ9	2.2 ± 0.1	1.9 ± 0.0	1.9 ± 0.1
C18:0*	1.4 ± 0.3	1.5 ± 0.0	1.6 ± 0.1
C18:1	49.8 ± 0.5	44.3 ± 0.1	38.1 ± 1.4
ΔC19:0	12.5 ± 0.1	5.8 ± 0.1	9.1 ± 0.2
Unknown	6.4 ± 0.7	9.0 ± 0.7	8.9 ± 1.1
Unsaturation	54.1 ± 0.4	47.6 ± 0.2	41.4 ± 1.6

^*^Except C18:0, the population means of each fatty acid were significantly different at the 0.05 level, which was tested by one-way ANOVA.
