# Supplementary material for: Adaptation of Lactococcus lactis to high growth temperature leads to a dramatic increase in acidification rate
Source: Sci Rep. 2015 Sep 21;5:14199. doi: 10.1038/srep14199 (PMC4585701; doi:10.1038/srep14199)
Supplement: Supplementary Information [file srep14199-s1.pdf]

Adaptation of *Lactococcus lactis* to high growth temperature leads to a dramatic increase in acidification rate

Jun Chen[1], Jing Shen[1], Lars Ingvar Hellgren[2], Peter Ruhdal Jensen[1], and Christian Solem[1*]

1. National Food Institute, Technical University of Denmark, DK-2800 Kgs. Lyngby, Denmark

2. Department of Systems Biology, Technical University of Denmark, DK-2800 Kgs. Lyngby, Denmark

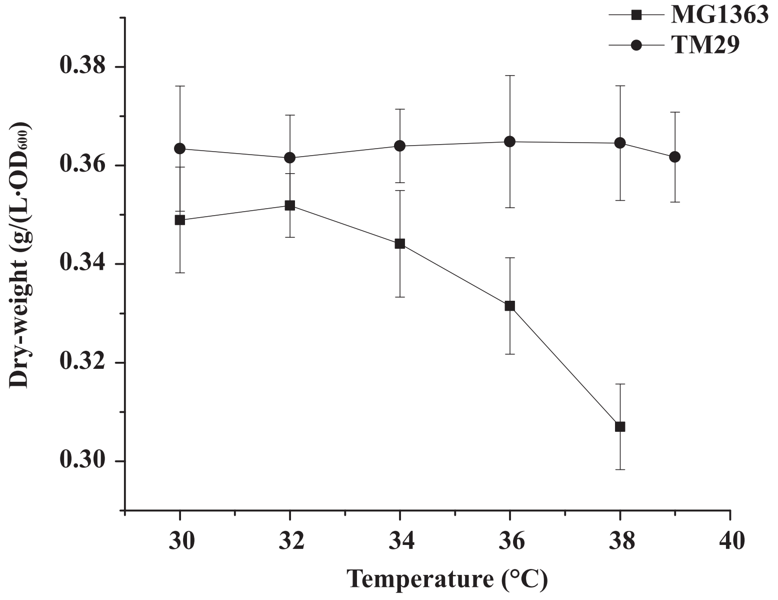

Figure S1. The cell dry-weight as a function of temperature for MG1363 and TM29.

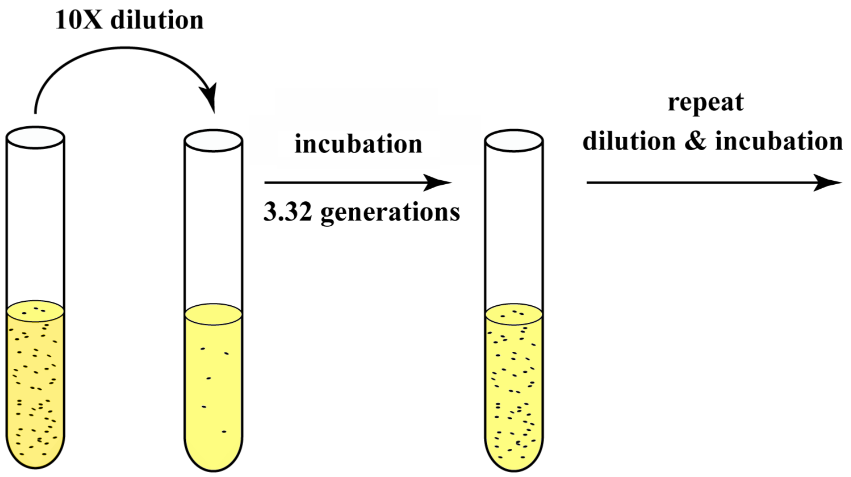

Figure S2. Illustration of procedure for adaptive laboratory evolution.

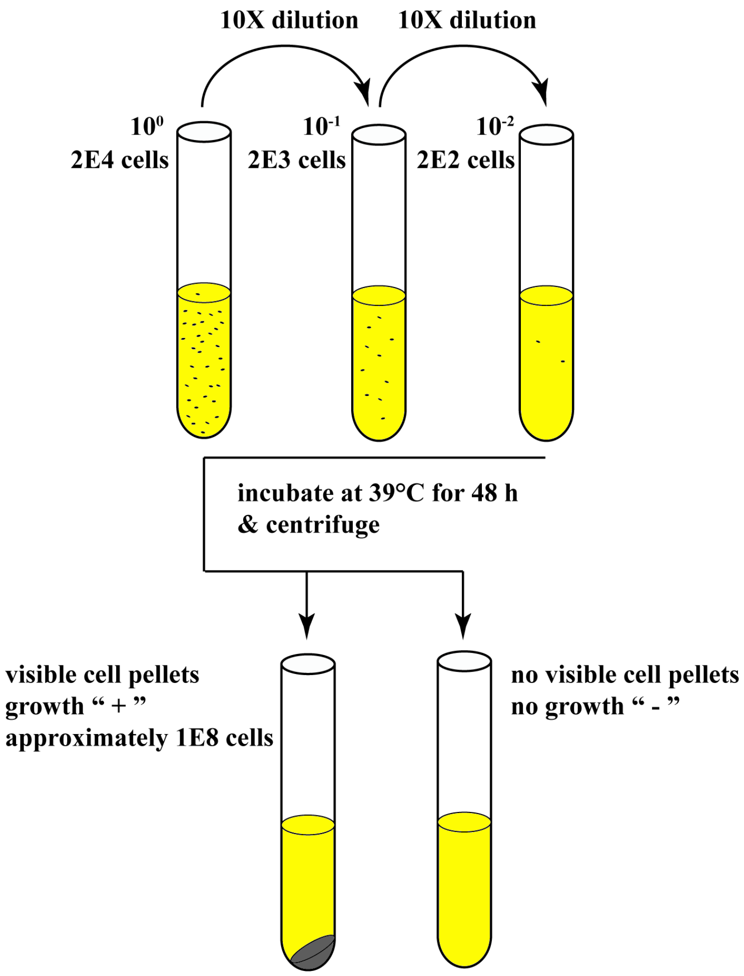

Figure S3. Illustration of procedure for serial-dilution experiment.

Table S1. Differentially expressed genes in MG1363 (38°C vs. 30°C)

Gene name	Protein name	logFC	adj.P.Val
amtB	Ammonium transporter AmtB	-3.33	1.51E-09
llmg_1104	Drug-export protein	-2.89	6.93E-11
glnB	Nitrogen regulatory protein P-II	-2.80	5.05E-10
llmg_1747	Amino acid permease	-2.39	2.38E-08
icd	Isocitrate dehydrogenase [NADP]	-2.33	9.23E-09
llmg_1397	Putative uncharacterized protein	-2.24	6.91E-11
llmg_1396	Putative uncharacterized protein	-2.20	3.05E-11
citB	Aconitate hydratase	-2.19	9.34E-10
llmg_1407	Putative uncharacterized protein	-2.11	6.65E-10
llmg_0330	Putative permease protein	-2.09	2.23E-10
pnuC1	PnuC1 protein	-2.08	1.58E-07
fdhC	Putative formate dehydrogenase	-2.07	5.20E-06
llmg_1408	Putative uncharacterized protein	-2.05	3.25E-11
llmg_1409	Putative uncharacterized protein	-2.03	4.39E-10
llmg_1393	Putative uncharacterized protein	-2.02	3.70E-10
llmg_0329	ABC transporter ATP binding and permease protein	-2.02	9.49E-10
llmg_1387	Putative uncharacterized protein	-2.01	2.54E-09
llmg_1392	Putative uncharacterized protein	-2.01	5.05E-10
llmg_0856	Putative uncharacterized protein	-1.98	6.23E-08
llmg_0870	Transporter	-1.97	2.05E-08
llmg_1173	Putative uncharacterized protein	-1.94	4.30E-11
llmg_1390	Putative uncharacterized protein	-1.91	4.54E-08
llmg_1403	Putative uncharacterized protein	-1.90	3.57E-08
llmg_1389	Putative uncharacterized protein	-1.89	6.83E-10
llmg_1391	Putative uncharacterized protein	-1.88	1.25E-09
llmg_1410	Putative uncharacterized protein	-1.83	4.45E-07
traD	Conjugal transfer protein TraD	-1.83	5.75E-09
llmg_1395	Putative uncharacterized protein	-1.79	2.86E-10
lmrP	Multidrug resistance protein	-1.77	9.19E-09
pbuO	Xanthine/uracil/vitamin C permease	-1.76	1.10E-07
ltrD	LtrD protein	-1.75	6.83E-10
leuD	3-isopropylmalate dehydratase small subunit	-1.75	6.13E-09
dppA	Dipeptide-binding protein	-1.74	4.65E-08
llmg_2306	Putative uncharacterized protein	-1.74	9.49E-10
llmg_2445	Multidrug resistance protein	-1.68	2.16E-08
arcD2	Arginine/ornithine antiporter	-1.68	6.41E-10
llmg_1281	Putative ABC transporter ATP-binding protein	-1.67	8.66E-09

llmg_0050	Putative transposase helper protein for IS712A	-1.66	2.27E-05
llmg_1411	Putative uncharacterized protein	-1.66	1.02E-07
gltA	GltA protein	-1.66	3.33E-09
llmg_1283	Putative uncharacterized protein	-1.66	1.31E-08
llmg_1406	Putative uncharacterized protein	-1.65	3.59E-08
llmg_0147	Lambda repressor-like, DNA-binding	-1.65	1.46E-06
ptnD	PTS system, mannose-specific IID component	-1.65	4.53E-05
ilvD	Dihydroxy-acid dehydratase	-1.64	3.47E-09
llmg_1378	Putative uncharacterized protein	-1.63	7.12E-10
pmrB	Multidrug resistance efflux pump	-1.63	4.39E-04
llmg_0454	Putative beta-glucosides-specific IIBC component	-1.62	4.79E-08
llmg_0889	Putative membrane protein	-1.60	8.87E-06
cluA	Cell surface antigen I/II	-1.59	3.01E-09
ltrC	LtrC protein	-1.58	4.53E-08
llmg_1386	Putative uncharacterized protein	-1.55	5.05E-10
plpB	Lipoprotein	-1.55	1.16E-08
llmg_2011	Putative amino acid permease	-1.53	2.09E-06
llmg_0343	UPF0397 protein llmg_0343	-1.53	2.01E-06
llmg_0823	Putative transposase helper protein for IS712A	-1.52	4.82E-06
llmg_1029	Putative membrane protein	-1.52	3.59E-08
llmg_1474	Putative voltage gated chloride channel	-1.49	4.06E-06
llmg_2054	Putative uncharacterized protein	-1.49	1.01E-02
llmg_1382	Putative uncharacterized protein	-1.48	4.33E-07
llmg_0284	Putative uncharacterized protein	-1.47	9.99E-08
plpA	Lipoprotein	-1.47	2.14E-09
llmg_0375	Amino acid permease	-1.46	9.61E-07
leuC	3-isopropylmalate dehydratase large subunit	-1.45	2.30E-07
llmg_1377	Putative uncharacterized protein	-1.45	2.05E-08
llmg_1379	Predicted Zn peptidase	-1.45	3.88E-07
llmg_1261	Putative transposase helper protein for IS712A	-1.43	1.96E-06
llmg_2109	Putative transposase helper protein for IS712A	-1.42	8.07E-06
llmg_1384	Putative uncharacterized protein	-1.42	2.38E-08
llmg_1074	Putative transposase helper protein for IS712A	-1.42	3.60E-05
fadD	Long-chain acyl-CoA synthetase	-1.40	5.52E-08
llmg_1388	Putative uncharacterized protein	-1.37	6.69E-09
llmg_0578	Putative tRNA-processing ribonuclease BN	-1.37	1.99E-05
llmg_2340	Putative uncharacterized protein	-1.37	3.46E-06
ypbC	Putative membrane protein	-1.36	1.02E-05
llmg_1402	Putative uncharacterized protein	-1.35	7.45E-06
llmg_1385	Putative uncharacterized protein	-1.34	3.17E-08

llmg_1381	Putative uncharacterized protein	-1.33	2.12E-07
llmg_1612	Putative transposase helper protein for IS712A	-1.32	3.22E-05
llmg_0683	Putative transposase helper protein for IS712A	-1.32	1.28E-05
llmg_1583	Putative prenyltransferase	-1.32	2.99E-05
udp	Uridine phosphorylase	-1.30	7.67E-08
pepC	PepC protein	-1.29	1.80E-06
ctrA	Putative amino-acid transporter	-1.29	1.03E-06
llmg_1229	Putative uncharacterized protein	-1.29	1.44E-05
bacA	Undecaprenyl-diphosphatase	-1.29	1.73E-05
secY	Protein translocase subunit SecY	-1.28	2.34E-07
serS	Serine--tRNA ligase	-1.28	1.30E-06
gntP	Gluconate transport protein	-1.27	3.95E-07
zitP	Zinc ABC transporter permease protein	-1.26	3.45E-03
menA	Prenyltransferase, UbiA family	-1.26	1.81E-07
alaS	Alanine--tRNA ligase	-1.25	6.13E-09
noxB	NADH dehydrogenase	-1.24	3.98E-05
kinA	Sensor protein kinase kinA	-1.23	9.99E-08
ilvB	Acetolactate synthase	-1.22	1.44E-06
adhE	Alcohol-acetaldehyde dehydrogenase	-1.21	3.75E-06
llmg_1760	Queuosine precursor transporter QueT	-1.21	1.61E-08
ubiA	Putative prenyltransferase, UbiA family	-1.20	1.60E-06
llmg_1986	Putative uncharacterized protein	-1.20	3.83E-06
ilvA	IlvA protein	-1.18	4.33E-07
ilvN	Acetolactate synthase small subunit	-1.17	2.55E-07
llmg_1993	Hypothetical transporter	-1.16	5.52E-05
rpoC	DNA-directed RNA polymerase subunit beta'	-1.16	9.30E-04
dar	Acetoin(Diacetyl)reductase	-1.16	7.96E-07
llmg_1512	Putative ABC transporter ATP-binding protein	-1.14	1.28E-06
plpD	Lipoprotein	-1.14	6.53E-07
llmg_2291	Putative uncharacterized protein	-1.14	2.49E-06
rodA	Rod shape-determining protein RodA	-1.13	4.88E-06
hprT	HprT protein	-1.12	1.22E-05
pip	Phage infection protein	-1.11	4.34E-07
llmg_2290	Putative uncharacterized protein	-1.11	2.52E-06
llmg_0229	Putative uncharacterized protein	-1.11	2.86E-05
purA	Adenylosuccinate synthetase	-1.11	1.96E-06
plpC	Lipoprotein	-1.10	2.34E-07
llmg_2368	Putative uncharacterized protein	-1.10	2.88E-06
rplO	50S ribosomal protein L15	-1.10	4.65E-05
llmg_2184	Putative uncharacterized protein	-1.09	1.67E-06

llmg_1749	Putative membrane protein	-1.08	1.05E-06
thrS	Threonine--tRNA ligase	-1.08	3.62E-05
rfbX	Putative O-antigen transporter	-1.07	3.97E-06
ps342	Major head protein	-1.06	4.18E-06
llmg_2172	Putative nitroreductase	-1.05	2.76E-04
llmg_1101	Putative secreted protein	-1.05	8.63E-05
llmg_0443	Putative uncharacterized protein	-1.05	1.78E-03
llmg_0334	Thiamine transporter ThiT	-1.04	1.09E-06
pgiA	Glucose-6-phosphate isomerase	-1.04	4.56E-03
pepQ	Proline dipeptidase	-1.04	5.72E-04
llmg_1932	Sulfate/thiosulfate import ATP-binding protein cysA	-1.04	9.61E-07
matR	Group II intron-encoded protein LtrA	-1.04	7.25E-05
ldh	L-lactate dehydrogenase	-1.04	1.06E-04
llmg_1174	Putative uncharacterized protein	-1.03	9.12E-07
copA	Copper/potassium-transporting ATPase	-1.03	2.20E-04
femD	Phosphoglucosamine mutase	-1.03	9.88E-06
llmg_1016	Cationic transporter	-1.03	1.09E-06
llmg_0202	Predicted kinase related to dihydroxyacetone kinase	-1.02	7.26E-04
llmg_1740	HTH-type transcriptional regulator	-1.02	1.93E-03
llmg_2212	Putative uncharacterized protein	-1.01	2.72E-05
rpmGA	50S ribosomal protein L33 2	-1.00	1.84E-05
llmg_2278	Putative uncharacterized protein	-1.00	1.84E-05
llmg_1613	Putative secreted protein	-1.00	6.24E-04
ascB	6-phospho-beta-glucosidase	-1.00	6.19E-07
llmg_1990	Putative uncharacterized protein	-0.99	2.94E-04
asnB	Asparagine synthetase	-0.99	1.10E-03
ackA1	Acetate kinase	-0.98	2.40E-05
pgmB	Beta-phosphoglucomutase	-0.98	9.89E-06
llmg_0882	Putative membrane protein	-0.98	1.78E-06
llmg_0590	Putative membrane protein	-0.98	6.17E-05
llmg_1057	PsiE protein homolog	-0.97	1.96E-04
llmg_0007	GTP-dependent nucleic acid-binding protein	-0.97	4.22E-05
thiI	Probable tRNA sulfurtransferase	-0.97	1.11E-06
thiE	Thiamine-phosphate synthase	-0.97	1.33E-05
kinC	Sensor histidine kinase	-0.97	3.10E-05
hadL	Cryptic haloacid dehalogenase 1	-0.95	5.58E-05
llmg_0006	Putative HTH-type transcriptional regulator	-0.94	6.59E-04
gidA	tRNA uridine 5-carboxymethylaminomethyl modification enzyme MnmG	-0.94	4.57E-06
llmg_1644	Putative membrane protein	-0.94	3.00E-06
llmg_0228	Putative membrane protein	-0.93	4.23E-06

llmg_1330	Niacin transporter NiaX	-0.93	1.25E-05
kdgA	2-dehydro-3-deoxyphosphogluconate aldolase / 4-hydroxy-2-oxoglutarate aldolase	-0.93	6.17E-06
llmg_0328	Putative uncharacterized protein	-0.93	4.72E-04
llmg_0078	Putative uncharacterized protein	-0.92	8.26E-05
metE	5-methyltetrahydropteroyltriglutamate--homocysteine methyltransferase	-0.92	2.12E-06
llmg_0226	Possible surface protein	-0.92	6.67E-05
llmg_1028	Putative NAD(P)H nitroreductase	-0.92	1.44E-05
glcU	Putative glucose uptake protein glcU	-0.91	5.47E-06
ilvC	Ketol-acid reductoisomerase	-0.91	1.18E-06
pepF	PepF protein	-0.91	2.68E-05
aspC	Putative aminotransferase	-0.91	1.07E-06
llmg_0146	Aryl-alcohol dehydrogenase	-0.91	2.11E-04
llmg_2183	Putative uncharacterized protein	-0.91	9.04E-07
llmg_0168	Deoxyribonuclease	-0.90	8.71E-06
zitQ	Zinc ABC transporter ATP binding protein	-0.90	3.22E-02
llmg_1748	Putative membrane protein	-0.90	5.36E-05
llmg_1082	Putative membrane protein	-0.90	6.64E-05
rex	Redox-sensing transcriptional repressor rex	-0.88	3.24E-04
llmg_1251	Putative uncharacterized protein	-0.87	2.68E-03
metF	Methylenetetrahydrofolate reductase	-0.87	1.21E-06
llmg_0861	Putative uncharacterized protein	-0.87	6.86E-05
llmg_2369	Hypothetical secreted protein predicted by Glimmer/Critica	-0.87	2.01E-05
ps305	Putative uncharacterized protein ps305	-0.86	1.05E-04
llmg_1413	Putative membrane protein	-0.86	9.33E-04
epsR	Transcriptional regulator	-0.86	3.16E-05
ps109	Putative uncharacterized protein ps109	-0.85	3.01E-04
rluD	Pseudouridine synthase	-0.85	7.99E-04
llmg_1577	Putative membrane protein	-0.85	5.34E-06
llmg_0342	Amino acid ABC transporter permease protein	-0.85	1.50E-06
llmg_0141	Putative Transcriptional regulator	-0.84	2.32E-05
trmE	tRNA modification GTPase MnmE	-0.84	4.15E-05
llmg_1349	Putative uncharacterized protein	-0.83	8.81E-04
llmg_0916	Putative uncharacterized protein	-0.83	9.94E-04
purL	Phosphoribosylformylglycinamidine synthase 2	-0.83	7.82E-06
llmg_0310	Putative uncharacterized protein	-0.83	1.74E-03
llmg_0661	CorA like magnesium and cobalt transport protein	-0.82	1.00E-03
llmg_2213	Putative uncharacterized protein	-0.82	6.41E-05
metS	Methionine--tRNA ligase	-0.82	7.82E-06
llmg_2496	Putative uncharacterized protein	-0.82	2.50E-05
llmg_0315	Phosphonate ABC transporter permease protein	-0.81	3.84E-04

cstA	Carbon starvation protein A	-0.81	8.22E-05
llmg_0473	Putative uncharacterized protein	-0.81	2.99E-03
bmpA	Basic membrane protein A	-0.80	4.15E-04
llmg_1056	Cation transporter	-0.80	1.07E-03
purQ	Phosphoribosylformylglycinamidine synthase 1	-0.80	2.11E-05
llmg_0453	Similar to sucrose-specific PTS enzyme IIABC	-0.80	2.47E-05
llmg_2472	Putative uncharacterized protein	-0.80	5.97E-06
ps101	Phage integrase	-0.79	2.55E-06
guaC	GMP reductase	-0.79	2.28E-04
thrB	Homoserine kinase	-0.79	1.43E-03
llmg_1851	Putative membrane protein	-0.79	3.58E-06
hsdM	Putative type I site-specific deoxyribonuclease	-0.79	2.47E-05
cydB	Cytochrome d ubiquinol oxidase, subunit II	-0.79	1.11E-04
malF	Maltose transport system permease protein malF	-0.79	3.39E-05
llmg_0162	Putative uncharacterized protein	-0.79	1.36E-05
recQ	ATP-dependent DNA helicase RecQ	-0.79	7.30E-06
vacB2	Ribonuclease R	-0.78	1.41E-03
llmg_0985	Putative secreted protein	-0.78	8.38E-04
blt	Multidrug resistance protein	-0.78	2.55E-05
llmg_1066	Putative membrane protein	-0.78	1.59E-04
malE	Maltose ABC transporter substrate binding protein	-0.78	1.74E-04
dppD	Dipeptide transport ATP-binding protein dppD	-0.77	5.77E-04
llmg_0223	Putative galactofuranose transferase	-0.77	3.46E-06
llmg_0269	ABC transporter ATP-binding and permease protein	-0.77	3.51E-03
llmg_1957	ABC transporter, ATP-binding protein	-0.77	1.47E-04
llmg_0135	Hypothetical transmembrane protein	-0.77	6.38E-05
llmg_0585	Putative secreted protein	-0.77	2.98E-04
rpmD	50S ribosomal protein L30	-0.77	1.23E-02
purN	Phosphoribosylglycinamide formyltransferase	-0.77	7.27E-04
metE2	5-methyltetrahydropteroyltriglutamate--homocysteine methyltransferase	-0.77	1.96E-06
llmg_1426	Sucrose-specific PTS system IIBC component	-0.76	1.24E-03
llmg_1230	Putative uncharacterized protein	-0.76	1.55E-05
nupC	Purine/cytidine ABC transporter permease protein	-0.76	2.50E-03
pbp2B	Penicillin-binding protein 2B	-0.76	1.10E-03
ps347	Major tail protein	-0.76	1.45E-05
llmg_1203	ABC transporter ABC binding and permease protein	-0.75	1.63E-05
llmg_1245	Putative uncharacterized protein	-0.75	2.63E-03
llmg_1960	Putative uncharacterized protein	-0.75	2.52E-04
llmg_1086	Similar to cation (Calcium) transporting ATPase	-0.74	8.74E-04
llmg_0756	Putative secreted protein	-0.74	4.53E-05

trePP	Putative trehalose/maltose hydrolase	-0.74	1.19E-04
llmg_0227	Putative membrane protein	-0.73	3.29E-05
llmg_0065	Putative acetyltransferase, GNAT family	-0.73	1.17E-04
ps345	Putative uncharacterized protein ps345	-0.73	2.90E-04
gltD	Glutamate synthase, small subunit 1	-0.73	1.12E-04
typA	GTP-binding protein TypA/BipA homolog	-0.73	9.20E-05
snf	SWI/SNF family helicase	-0.73	3.16E-04
phoU	Phosphate transport system regulator phoU	-0.73	1.82E-04
rplL	50S ribosomal protein L7/L12	-0.73	8.49E-05
llmg_1736	Putative uncharacterized protein	-0.72	2.28E-03
acmA	Probable N-acetylmuramidase	-0.72	1.06E-04
llmg_0449	Putative uncharacterized protein	-0.71	3.24E-04
guaB	Inosine-5'-monophosphate dehydrogenase	-0.71	2.57E-03
dtd	D-tyrosyl-tRNA(Tyr) deacylase	-0.71	3.73E-04
tkt	Tkt protein	-0.71	1.03E-03
llmg_2452	Putative uncharacterized protein	-0.71	2.44E-04
llmg_1614	Putative uncharacterized protein	-0.71	4.56E-05
gmk	Gmk protein	-0.70	3.29E-05
tyrA	Prephenate dehydrogenase	-0.70	1.06E-03
aldR	Putative regulator AldR	-0.70	4.58E-03
llmg_0076	High confidence in function and specificity	-0.70	2.72E-04
llmg_0008	Putative HTH-type transcriptional regulator	-0.69	1.67E-05
ahpF	Alkyl hydroperoxide reductase subunit F	-0.69	1.89E-04
llmg_0887	Cation transport protein	-0.69	3.40E-04
llmg_0381	Putative membrane protein	-0.69	1.47E-03
llmg_1089	Carbamoyl-phosphate synthase large chain	-0.69	2.05E-02
deoD	Purine nucleoside phosphorylase DeoD-type	-0.69	1.51E-03
llmg_1446	Putative membrane protein	-0.69	1.27E-03
abiP	Abortive phage resistance protein abiP	-0.68	1.85E-04
recD	Exodeoxyribonuclease V alpha chain	-0.68	1.48E-05
pyrG	CTP synthase	-0.68	7.25E-03
glgD	Glucose-1-phosphate adenylyltransferase	-0.68	5.69E-03
llmg_0143	Membrane protein insertase YidC	-0.68	3.42E-04
llmg_0101	Putative membrane protein	-0.68	8.81E-04
llmg_0600	Glycosyl transferase	-0.68	3.33E-05
metB1	Cystathionine gamma-synthase	-0.68	1.59E-04
llmg_0263	Putative methyltransferase	-0.68	7.17E-04
llmg_1615	Putative acetyltransferase	-0.67	4.39E-04
ps333	Terminase small subunit	-0.67	1.84E-05
tyrS	Tyrosine--tRNA ligase	-0.67	8.41E-04

rheA	ATP-dependent RNA helicase	-0.67	1.26E-05
ps344	Putative uncharacterized protein ps344	-0.67	2.13E-05
llmg_1250	Putative Arsenical-resistance protein	-0.66	2.30E-04
ecsB	ABC-type transporter ATP-binding protein	-0.66	1.85E-04
chb	Chitin binding protein, putative	-0.66	4.99E-02
dppC	Dipeptide transport system permease protein dppC	-0.66	9.54E-05
pycA	Pyruvate carboxylase	-0.66	2.08E-04
llmg_1980	Putative uncharacterized protein	-0.66	1.49E-03
llmg_0130	Putative uncharacterized protein	-0.66	4.00E-03
rpoZ	DNA-directed RNA polymerase subunit omega	-0.66	4.25E-05
ps358	Putative uncharacterized protein ps358	-0.65	7.50E-03
llmg_0495	Putative uncharacterized protein	-0.65	1.21E-04
cbiQ	Putative cobalt ABC transporter permease protein	-0.65	5.77E-05
comC	Type 4 prepilin-like protein specific leader peptidase	-0.65	9.79E-03
cpdC	2',3'-cyclic-nucleotide 2'-phosphodiesterase	-0.65	1.39E-04
gapA	Glyceraldehyde 3-phosphate dehydrogenase	-0.65	4.78E-04
leuS	Leucine--tRNA ligase	-0.65	4.56E-05
thiM	Hydroxyethylthiazole kinase	-0.64	1.27E-03
llmg_2477	Lysine specific permease	-0.64	1.04E-02
gntK	Gluconate kinase	-0.63	7.14E-05
llmg_2238	Putative uncharacterized protein	-0.63	9.94E-04
llmg_1941	Putative uncharacterized protein	-0.63	4.54E-04
glpF3	Putative glycerol uptake facilitator protein	-0.63	6.40E-04
elaA	ElaA protein	-0.63	1.57E-02
oxlT	Oxalate/Formate Antiporter	-0.63	1.08E-02
rpmH	50S ribosomal protein L34	-0.62	1.99E-03
llmg_0394	Putative permease protein	-0.62	3.82E-04
cadA	Cation-transporting ATPase	-0.62	7.90E-04
llmg_0149	Putative uncharacterized protein	-0.62	8.02E-05
pepN	Aminopeptidase N	-0.61	1.98E-03
ps412	Hypothetical phage protein	-0.61	4.01E-04
llmg_0726	Putative uncharacterized protein	-0.61	2.93E-03
purM	Phosphoribosylformylglycinamidine cyclo-ligase	-0.61	2.16E-04
ps343	Putative uncharacterized protein ps343	-0.60	1.39E-03
glnP	Glutamine ABC transporter permease and substrate binding protein	-0.60	7.90E-04
glgC	Glucose-1-phosphate adenylyltransferase	-0.60	4.94E-03
llmg_1108	Putative membrane protein	-0.60	1.40E-02
rbsK	Ribokinase	-0.60	2.19E-03
recA	Protein RecA	-0.60	2.72E-04
purF	Amidophosphoribosyltransferase	-0.60	2.87E-02

ps504	Putative uncharacterized protein ps504	-0.59	1.70E-04
ilvE	Branched-chain-amino-acid aminotransferase	-0.59	8.71E-04
llmg_1305	Putative transcriptional regulator	-0.59	4.74E-03
hly	Hemolysin like protein	-0.59	3.15E-03
ugd	UDP-glucose 6-dehydrogenase	-0.59	4.01E-04
llmg_1911	Putative uncharacterized protein	-0.59	4.12E-03
llmg_0875	Cation-transporting ATPase	0.59	1.24E-04
ps429	Putative uncharacterized protein ps429	0.59	9.47E-03
chiC	Acidic endochitinase	0.59	1.27E-04
pflA	Pyruvate-formate lyase activating enzyme	0.59	1.47E-03
llmg_0294	Putative uncharacterized protein	0.59	4.94E-03
llmg_1762	Putative dehydrogenease / oxidoreductase	0.59	1.24E-04
copR	Transcriptional regulator	0.59	1.39E-03
llmg_1771	Putative rhodanese-related sulfurtransferase	0.60	1.95E-03
llmg_1165	Putative membrane protein	0.60	2.87E-04
llmg_2052	Putative uncharacterized protein	0.60	3.16E-03
rpoE	Probable DNA-directed RNA polymerase subunit delta	0.60	2.25E-03
llmg_1500	Putative methyltransferase	0.60	1.17E-04
llmg_2391	Putative membrane protein	0.60	5.34E-05
galK	Galactokinase	0.60	2.72E-04
ptcA	PTS system, cellobiose-specific IIA component	0.60	8.13E-05
trxB2	Ferredoxin--NADP reductase	0.60	3.86E-04
llmg_0420	Putative uncharacterized protein	0.60	1.97E-02
sbcD	Nuclease SbcCD subunit D	0.60	1.70E-04
llmg_1260	Putative uncharacterized protein	0.60	6.08E-04
ppa	Inorganic pyrophosphatase	0.61	8.81E-04
ps435	Putative uncharacterized protein ps435	0.61	5.04E-04
dfpB	Putative phosphopantothenate--cysteine ligase	0.61	2.76E-04
ruvA	Holliday junction ATP-dependent DNA helicase RuvA	0.61	2.52E-04
accB	Acetyl-CoA carboxylase biotin carboxyl carrier protein	0.61	3.00E-02
llmg_0688	Putative uncharacterized protein	0.62	1.46E-03
eraL	GTPase Era	0.62	9.33E-04
pepP	Aminopeptidase P	0.62	4.75E-04
llmg_1482	Putative uncharacterized protein	0.62	2.85E-04
llmg_1817	Putative beta-phosphoglucomutase	0.62	1.82E-04
llmg_1358	Putative uncharacterized protein	0.62	7.19E-04
llmg_1769	Putative uncharacterized protein	0.62	3.09E-04
llmg_1800	Putative secreted protein	0.62	1.26E-05
llmg_1659	Putative uncharacterized protein	0.62	4.53E-03
llmg_1190	Putative uncharacterized protein	0.62	3.54E-05

llmg_0713	Putative uncharacterized protein	0.62	2.71E-03
pppL	Putative phosphoprotein phosphatase	0.62	1.17E-04
pheT	Phenylalanyl-tRNA synthetase beta chain	0.63	2.19E-04
llmg_0645	Putative membrane protein	0.63	4.31E-03
llmg_2004	Putative uncharacterized protein	0.63	3.56E-04
llmg_1186	UPF0154 protein llmg_1186	0.63	2.48E-04
mesJ	tRNA(Ile)-lysidine synthase	0.63	2.26E-04
llrE	Two-component system regulator llrE	0.63	2.64E-05
llmg_1527	Putative transcription regulator	0.63	9.15E-04
llmg_1357	Putative uncharacterized protein	0.63	1.47E-03
llmg_1672	Putative uncharacterized protein	0.63	7.93E-05
llmg_1523	Putative uncharacterized protein	0.63	1.07E-04
llmg_0922	Putative secreted protein	0.63	2.61E-04
llmg_1710	Putative glycosyltransferase	0.64	3.13E-04
ftsL	Cell division protein	0.64	2.11E-04
recF	DNA replication and repair protein RecF	0.64	1.87E-03
ppiA	Peptidyl-prolyl cis-trans isomerase, cyclophilin-type	0.64	3.16E-05
rpsO	30S ribosomal protein S15	0.64	4.17E-04
llmg_0397	FMN-dependent NADH-azoreductase	0.64	6.09E-05
busR	Transcriptional repressor BusR, GntR family	0.64	1.20E-03
mapA	Maltose phosphorylase	0.64	4.54E-04
cspD2	Cold shock protein cspD	0.64	1.58E-02
llmg_1538	Putative isochorismatase	0.64	1.71E-04
llmg_2500	Putative uncharacterized protein	0.65	2.70E-04
llmg_1175	Ribosome biogenesis GTPase A	0.65	2.19E-04
kinE	Sensor kinase protein	0.65	3.76E-04
rpmF	50S ribosomal protein L32	0.65	1.05E-02
ps130	Putative uncharacterized protein ps130	0.65	7.25E-03
llmg_1448	Putative membrane protein	0.66	1.68E-04
dacA	DacA protein	0.66	1.04E-04
fruR	Transcriptional regulator of the fructose operon	0.66	5.00E-04
llmg_2187	Putative uncharacterized protein	0.66	3.21E-04
ps126	Putative DNA primase	0.66	2.24E-03
llmg_1365	Putative uncharacterized protein	0.66	7.33E-04
llmg_1650	Putative secreted protein	0.66	5.66E-05
llmg_0276	Oxidoreductase, aldo/keto reductase family	0.66	2.02E-04
oppB2	Peptide transport system permease protein oppB2	0.66	1.17E-03
llmg_0185	Acetyltransferase, GNAT family	0.66	1.13E-04
llrB	Two-component system regulator llrB	0.67	1.53E-03
llmg_0056	Putative uncharacterized protein	0.67	9.87E-04

llmg_1977	Putative MecA homolog	0.67	3.21E-04
acmD	N-acetylglucosaminidase	0.67	1.10E-03
llmg_2243	Putative uncharacterized protein	0.67	2.03E-05
llmg_0538	3-hydroxyacyl-[acyl-carrier-protein] dehydratase FabZ	0.67	2.69E-02
llmg_1197	Putative uncharacterized protein	0.68	3.29E-04
xylA	Xylose isomerase	0.68	2.22E-02
llmg_0884	Putative uncharacterized protein	0.68	2.02E-05
rpsU	30S ribosomal protein S21	0.68	2.29E-02
llmg_1598	Putative secreted protein	0.69	1.84E-04
rluA	Pseudouridine synthase	0.69	1.42E-02
llmg_0195	Putative NADH dehydrogenase	0.69	1.95E-03
llrF	Two-component system regulator llrF	0.69	1.21E-05
llmg_1353	Putative tellurite resistance protein	0.69	6.25E-04
lmrC	Multidrug resistance protein C	0.70	2.52E-04
llmg_0432	Similar to transcription regulator	0.70	2.11E-04
pcp	Pyrrolidone-carboxylate peptidase	0.70	8.78E-03
llmg_1269	Putative uncharacterized protein	0.70	1.02E-02
llmg_2435	Putative uncharacterized protein	0.70	1.63E-04
rmaD	Transcriptional regulator, MarR family	0.71	3.00E-02
llmg_1293	Putative uncharacterized protein	0.71	4.74E-02
ps413	Hypothetical phage protein	0.71	2.77E-02
llmg_1560	Stress induced DNA binding protein	0.71	1.41E-03
llmg_2248	Putative abortive phage resistance	0.71	2.94E-04
trmA	Regulatory protein spx 2	0.71	4.84E-05
ps310	Putative uncharacterized protein ps310	0.72	2.53E-03
llmg_2144	Putative uncharacterized protein	0.72	4.60E-05
llmg_1315	Putative RNA methyltransferase	0.72	4.35E-05
ps116	Phage repressor	0.72	5.11E-03
ps324	Putative uncharacterized protein ps324	0.72	1.27E-03
fhuR	HTH-type transcriptional regulator fhuR	0.72	1.01E-04
ps123	Putative uncharacterized protein ps123	0.73	6.15E-03
llmg_0708	Putative uncharacterized protein	0.73	1.18E-04
ps511	Putative uncharacterized protein ps511	0.74	2.03E-03
llmg_1596	UPF0145 protein llmg_1596	0.74	5.36E-05
llmg_1158	Putative uncharacterized protein	0.74	1.47E-03
llmg_1140	Putative uncharacterized protein	0.74	3.76E-04
pepDB	PepDB protein	0.74	2.81E-02
glpD	GlpD protein	0.75	1.88E-02
llmg_1148	Putative uncharacterized protein	0.75	9.07E-04
llmg_1019	Oxidoreductase, short-chain dehydrogenase/reductase family protein	0.75	2.42E-04

llmg_0905	Putative uncharacterized protein	0.75	1.72E-03
llmg_1597	Putative uncharacterized protein	0.76	1.45E-04
llmg_1857	Putative esterase	0.76	2.17E-05
ps338	Putative uncharacterized protein ps338	0.76	3.30E-03
llmg_0086	Putative membrane protein	0.76	2.08E-02
llmg_2531	Putative uncharacterized protein	0.76	1.10E-04
scpA	Segregation and condensation protein A	0.77	8.89E-05
llmg_2221	Putative membrane protein	0.77	6.34E-06
ps614	Putative uncharacterized protein ps614	0.77	1.63E-04
llmg_0424	Transcriptional regulator	0.77	1.67E-04
ps402	Putative uncharacterized protein ps402	0.77	9.47E-05
ps117	Putative uncharacterized protein ps117	0.77	5.75E-04
llmg_0670	Putative uncharacterized protein	0.77	5.93E-06
rmaE	Transcriptional regulator, MarR family	0.77	1.23E-02
rpsP	30S ribosomal protein S16	0.77	9.43E-03
llmg_1968	Putative uncharacterized protein	0.77	9.15E-03
llmg_0514	Regulatory protein spx	0.78	1.70E-04
drrA	Daunorubicin resistance ABC transporter ATP-binding subunit	0.78	5.98E-04
llmg_2010	UPF0213 protein llmg_2010	0.78	4.77E-02
llmg_2249	Putative uncharacterized protein	0.78	4.56E-03
llmg_0956	Transcriptional regulator, LacI family	0.78	6.65E-05
llmg_1085	Putative uncharacterized protein	0.79	7.22E-06
dnaK	Chaperone protein DnaK	0.79	2.53E-05
galU	UDP--glucose-1-phosphate uridylyltransferase	0.79	4.84E-05
ps325	Deoxyuridine 5'-triphosphate nucleotidohydrolase	0.79	5.43E-04
llmg_2482	Putative uncharacterized protein	0.79	7.14E-05
llmg_0159	Putative uncharacterized protein	0.80	3.39E-03
llmg_0553	Putative uncharacterized protein	0.80	6.34E-03
llmg_1772	Putative rhodanese-related sulfurtransferase	0.80	3.16E-04
llmg_2434	Putative uncharacterized protein	0.80	3.50E-04
hrcA	Heat-inducible transcription repressor HrcA	0.80	9.88E-05
llmg_2479	Putative uncharacterized protein	0.81	7.07E-05
llmg_2273	Putative uncharacterized protein	0.81	1.22E-04
luxS	S-ribosylhomocysteine lyase	0.81	2.82E-05
llmg_0866	Putative uncharacterized protein	0.81	9.71E-06
llmg_1708	Putative glycosyltransferase	0.81	7.01E-03
llmg_1258	Putative uncharacterized protein	0.81	6.37E-05
llmg_1188	Non-canonical purine NTP pyrophosphatase	0.81	3.45E-05
ps408	Putative cro repressor	0.82	9.61E-03
llmg_1434	Putative secreted protein	0.82	1.03E-04

llmg_0552	Putative glyoxylase	0.82	2.71E-03
llmg_0692	Putative uncharacterized protein	0.82	1.10E-03
llmg_1139	Putative uncharacterized protein	0.82	1.03E-03
xpt	Xanthine phosphoribosyltransferase	0.82	6.53E-06
pacA	Penicillin acylase	0.82	4.88E-06
llrD	RrD	0.83	1.05E-06
llmg_0969	Putative uncharacterized protein	0.83	4.36E-05
llmg_0687	Putative uncharacterized protein	0.83	1.25E-06
llmg_2337	Putative uncharacterized protein	0.83	5.60E-05
llmg_1999	Putative uncharacterized protein	0.83	7.58E-05
llmg_1751	Alkaline phosphatase superfamily protein	0.84	4.11E-05
llmg_0865	Transcriptional antiterminator, BglG family	0.84	2.40E-05
llmg_2146	Putative uncharacterized protein	0.84	3.81E-04
llmg_0710	Putative membrane protein	0.84	5.34E-06
ps612	Putative uncharacterized protein ps612	0.84	3.68E-05
llmg_1369	Putative uncharacterized protein	0.84	5.97E-06
llmg_0300	Putative uncharacterized protein	0.84	4.65E-04
llmg_1585	Putative amidase	0.84	1.17E-05
panE	2-dehydropantoate 2-reductase	0.85	4.91E-05
hisB	Imidazoleglycerol-phosphate dehydratase	0.85	4.30E-04
llmg_1362	Putative uncharacterized protein	0.85	1.68E-05
smpB	SsrA-binding protein	0.85	3.99E-05
llmg_1453	Oxidoreductase	0.85	2.17E-05
llmg_1236	Putative uncharacterized protein	0.86	1.56E-04
murD	UDP-N-acetylmuramoylalanine--D-glutamate ligase	0.86	2.10E-06
ps509	Hypothetical phage protein predicted by Glimmer/Critica	0.86	9.00E-06
llmg_1974	Probable ATP-dependent transporter	0.86	3.36E-06
als	Acetolactate synthase large subunit	0.86	3.09E-04
llmg_0281	Anaerobic ribonucleoside-triphosphate reductase	0.87	1.82E-04
llmg_0992	Putative nucleoside-diphosphate-sugar epimerases	0.87	2.38E-04
ps404	CI-like repressor	0.87	3.26E-03
llmg_0968	Oxidoreductase	0.88	6.89E-04
llmg_1859	Putative flavodoxin	0.88	5.36E-05
rmaB	Transcriptional regulator, MarR family	0.88	2.02E-03
llmg_2034	Hydrolase, haloacid dehalogenase-like family	0.88	3.83E-05
llmg_0970	Carboxymuconolactone decarboxylase family protein	0.88	1.45E-05
llmg_1166	Putative endoglucanase	0.88	1.12E-03
llmg_0686	Putative uncharacterized protein	0.88	9.61E-07
llmg_0274	Putative uncharacterized protein	0.88	3.16E-04
llmg_1322	Putative uncharacterized protein	0.89	6.88E-06

ps312	Putative uncharacterized protein ps312	0.89	2.53E-03
ocd	Ornithine cyclodeaminase, mu-crystallin homolog	0.89	2.46E-04
llmg_1155	Glutaminase	0.89	1.52E-03
llmg_1915	Putative Fe-S oxidoreductase	0.89	3.41E-06
ps519	Putative Terminase small subunit	0.89	2.34E-04
llmg_1160	Putative uncharacterized protein	0.90	3.03E-07
ps122	Putative uncharacterized protein ps122	0.90	3.89E-04
llmg_1115	XpaC-like protein	0.90	2.68E-05
aldC	AldC protein	0.90	3.15E-03
llmg_0167	Putative uncharacterized protein	0.90	3.60E-05
llmg_1052	Putative uncharacterized protein	0.91	1.09E-03
llmg_1513	Putative uncharacterized protein	0.91	7.87E-04
llmg_2247	Putative uncharacterized protein	0.91	1.58E-05
llmg_1533	Magnesium and cobalt transport protein	0.91	4.53E-05
llmg_1774	Putative uncharacterized protein	0.91	4.26E-02
llmg_2540	Putative uncharacterized protein	0.91	3.35E-04
tnp982	Transposase for insertion sequence element IS982B	0.91	1.99E-04
llmg_1802	Putative uncharacterized protein	0.92	1.41E-04
llmg_1347	Putative uncharacterized protein	0.92	2.05E-06
llmg_1416	Putative uncharacterized protein	0.92	2.05E-05
ps128	Putative uncharacterized protein ps128	0.92	6.14E-06
adaA	Methylphosphotriester-DNA alkyltransferase	0.92	3.13E-03
llmg_0128	Nucleoid-associated protein llmg_0128	0.92	6.87E-05
ps510	Putative uncharacterized protein ps510	0.93	9.83E-04
llmg_0422	Putative uncharacterized protein	0.93	1.26E-04
llmg_1324	Transcriptional regulator, araC family	0.93	3.94E-04
xseA	Exodeoxyribonuclease 7 large subunit	0.93	9.71E-06
llmg_0270	Putative uncharacterized protein	0.94	6.07E-06
bglA2	6-phospho-beta-glucosidase	0.94	1.09E-06
tnp1216	Transposase for insertion sequence-like element IS1216	0.94	4.77E-04
llmg_0716	Putative uncharacterized protein	0.95	1.93E-03
llmg_0696	Putative membrane protein	0.95	4.56E-03
llmg_1253	Putative uncharacterized protein	0.95	5.15E-05
llmg_1498	Putative uncharacterized protein	0.95	4.15E-06
nucA	5'-nucleotidase	0.95	1.62E-06
llmg_0883	Putative uncharacterized protein	0.95	6.38E-05
llmg_0573	Putative uncharacterized protein	0.96	2.58E-03
llmg_1550	UPF0374 protein llmg_1550	0.96	3.17E-05
llmg_1314	Putative uncharacterized protein	0.96	2.01E-06
llmg_2492	Putative uncharacterized protein	0.96	3.14E-05

tnp946	Transposase for insertion sequence element IS946	0.96	4.82E-06
llmg_1677	Putative secreted protein	0.96	1.28E-05
nrdI	Protein NrdI	0.97	9.40E-06
dnaG	DNA primase	0.97	9.44E-06
dpsA	Non-heme iron-binding ferritin	0.97	4.80E-05
hemH	Ferrochelatase	0.98	9.61E-07
llmg_0352	Oxidoreductase, aldo/keto reductase family	0.98	4.37E-05
llmg_1652	Putative uncharacterized protein	0.98	9.71E-06
ps119	Putative uncharacterized protein ps119	0.99	3.21E-03
llmg_1773	Putative uncharacterized protein	0.99	1.79E-03
llmg_2483	Putative uncharacterized protein	0.99	9.15E-04
llmg_1462	Putative HTH-type transcriptional regulator	0.99	5.81E-06
qor	Quinone oxidoreductase	0.99	6.79E-06
dut	Deoxyuridine 5'-triphosphate nucleotidohydrolase	0.99	1.38E-05
llmg_0962	Transcriptional regulator, araC family	0.99	8.76E-07
llmg_2336	Putative acetyltransferase	1.00	1.24E-04
llmg_1903	Putative transcriptional regulator	1.00	2.88E-06
llmg_1660	Transcriptional regulator, TetR family	1.00	5.48E-05
llmg_1766	Putative acetyltransferase	1.00	2.88E-06
rpmE	50S ribosomal protein L31 type B	1.00	4.84E-04
llmg_1676	ABC transporter permease protein	1.01	5.86E-06
acpD	FMN-dependent NADH-azoreductase	1.01	5.07E-06
nadD2	Probable nicotinate-nucleotide adenylyltransferase	1.01	9.60E-05
llmg_0904	Immunogenic secreted protein homolog	1.02	3.52E-05
xseB	Exodeoxyribonuclease 7 small subunit	1.02	3.42E-04
nrdE	Ribonucleoside-diphosphate reductase	1.02	4.88E-06
llmg_2143	Putative 20-kDa protein	1.02	8.09E-07
yphI	Putative uncharacterized protein yphI	1.02	1.58E-07
llmg_1257	Putative uncharacterized protein	1.02	8.73E-06
llmg_1461	Putative Di/Tri-peptide binding protein	1.03	5.02E-04
llmg_1526	Putative uncharacterized protein	1.03	1.38E-04
llmg_0275	Putative uncharacterized protein	1.03	4.19E-05
oppC2	Oligopeptide transport system permease protein oppC2	1.03	1.91E-05
llmg_0903	Putative serine/threonine phosphatase	1.03	1.85E-05
ps437	Putative uncharacterized protein ps437	1.04	1.04E-05
rpsT	30S ribosomal protein S20	1.04	1.79E-05
llmg_2051	Putative uncharacterized protein	1.04	1.24E-04
ps517	Putative Dna Primase	1.05	1.50E-06
llmg_0899	Putative uncharacterized protein	1.05	4.82E-07
hllA	HU-like DNA-binding protein	1.06	2.63E-04

grpE	Protein GrpE	1.06	1.01E-06
llmg_1205	Putative uncharacterized protein	1.06	5.93E-05
llmg_0572	Putative transcriptional regulator, MerR family	1.06	6.61E-06
ps410	Hypothetical phage protein	1.07	8.83E-04
llmg_2145	Putative uncharacterized protein	1.07	3.67E-06
llmg_0972	Putative uncharacterized protein	1.07	2.69E-06
llmg_1803	Putative secreted protein	1.07	1.46E-05
llmg_2032	Putative uncharacterized protein	1.07	1.24E-04
llmg_2223	Putative uncharacterized protein	1.08	7.60E-07
llmg_1432	Putative secreted protein	1.08	1.28E-05
llmg_2438	HTH-type transcriptional regulator	1.08	1.14E-05
ps124	Putative uncharacterized protein ps124	1.08	1.04E-05
ps514	Hypothetical phage protein predicted by Glimmer/Critica	1.08	1.60E-06
osmC	Osmotically inducible protein C	1.09	1.38E-05
glmS	Glutamine--fructose-6-phosphate aminotransferase [isomerizing]	1.09	4.61E-06
ps512	Putative uncharacterized protein ps512	1.10	1.56E-06
uspA	Universal stress protein A	1.10	9.61E-07
llmg_0252	Putative uncharacterized protein	1.10	1.09E-05
llmg_0603	HTH-type transcriptional regulator	1.10	9.12E-07
trxA	Thioredoxin	1.11	3.52E-05
ps610	Putative uncharacterized protein ps610	1.11	4.49E-07
llmg_0400	Putative uncharacterized protein	1.11	1.28E-05
llmg_1804	Transcriptional regulator, rgg family	1.11	7.55E-06
llmg_2498	Putative uncharacterized protein	1.11	5.48E-06
llmg_0380	Putative uncharacterized protein	1.11	4.35E-05
ps121	Putative DNA binding protein	1.12	1.47E-04
llmg_1159	Putative uncharacterized protein	1.12	2.51E-04
nrdH	Glutaredoxin-like protein NrdH	1.12	1.83E-04
llmg_1359	Putative uncharacterized protein	1.13	6.69E-06
llmg_0015	Putative RNA binding protein	1.13	3.30E-05
ps125	Phage dna replication	1.13	1.26E-04
arcC2	Carbamate kinase	1.14	7.30E-06
llmg_0477	Putative membrane protein	1.14	4.95E-06
clpC	ATP-dependent Clp protease ATP-binding subunit	1.14	1.49E-07
llmg_2016	Putative uncharacterized protein	1.15	1.77E-05
llmg_0479	Putative uncharacterized protein	1.15	3.23E-06
acmC	N-acetylglucosaminidase	1.15	5.93E-07
mraW	Ribosomal RNA small subunit methyltransferase H	1.16	2.75E-07
llmg_1360	Putative uncharacterized protein	1.16	2.88E-06
radC	UPF0758 protein llmg_1515	1.17	6.66E-07

rpmB	50S ribosomal protein L28	1.18	4.24E-05
ps513	Putative uncharacterized protein ps513	1.19	1.76E-06
llmg_2436	Putative uncharacterized protein	1.19	6.47E-06
ps603	Putative uncharacterized protein ps603	1.19	2.01E-06
gpo	Glutathione peroxidase	1.20	1.12E-06
llmg_1707	Putative glycosyltransferase	1.21	8.24E-06
tetR	Similar to transcriptional regulator	1.21	1.05E-05
llmg_1317	Putative N-acetylmannosamine-6-phosphate 2-epimerase	1.21	5.05E-06
hdiR	HTH-type transcriptional regulator HdiR	1.22	3.28E-06
llmg_0760	Putative transglycosylase	1.22	2.44E-06
llmg_0189	Putative uncharacterized protein	1.22	9.94E-06
llmg_0188	Putative uncharacterized protein	1.22	2.46E-06
llmg_1460	Putative Di/Tri-peptide transport ATP-binding protein	1.22	9.25E-07
llmg_0898	Putative uncharacterized protein	1.23	6.38E-05
llmg_0317	Putative uncharacterized protein	1.23	1.73E-05
llmg_1079	Putative uncharacterized protein	1.23	5.89E-05
ps606	Putative uncharacterized protein ps606	1.23	1.16E-07
llmg_1182	Putative acetyltransferase	1.24	8.66E-05
ps518	Hypothetical phage protein predicted by Glimmer/Critica	1.24	7.14E-07
llmg_2338	Putative uncharacterized protein	1.24	3.67E-06
llmg_1050	Putative secreted protein	1.25	1.06E-03
gadR	Positive regulator GadR	1.26	2.81E-07
llmg_1768	Putative uncharacterized protein	1.26	2.49E-06
ps515	Hypothetical phage protein predicted by Glimmer/Critica	1.27	2.00E-05
nrdF	Ribonucleoside-diphosphate reductase subunit beta	1.27	2.20E-07
llmg_1348	Putative uncharacterized protein	1.27	1.14E-05
llmg_1632	Putative hydrolase	1.27	3.46E-06
llmg_1806	Putative uncharacterized protein	1.28	1.64E-07
ps609	Putative uncharacterized protein ps609	1.28	1.63E-04
rmaA	Transcriptional regulator, MarR family	1.28	2.88E-06
llmg_0089	Putative HTH-type transcriptional regulator	1.29	3.34E-07
llmg_1454	Putative sugar kinase	1.29	4.67E-06
ps611	Putative uncharacterized protein ps611	1.29	9.09E-03
llmg_1629	Putative methyltransferase	1.29	1.13E-04
comX	Competence regulator ComX	1.30	1.28E-05
ps311	Putative uncharacterized protein ps311	1.30	3.30E-05
fhuC	Ferrichrome ABC transporter fhuC	1.30	9.41E-08
llmg_2481	Putative chorismate mutase	1.31	1.83E-04
ps516	Hypothetical phage protein predicted by Glimmer/Critica	1.31	3.16E-05
llmg_1495	Putative uncharacterized protein	1.32	8.73E-06

llmg_2333	Putative uncharacterized protein	1.34	4.41E-04
oppA2	Oligopeptide-binding protein oppA2	1.34	9.36E-05
ps357	Putative uncharacterized protein ps357	1.34	3.18E-05
ps431	Putative uncharacterized protein ps431	1.35	6.57E-05
llmg_0685	Putative uncharacterized protein	1.35	2.00E-06
clpB	Chaperone protein ClpB	1.35	1.67E-06
ps608	Putative uncharacterized protein ps608	1.37	4.49E-08
arsC	Arsenate reductase	1.40	2.18E-04
llmg_1529	6,7-dimethyl-8-ribityllumazine synthase	1.40	5.89E-05
llmg_2339	Transcriptional regulator	1.41	1.80E-05
llmg_1965	Putative AMP-binding enzyme	1.41	6.63E-08
llmg_0959	Beta-glucosidase	1.42	3.17E-08
rmaI	Transcriptional regulator, MarR family	1.42	1.73E-06
llmg_0248	Transcriptional regulator, TetR family	1.43	4.22E-07
llmg_1675	ABC transporter ATP-binding protein	1.44	2.55E-06
ps508	Hypothetical phage protein predicted by Glimmer/Critica	1.44	1.49E-06
padR	Putative transcriptional repressor of padC	1.44	3.24E-06
comFC	Putative late competence protein	1.45	2.67E-04
ntd	Nucleoside deoxyribosyltransferase	1.45	1.15E-07
ps605	Putative uncharacterized protein ps605	1.46	8.61E-08
fur	Ferric uptake regulation protein	1.48	7.96E-07
llmg_0960	Beta-glucosidase	1.48	1.17E-09
llmg_1458	Putative Di/Tri-peptide transport system permease protein	1.49	1.44E-06
llmg_1703	Putative uncharacterized protein	1.50	2.01E-05
llmg_1554	Transcriptional regulator, TetR family	1.50	1.69E-07
llmg_1904	Putative nitroreductase	1.52	2.81E-06
llmg_1553	Putative ABC transporter ATP-binding protein	1.53	2.04E-09
llmg_1252	Putative uncharacterized protein	1.54	5.23E-06
ps604	Putative uncharacterized protein ps604	1.54	6.83E-10
clpE	ATP-dependent Clp protease ATP-binding subunit ClpE	1.54	5.13E-06
cspB	Cold shock-like protein cspB	1.57	2.61E-04
llmg_0458	Glucosyltransferase-I	1.57	1.23E-07
bglX	Beta-glucosidase	1.58	2.29E-08
ctsR	Transcriptional regulator ctsR	1.59	1.04E-08
nagB	Glucosamine-6-phosphate deaminase	1.59	1.96E-06
uspA2	Universal stress protein A2	1.59	2.33E-07
llmg_1920	Putative uncharacterized protein	1.60	1.41E-05
clpP	ATP-dependent Clp protease proteolytic subunit	1.60	2.38E-08
llmg_0564	Putative soluble lytic murein transglycosylase	1.61	1.00E-06
llmg_0709	Transcriptional regulator, PadR-like family	1.61	5.12E-07

arcA	Arginine deiminase	1.62	2.38E-08
llmg_1658	Putative uncharacterized protein	1.63	1.31E-06
dltE	Oxidoreductase dltE	1.64	2.38E-08
feoA	Ferrous iron transport protein A	1.65	3.85E-09
dukA	Deoxyguanosine kinase	1.68	1.81E-06
llmg_0155	Putative uncharacterized protein	1.70	6.13E-09
llmg_1344	Putative uncharacterized protein	1.79	2.75E-09
llmg_1663	Putative uncharacterized protein	1.82	2.66E-06
llmg_1130	Putative uncharacterized protein	1.84	1.40E-06
cbr	Carbonyl reductase cbr	1.84	3.28E-09
llmg_0991	Transcriptional regulator, AcrR family	1.86	2.99E-08
llmg_1761	Putative uncharacterized protein	1.88	9.61E-07
ps340	Putative uncharacterized protein ps340	1.88	9.44E-06
llmg_2426	Putative uncharacterized protein	1.89	1.91E-04
llmg_0353	Putative transcription regulator	1.89	2.49E-08
rpe2	Ribulose-phosphate 3-epimerase	1.91	3.70E-10
rpiB	Ribose 5-phosphate isomerase B	1.91	1.26E-08
ps407	Putative uncharacterized protein ps407	1.96	1.67E-06
arcD1	Arginine/ornithine antiporter	1.97	3.17E-08
arcC1	Carbamate kinase	1.97	5.05E-10
llmg_1457	Putative Di/Tri-peptide transport ATP-binding protein	2.00	1.17E-07
llmg_0245	Putative zinc-binding dehydrogenase	2.05	2.75E-07
llmg_0354	Putative glyoxylase	2.08	2.86E-10
ribD	Riboflavin biosynthesis protein RibD	2.09	1.95E-08
llmg_0527	Putative uncharacterized protein	2.10	6.41E-10
rmeC	Transcriptional regulator, MerR family	2.21	5.05E-10
llmg_0485	Putative membrane protein	2.27	2.86E-10
arcB	Ornithine carbamoyltransferase, catabolic	2.35	2.21E-09
groEL2	60 kDa chaperonin	2.36	2.90E-10
groES	10 kDa chaperonin	2.41	7.12E-10
llmg_1235	Cyclic nucleotide-binding domain protein	2.42	1.95E-08
rmeB	Transcriptional regulator, MerR family	2.49	2.49E-08
ribB	RibB protein	2.53	2.84E-07
ribA	Riboflavin biosynthesis protein ribA	2.70	3.03E-07
llmg_0246	Putative uncharacterized protein	2.86	4.38E-12
llmg_0490	Sugar transport system permease protein	2.96	4.30E-11
llmg_0489	Sugar transport system permease protein	3.05	5.55E-13
llmg_0247	Putative UDP-glucose 4-epimerase	3.25	2.03E-12
llmg_0486	Putative membrane protein	3.27	2.03E-14
llmg_0488	Multiple sugar-binding protein	3.89	2.71E-14

llmg_0487	Putative trehalose/maltose hydrolase	4.10	1.27E-03

Table S2. Differentially expressed genes in TM29 (38°C vs. 30°C)

Gene name	Protein name	logFC	adj.P.Val
llmg_1396	Putative uncharacterized protein	-4.10	1.08E-15
llmg_1397	Putative uncharacterized protein	-3.97	4.13E-15
llmg_1392	Putative uncharacterized protein	-3.91	9.44E-15
llmg_1393	Putative uncharacterized protein	-3.74	1.09E-14
llmg_1395	Putative uncharacterized protein	-3.70	2.31E-15
llmg_1387	Putative uncharacterized protein	-3.67	9.61E-14
llmg_1386	Putative uncharacterized protein	-3.63	1.08E-15
llmg_1389	Putative uncharacterized protein	-3.51	2.39E-14
llmg_1390	Putative uncharacterized protein	-3.45	3.82E-12
llmg_1391	Putative uncharacterized protein	-3.42	5.37E-14
traD	Conjugal transfer protein TraD	-3.35	2.21E-13
llmg_1388	Putative uncharacterized protein	-3.35	9.33E-15
llmg_1384	Putative uncharacterized protein	-3.34	4.44E-14
cluA	Cell surface antigen I/II	-3.29	2.72E-14
llmg_1402	Putative uncharacterized protein	-3.15	5.24E-11
llmg_1385	Putative uncharacterized protein	-3.12	5.96E-14
llmg_1407	Putative uncharacterized protein	-3.08	4.90E-13
ps344	Putative uncharacterized protein ps344	-3.00	2.39E-14
llmg_1377	Putative uncharacterized protein	-2.97	2.08E-13
llmg_1382	Putative uncharacterized protein	-2.96	1.00E-11
llmg_1409	Putative uncharacterized protein	-2.91	3.09E-13
ltrC	LtrC protein	-2.91	2.85E-12
ltrD	LtrD protein	-2.90	9.40E-14
ps341	Putative uncharacterized protein ps341	-2.81	1.12E-09
llmg_1104	Drug-export protein	-2.79	1.10E-11
ps345	Putative uncharacterized protein ps345	-2.79	8.54E-12
ps343	Putative uncharacterized protein ps343	-2.78	8.54E-12
llmg_1379	Predicted Zn peptidase	-2.76	1.80E-11
ps347	Major tail protein	-2.75	2.08E-13
llmg_1408	Putative uncharacterized protein	-2.75	4.37E-14
llmg_1378	Putative uncharacterized protein	-2.72	9.40E-14
llmg_2054	Putative uncharacterized protein	-2.65	5.37E-05
llmg_1406	Putative uncharacterized protein	-2.62	1.72E-11
ps342	Major head protein	-2.62	9.89E-12
llmg_1381	Putative uncharacterized protein	-2.60	6.59E-12
llmg_1411	Putative uncharacterized protein	-2.60	6.87E-11
ps348	Putative uncharacterized protein ps348	-2.57	3.08E-10
ps354	Putative uncharacterized protein ps354	-2.57	4.39E-12
llmg_1410	Putative uncharacterized protein	-2.50	2.43E-09
ps355	Putative holin	-2.44	1.75E-09
matR	Group II intron-encoded protein LtrA	-2.42	1.12E-09
llmg_1747	Amino acid permease	-2.42	4.94E-09
amtB	Ammonium transporter AmtB	-2.40	2.83E-08

ps351	Putative uncharacterized protein ps351	-2.32	4.42E-11
ps346	Putative uncharacterized protein ps346	-2.29	2.16E-10
ps350	Putative uncharacterized protein ps350	-2.26	9.28E-10
ps349	Putative uncharacterized protein ps349	-2.25	1.33E-09
leuD	3-isopropylmalate dehydratase small subunit	-2.13	9.97E-11
glnB	Nitrogen regulatory protein P-II	-2.10	4.00E-09
llmg_1281	Putative ABC transporter ATP-binding protein	-2.09	9.97E-11
citB	Aconitate hydratase	-1.94	9.79E-10
ps356	Endolysin	-1.92	2.83E-08
ps353	Putative uncharacterized protein ps353	-1.88	3.67E-08
pbuO	Xanthine/uracil/vitamin C permease	-1.82	2.26E-08
ps352	Putative uncharacterized protein ps352	-1.82	3.17E-09
leuC	3-isopropylmalate dehydratase large subunit	-1.80	4.00E-09
llmg_1283	Putative uncharacterized protein	-1.80	1.12E-09
ilvN	Acetolactate synthase small subunit	-1.73	4.49E-10
ps334	Terminase large subunit	-1.69	1.65E-09
llmg_0050	Putative transposase helper protein for IS712A	-1.66	1.18E-05
ilvD	Dihydroxy-acid dehydratase	-1.65	7.82E-10
ps335	Portal protein	-1.63	6.54E-07
llmg_1016	Cationic transporter	-1.62	9.79E-10
ilvB	Acetolactate synthase	-1.61	1.54E-08
fdhC	Putative formate dehydrogenase	-1.58	4.43E-05
icd	Isocitrate dehydrogenase [NADP]	-1.57	4.13E-07
llmg_1403	Putative uncharacterized protein	-1.56	1.42E-07
llmg_0823	Putative transposase helper protein for IS712A	-1.55	1.68E-06
gltA	GltA protein	-1.54	2.16E-09
hprT	HprT protein	-1.53	1.47E-07
llmg_0147	Lambda repressor-like, DNA-binding	-1.50	1.84E-06
purH	Bifunctional purine biosynthesis protein PurH	-1.49	1.84E-09
llmg_1229	Putative uncharacterized protein	-1.49	1.38E-06
ypbC	Putative membrane protein	-1.46	2.17E-06
llmg_1173	Putative uncharacterized protein	-1.45	2.54E-10
llmg_0443	Putative uncharacterized protein	-1.45	5.62E-05
llmg_1373	Putative uncharacterized protein	-1.43	6.17E-05
lmrP	Multidrug resistance protein	-1.43	4.03E-08
llmg_0683	Putative transposase helper protein for IS712A	-1.40	3.13E-06
llmg_0856	Putative uncharacterized protein	-1.37	2.00E-06
llmg_2306	Putative uncharacterized protein	-1.34	7.03E-09
arcD2	Arginine/ornithine antiporter	-1.33	2.48E-09
ps309	Putative uncharacterized protein ps309	-1.33	1.84E-07
pnuC1	PnuC1 protein	-1.32	1.18E-05
ps336	Putative uncharacterized protein ps336	-1.31	1.34E-06
ps337	Putative uncharacterized protein ps337	-1.30	4.09E-07
llmg_1474	Putative voltage gated chloride channel	-1.30	8.77E-06
pnuC2	Nicotinamide mononucleotide transporter	-1.30	9.91E-04
llmg_1594	Gamma-glutamyl-diamino acid-endopeptidase	-1.28	1.51E-07

ps333	Terminase small subunit	-1.27	3.23E-09
llmg_2445	Multidrug resistance protein	-1.26	2.59E-07
llmg_1612	Putative transposase helper protein for IS712A	-1.25	3.18E-05
llmg_1074	Putative transposase helper protein for IS712A	-1.23	7.91E-05
ilvC	Ketol-acid reductoisomerase	-1.23	8.72E-09
ilvA	IlvA protein	-1.22	8.96E-08
llmg_1261	Putative transposase helper protein for IS712A	-1.22	4.93E-06
AM_new	Group II intron-interrupted relaxase LtrB	-1.21	1.47E-07
zitP	Zinc ABC transporter permease protein	-1.19	3.28E-03
ps306	Putative uncharacterized protein ps306	-1.14	1.81E-07
llmg_2202	Putative uncharacterized protein	-1.14	5.24E-08
llmg_2109	Putative transposase helper protein for IS712A	-1.14	4.36E-05
llmg_1174	Putative uncharacterized protein	-1.14	9.20E-08
llmg_1399	Putative uncharacterized protein	-1.12	1.49E-02
ps308	Putative uncharacterized protein ps308	-1.12	9.00E-05
ldh	L-lactate dehydrogenase	-1.12	2.58E-05
secG	Protein-export membrane protein secG	-1.11	3.78E-07
llmg_1583	Putative prenyltransferase	-1.10	9.40E-05
llmg_1760	Queuosine precursor transporter QueT	-1.09	1.67E-08
ps319	Putative uncharacterized protein ps319	-1.09	4.12E-03
ps320	Putative uncharacterized protein ps320	-1.09	1.40E-04
zitQ	Zinc ABC transporter ATP binding protein	-1.08	8.50E-03
llmg_1446	Putative membrane protein	-1.06	1.11E-05
aldR	Putative regulator AldR	-1.06	8.59E-05
ps313	Putative transcription regulator	-1.04	2.48E-06
fadD	Long-chain acyl-CoA synthetase	-1.04	7.88E-07
guaC	GMP reductase	-1.02	9.60E-06
ps310	Putative uncharacterized protein ps310	-1.02	6.66E-05
ps318	Putative uncharacterized protein ps318	-1.01	1.35E-06
llmg_1251	Putative uncharacterized protein	-0.99	5.86E-04
vacB2	Ribonuclease R	-0.99	1.08E-04
ps323	Putative uncharacterized protein ps323	-0.98	3.15E-04
llmg_1349	Putative uncharacterized protein	-0.98	1.21E-04
llmg_1993	Hypothetical transporter	-0.98	1.71E-04
llmg_1613	Putative secreted protein	-0.97	4.77E-04
ps427	Putative uncharacterized protein ps427	-0.97	3.07E-05
llmg_1268	Tyrosine recombinase	-0.96	7.33E-03
ps321	Putative uncharacterized protein ps321	-0.96	2.19E-06
llmg_1433	Putative secreted protein	-0.95	4.79E-05
llmg_1029	Putative membrane protein	-0.94	4.08E-06
purA	Adenylosuccinate synthetase	-0.93	5.88E-06
llmg_2011	Putative amino acid permease	-0.93	1.90E-04
llmg_0895	Putative uncharacterized protein	-0.93	1.17E-05
llmg_0284	Putative uncharacterized protein	-0.93	8.51E-06
llmg_0146	Aryl-alcohol dehydrogenase	-0.93	9.85E-05
ps339	Putative uncharacterized protein ps339	-0.92	3.33E-05

llmg_2212	Putative uncharacterized protein	-0.92	3.61E-05
aldB	Alpha-acetolactate decarboxylase	-0.92	6.85E-08
dtd	D-tyrosyl-tRNA(Tyr) deacylase	-0.90	2.10E-05
ltrE	LtrE protein	-0.90	1.34E-06
llmg_1932	Sulfate/thiosulfate import ATP-binding protein cysA	-0.90	2.05E-06
llmg_1986	Putative uncharacterized protein	-0.89	4.49E-05
metE	5-methyltetrahydropteroyltriglutamate--homocysteine methyltransferase	-0.89	1.38E-06
llmg_0006	Putative HTH-type transcriptional regulator	-0.88	7.35E-04
ilvE	Branched-chain-amino-acid aminotransferase	-0.87	1.06E-05
telC	Putative tellurium resistance protein	-0.87	2.53E-05
llmg_1269	Putative uncharacterized protein	-0.87	1.45E-03
llmg_2184	Putative uncharacterized protein	-0.86	1.01E-05
ps449	Putative uncharacterized protein ps449	-0.85	9.67E-03
thiI	Probable tRNA sulfurtransferase	-0.85	2.15E-06
llmg_0726	Putative uncharacterized protein	-0.85	1.06E-04
ps315	Putative uncharacterized protein ps315	-0.84	1.43E-04
llmg_1740	HTH-type transcriptional regulator	-0.84	4.90E-03
llmg_2183	Putative uncharacterized protein	-0.83	8.80E-07
ps316	Putative uncharacterized protein ps316	-0.83	1.11E-03
phoU	Phosphate transport system regulator phoU	-0.82	2.78E-05
purB	PurB protein	-0.82	4.93E-06
llmg_1230	Putative uncharacterized protein	-0.82	3.36E-06
llmg_0329	ABC transporter ATP binding and permease protein	-0.82	1.88E-05
llmg_1228	Putative uncharacterized protein	-0.81	1.44E-03
llmg_1305	Putative transcriptional regulator	-0.81	2.08E-04
llmg_0578	Putative tRNA-processing ribonuclease BN	-0.81	1.43E-03
pip	Phage infection protein	-0.81	6.81E-06
ps307	Putative uncharacterized protein ps307	-0.81	9.09E-04
udp	Uridine phosphorylase	-0.80	8.77E-06
llmg_1146	Putative uncharacterized protein	-0.80	1.57E-02
ps330	Putative uncharacterized protein ps330	-0.80	6.99E-05
llmg_1440	Putative uncharacterized protein	-0.79	9.67E-04
llmg_0168	Deoxyribonuclease	-0.79	1.81E-05
rodA	Rod shape-determining protein RodA	-0.79	1.02E-04
alaS	Alanine--tRNA ligase	-0.79	6.62E-07
pepQ	Proline dipeptidase	-0.79	3.11E-03
gidA	tRNA uridine 5-carboxymethylaminomethyl modification enzyme MnmG	-0.78	1.56E-05
cspE	Cold shock-like protein cspE	-0.78	1.12E-02
ctrA	Putative amino-acid transporter	-0.78	1.08E-04
aroE	Shikimate dehydrogenase	-0.78	6.03E-06
ps327	Putative uncharacterized protein ps327	-0.77	5.49E-05
ps322	Putative uncharacterized protein ps322	-0.77	3.74E-03
asnB	Asparagine synthetase	-0.77	4.51E-03
ps109	Putative uncharacterized protein ps109	-0.77	4.35E-04
metF	Methylenetetrahydrofolate reductase	-0.77	2.07E-06
llmg_1512	Putative ABC transporter ATP-binding protein	-0.76	4.55E-05

rluD	Pseudouridine synthase	-0.76	1.19E-03
hadL	Cryptic haloacid dehalogenase 1	-0.76	2.76E-04
llmg_0330	Putative permease protein	-0.76	1.12E-05
llmg_2278	Putative uncharacterized protein	-0.75	1.76E-04
llmg_1007	Putative uncharacterized protein	-0.75	6.85E-04
noxB	NADH dehydrogenase	-0.75	1.98E-03
metS	Methionine--tRNA ligase	-0.75	1.03E-05
llmg_0870	Transporter	-0.75	3.87E-04
llmg_1028	Putative NAD(P)H nitroreductase	-0.74	6.99E-05
metB1	Cystathionine gamma-synthase	-0.73	3.92E-05
llmg_2291	Putative uncharacterized protein	-0.73	1.40E-04
deoD	Purine nucleoside phosphorylase DeoD-type	-0.72	6.34E-04
pgiA	Glucose-6-phosphate isomerase	-0.72	2.87E-02
llmg_0375	Amino acid permease	-0.71	6.42E-04
ps428	Deoxyuridine 5'-triphosphate nucleotidohydrolase	-0.71	3.64E-04
llmg_1082	Putative membrane protein	-0.71	3.49E-04
rpoZ	DNA-directed RNA polymerase subunit omega	-0.70	1.14E-05
serS	Serine--tRNA ligase	-0.70	3.20E-04
thrS	Threonine--tRNA ligase	-0.70	1.13E-03
llmg_1400	Putative uncharacterized protein	-0.69	2.40E-04
gmk	Gmk protein	-0.69	2.16E-05
ps446	Putative major head protein	-0.69	3.80E-05
llmg_1540	Glycerol-3-phosphate acyltransferase	-0.69	1.85E-03
llmg_2368	Putative uncharacterized protein	-0.68	2.01E-04
ackA1	Acetate kinase	-0.68	4.59E-04
rplO	50S ribosomal protein L15	-0.67	2.10E-03
llmg_2543	Putative uncharacterized protein	-0.67	1.40E-03
abiP	Abortive phage resistance protein abiP	-0.67	1.25E-04
llmg_2213	Putative uncharacterized protein	-0.67	2.59E-04
secY	Protein translocase subunit SecY	-0.67	1.25E-04
ps317	Putative replisome organiser protein	-0.67	4.97E-04
plpB	Lipoprotein	-0.66	7.61E-05
aroH	Phospho-2-dehydro-3-deoxyheptonate aldolase	-0.66	1.21E-03
telB	Putative tellurium resistance protein	-0.66	1.90E-03
llmg_2290	Putative uncharacterized protein	-0.66	2.84E-04
llmg_0310	Putative uncharacterized protein	-0.65	6.22E-03
llmg_1355	Putative uncharacterized protein	-0.65	2.07E-03
purN	Phosphoribosylglycinamide formyltransferase	-0.65	1.65E-03
purM	Phosphoribosylformylglycinamidine cyclo-ligase	-0.65	5.93E-05
ps421	Putative uncharacterized protein ps421	-0.65	9.55E-05
kupB	Probable potassium transport system protein kup 2	-0.64	1.70E-04
llmg_1354	Putative uncharacterized protein	-0.64	1.43E-03
llmg_1356	Putative uncharacterized protein	-0.64	5.72E-04
ps326	Putative uncharacterized protein ps326	-0.64	3.16E-03
pepF	PepF protein	-0.63	4.97E-04
llmg_0391	Putative membrane protein	-0.63	2.80E-05

llmg_2369	Hypothetical secreted protein predicted by Glimmer/Critica	-0.62	3.04E-04
llmg_0916	Putative uncharacterized protein	-0.62	5.65E-03
ps101	Phage integrase	-0.62	1.83E-05
ps312	Putative uncharacterized protein ps312	-0.62	1.73E-02
gapA	Glyceraldehyde 3-phosphate dehydrogenase	-0.62	4.32E-04
bmpA	Basic membrane protein A	-0.62	2.18E-03
guaB	Inosine-5'-monophosphate dehydrogenase	-0.61	4.88E-03
epsR	Transcriptional regulator	-0.61	4.54E-04
llmg_0343	UPF0397 protein llmg_0343	-0.61	5.41E-03
llmg_1848	Putative uncharacterized protein	-0.60	1.25E-04
elaA	ElaA protein	-0.60	1.33E-02
kinA	Sensor protein kinase kinA	-0.60	1.27E-04
plpA	Lipoprotein	-0.60	3.33E-05
dppD	Dipeptide transport ATP-binding protein dppD	-0.60	2.72E-03
glpT	Glycerol-3-phosphate transporter	-0.60	1.78E-02
llmg_1841	Putative uncharacterized protein	-0.59	3.20E-04
llmg_2496	Putative uncharacterized protein	-0.59	3.04E-04
llmg_2244	Putative uncharacterized protein	-0.59	1.08E-03
typA	GTP-binding protein TypA/BipA homolog	-0.59	3.71E-04
llmg_1843	Putative uncharacterized protein	-0.59	7.74E-04
ps435	Putative uncharacterized protein ps435	0.59	4.07E-04
fhuG	Ferrichrome ABC transporter permease protein	0.59	4.64E-04
llmg_2247	Putative uncharacterized protein	0.59	5.72E-04
llmg_1806	Putative uncharacterized protein	0.59	3.03E-04
rmaI	Transcriptional regulator, MarR family	0.59	3.66E-03
ps437	Putative uncharacterized protein ps437	0.59	1.15E-03
llmg_0424	Transcriptional regulator	0.59	9.32E-04
ps517	Putative Dna Primase	0.59	2.66E-04
llmg_1596	UPF0145 protein llmg_1596	0.59	2.48E-04
comGA	Putative competence protein ComGA	0.59	9.56E-05
llmg_0626	Putative transcriptional regulator	0.60	2.90E-04
llmg_1549	Putative uncharacterized protein	0.60	2.20E-04
llmg_1504	Putative uncharacterized protein	0.60	2.35E-03
tetR	Similar to transcriptional regulator	0.60	3.36E-03
cstA	Carbon starvation protein A	0.60	7.10E-04
pbpX	Penicillin-binding protein	0.61	1.60E-03
llmg_0477	Putative membrane protein	0.61	1.16E-03
llmg_2492	Putative uncharacterized protein	0.61	1.22E-03
llmg_0992	Putative nucleoside-diphosphate-sugar epimerases	0.61	2.76E-03
llmg_0183	Putative uncharacterized protein	0.61	2.20E-03
llmg_1359	Putative uncharacterized protein	0.61	1.39E-03
llmg_0568	Putative acylphosphate phosphohydrolase	0.61	1.08E-03
hdiR	HTH-type transcriptional regulator HdiR	0.61	1.37E-03
llmg_1241	Putative uncharacterized protein	0.61	1.19E-03
mesJ	tRNA(Ile)-lysidine synthase	0.61	1.72E-04
llmg_1621	Putative uncharacterized protein	0.61	1.37E-03

llmg_2223	Putative uncharacterized protein	0.62	1.27E-04
llmg_0765	Putative hydrolase	0.62	2.72E-04
llmg_1672	Putative uncharacterized protein	0.62	5.39E-05
llmg_0137	Permeases	0.62	3.76E-04
llmg_0689	Putative uncharacterized protein	0.62	2.91E-04
llmg_2243	Putative uncharacterized protein	0.62	2.53E-05
llmg_2066	Multidrug resistance protein B	0.62	1.45E-03
yfiA	Putative Sigma 54 modulation protein	0.62	1.44E-02
llmg_2207	Putative uncharacterized protein	0.62	2.72E-05
llmg_0692	Putative uncharacterized protein	0.62	5.42E-03
srtC	Sortase SrtC	0.62	3.79E-05
llmg_1197	Putative uncharacterized protein	0.62	4.04E-04
llmg_0956	Transcriptional regulator, LacI family	0.62	3.26E-04
rpmF	50S ribosomal protein L32	0.62	9.23E-03
llmg_0276	Oxidoreductase, aldo/keto reductase family	0.63	1.92E-04
bglH	Beta-glucosidase	0.63	1.65E-03
llmg_1663	Putative uncharacterized protein	0.63	1.56E-02
llmg_1242	Putative uncharacterized protein	0.63	9.13E-03
llmg_0673	Putative membrane protein	0.63	9.21E-04
hrcA	Heat-inducible transcription repressor HrcA	0.63	5.40E-04
llmg_1200	Putative uncharacterized protein	0.63	7.69E-03
llmg_0160	Oxygen-insensitive NAD(P)H nitroreductase	0.63	9.81E-03
cydD	Cytochrome D ABC transporter ATP binding and permease protein	0.63	4.43E-05
llmg_0625	Putative ABC transporter permease protein	0.63	3.61E-05
llmg_1817	Putative beta-phosphoglucomutase	0.63	7.91E-05
llmg_1597	Putative uncharacterized protein	0.64	4.09E-04
llmg_1090	Putative secreted protein	0.64	3.91E-05
pyrB	Aspartate carbamoyltransferase	0.64	5.62E-05
hllA	HU-like DNA-binding protein	0.64	8.20E-03
llmg_0062	Putative membrane protein	0.64	1.29E-03
apbE	Thiamine biosynthesis lipoprotein apbE	0.64	8.73E-06
llmg_0056	Putative uncharacterized protein	0.64	8.50E-04
xylA	Xylose isomerase	0.64	2.12E-02
nrdH	Glutaredoxin-like protein NrdH	0.64	8.86E-03
clpC	ATP-dependent Clp protease ATP-binding subunit	0.64	3.91E-05
noxC	NADH oxidase	0.65	2.30E-05
ceo	N5-carboxyethyl-ornithine synthase	0.65	1.34E-03
butA	Acetoin reductase	0.65	1.12E-05
llmg_1459	Putative Di/Tri-peptide transport system permease protein	0.65	2.92E-04
ctsR	Transcriptional regulator ctsR	0.65	1.18E-04
llmg_2347	Putative uncharacterized protein	0.65	4.04E-05
llmg_1432	Putative secreted protein	0.65	8.85E-04
xseA	Exodeoxyribonuclease 7 large subunit	0.65	1.92E-04
llmg_0670	Putative uncharacterized protein	0.65	1.67E-05
accB	Acetyl-CoA carboxylase biotin carboxyl carrier protein	0.66	1.47E-02
llmg_1245	Putative uncharacterized protein	0.66	4.34E-03

llmg_1215	Putative uncharacterized protein	0.66	4.53E-05
als	Acetolactate synthase large subunit	0.66	1.78E-03
nagA	N-acetylglucosamine-6-phosphate deacetylase	0.66	1.61E-04
llmg_2433	Putative uncharacterized protein	0.67	4.75E-03
llmg_1710	Putative glycosyltransferase	0.67	1.15E-04
llmg_0370	Putative uncharacterized protein	0.67	1.94E-02
llmg_1653	Putative uncharacterized protein	0.67	5.85E-03
llmg_0380	Putative uncharacterized protein	0.67	2.29E-03
noxE	NADH oxidase	0.67	6.44E-04
tagL	Exopolysaccharide biosynthesis protein	0.67	1.76E-04
llmg_1903	Putative transcriptional regulator	0.68	8.67E-05
pcp	Pyrrolidone-carboxylate peptidase	0.68	7.18E-03
llmg_2547	Putative uncharacterized protein	0.68	4.64E-05
llmg_2540	Putative uncharacterized protein	0.68	2.30E-03
galK	Galactokinase	0.68	4.48E-05
ps413	Hypothetical phage protein	0.68	2.55E-02
llmg_0655	Transposase	0.68	3.94E-05
rbsD	D-ribose pyranase	0.68	1.08E-04
llmg_1314	Putative uncharacterized protein	0.68	3.72E-05
llmg_0129	Putative uncharacterized protein	0.69	2.06E-03
llmg_1771	Putative rhodanese-related sulfurtransferase	0.69	3.70E-04
pdhB	Pyruvate dehydrogenase E1 component beta subunit	0.69	5.49E-05
llmg_1751	Alkaline phosphatase superfamily protein	0.69	1.51E-04
aguA	AguA protein	0.69	5.34E-03
llmg_2002	Putative uncharacterized protein	0.69	8.07E-04
llmg_1165	Putative membrane protein	0.69	3.70E-05
llmg_0015	Putative RNA binding protein	0.70	1.60E-03
llmg_1780	Putative uncharacterized protein	0.70	3.03E-02
llmg_0564	Putative soluble lytic murein transglycosylase	0.70	1.79E-03
ps311	Putative uncharacterized protein ps311	0.70	4.21E-03
llmg_0507	Peptide binding protein	0.70	3.19E-05
llmg_0989	ABC transporter ATP binding and permease protein	0.70	3.94E-04
llmg_0087	Putative short-chain type dehydrogenase	0.71	1.05E-03
llmg_1707	Putative glycosyltransferase	0.71	8.07E-04
carA	Carbamoyl-phosphate synthase small chain	0.71	4.01E-05
llmg_0686	Putative uncharacterized protein	0.71	4.54E-06
llmg_1315	Putative RNA methyltransferase	0.71	2.55E-05
nucA	5'-nucleotidase	0.71	1.79E-05
llmg_1054	Putative uncharacterized protein	0.71	1.32E-04
llmg_0961	Sugar kinase and transcriptional regulator	0.71	6.53E-03
llmg_0300	Putative uncharacterized protein	0.71	1.15E-03
llmg_0188	Putative uncharacterized protein	0.72	3.03E-04
ribA	Riboflavin biosynthesis protein ribA	0.72	2.21E-02
ps602	Putative uncharacterized protein ps602	0.72	1.70E-04
llmg_1322	Putative uncharacterized protein	0.72	3.18E-05
llmg_0867	Putative uncharacterized protein	0.72	3.61E-05

llmg_1769	Putative uncharacterized protein	0.73	3.81E-05
ptcB	PTS system, IIB component	0.73	3.97E-04
llmg_2326	Putative membrane protein	0.73	2.12E-06
hisB	Imidazoleglycerol-phosphate dehydratase	0.73	9.04E-04
llmg_0687	Putative uncharacterized protein	0.73	2.16E-06
llmg_1665	Putative membrane protein	0.73	1.58E-05
mtsA	Manganese ABC transporter substrate binding protein	0.73	1.61E-03
llmg_1168	Putative polysaccharide deacetylase	0.73	8.48E-05
accD	Acetyl-coenzyme A carboxylase carboxyl transferase subunit beta	0.74	4.58E-02
oppB2	Peptide transport system permease protein oppB2	0.74	2.81E-04
ps126	Putative DNA primase	0.74	5.40E-04
llmg_0965	Putative uncharacterized protein	0.74	4.83E-04
tnp982	Transposase for insertion sequence element IS982B	0.74	7.67E-04
ps116	Phage repressor	0.74	2.61E-03
llmg_0716	Putative uncharacterized protein	0.74	6.89E-03
ps512	Putative uncharacterized protein ps512	0.74	4.69E-05
llmg_0095	Putative esterase	0.74	4.94E-05
llmg_0925	Putative transcriptional regulator	0.74	1.32E-03
llmg_1495	Putative uncharacterized protein	0.75	1.13E-03
fhuD	Ferrichrome ABC transporter substrate binding protein	0.75	3.95E-05
llmg_2434	Putative uncharacterized protein	0.75	3.79E-04
grpE	Protein GrpE	0.75	2.05E-05
dnaK	Chaperone protein DnaK	0.75	2.12E-05
llmg_0572	Putative transcriptional regulator, MerR family	0.75	1.20E-04
rbsC	Ribose transport system permease protein RbsC	0.76	2.04E-02
llmg_1632	Putative hydrolase	0.76	3.48E-04
llmg_0904	Immunogenic secreted protein homolog	0.76	3.25E-04
llmg_1244	Putative uncharacterized protein	0.76	1.81E-04
llmg_1526	Putative uncharacterized protein	0.76	1.14E-03
aldC	AldC protein	0.76	6.50E-03
ssbA	Single-stranded DNA-binding protein	0.77	5.12E-04
llmg_1260	Putative uncharacterized protein	0.77	3.31E-05
llmg_0696	Putative membrane protein	0.77	1.07E-02
ps357	Putative uncharacterized protein ps357	0.77	2.49E-03
bglS	Beta-glucosidase A	0.78	8.93E-05
llmg_2338	Putative uncharacterized protein	0.78	2.24E-04
llmg_0097	Putative flavoprotein oxygenase	0.78	1.79E-04
llmg_1293	Putative uncharacterized protein	0.78	2.24E-02
llmg_0865	Transcriptional antiterminator, BglG family	0.78	2.53E-05
llmg_1270	Tyrosine recombinase XerS	0.78	1.85E-04
pyrR	Pyrimidine operon regulatory protein	0.78	3.15E-04
llmg_2320	Putative uncharacterized protein	0.78	2.29E-05
nrdF	Ribonucleoside-diphosphate reductase subunit beta	0.78	2.10E-05
mntH	Putative proton-dependent manganese transporter group C beta	0.79	3.10E-03
llmg_0877	Putative secreted protein	0.79	4.94E-05
ccpA	Catabolite control protein A	0.79	1.50E-05

llmg_1629	Putative methyltransferase	0.79	4.20E-03
llmg_0270	Putative uncharacterized protein	0.79	1.86E-05
llmg_1166	Putative endoglucanase	0.79	1.62E-03
llmg_0317	Putative uncharacterized protein	0.79	6.32E-04
llmg_2036	Putative uncharacterized protein	0.79	7.80E-04
ps129	Putative uncharacterized protein ps129	0.80	2.59E-04
llmg_2498	Putative uncharacterized protein	0.80	8.99E-05
pdhA	Pyruvate dehydrogenase E1 component alpha subunit	0.80	1.92E-06
butB	2,3-butanediol dehydrogenase	0.80	1.80E-06
tnp1297	Transposase for insertion sequence element IS1297	0.80	2.65E-03
llmg_1513	Putative uncharacterized protein	0.80	1.29E-03
llmg_2248	Putative abortive phage resistance	0.80	5.27E-05
llmg_0422	Putative uncharacterized protein	0.80	2.90E-04
llmg_1240	Putative uncharacterized protein	0.80	1.31E-05
acmD	N-acetylglucosaminidase	0.80	1.33E-04
llmg_0963	PTS system, IIC component	0.81	2.25E-04
llmg_1658	Putative uncharacterized protein	0.81	7.74E-04
nagB	Glucosamine-6-phosphate deaminase	0.81	8.50E-04
llmg_1804	Transcriptional regulator, rgg family	0.81	1.05E-04
padR	Putative transcriptional repressor of padC	0.81	5.21E-04
llmg_1507	Putative uncharacterized protein	0.81	4.93E-06
trxA	Thioredoxin	0.81	3.91E-04
acmC	N-acetylglucosaminidase	0.82	1.17E-05
gadR	Positive regulator GadR	0.82	1.45E-05
llmg_0189	Putative uncharacterized protein	0.82	2.81E-04
llmg_0880	Putative oxidoreductase	0.83	4.69E-05
napC	Multidrug-efflux transporter protein	0.83	4.46E-03
llmg_1053	Putative uncharacterized protein	0.83	1.06E-05
llmg_0671	Putative uncharacterized protein	0.84	2.52E-05
rmaX	Transcriptional regulator, MarR family	0.84	2.35E-06
llmg_1324	Transcriptional regulator, araC family	0.84	5.85E-04
rbsD	Ribose ABC transporter permease protein RbsD	0.84	6.64E-05
llmg_0470	Putative uncharacterized protein	0.84	1.70E-04
rpmB	50S ribosomal protein L28	0.84	5.59E-04
llmg_2339	Transcriptional regulator	0.84	1.23E-03
llmg_1768	Putative uncharacterized protein	0.85	8.38E-05
drrA	Daunorubicin resistance ABC transporter ATP-binding subunit	0.85	1.61E-04
chb	Chitin binding protein, putative	0.85	1.00E-02
llmg_2032	Putative uncharacterized protein	0.85	6.12E-04
busAA	Glycine betaine/proline ABC transporter	0.85	3.27E-05
llmg_1093	Putative secreted protein	0.85	3.94E-07
llmg_0459	Putative uncharacterized protein	0.85	2.53E-06
llmg_1462	Putative HTH-type transcriptional regulator	0.86	1.35E-05
llmg_0760	Putative transglycosylase	0.86	5.11E-05
llmg_1859	Putative flavodoxin	0.86	3.68E-05
llmg_0968	Oxidoreductase	0.86	4.85E-04

llmg_2548	Putative uncharacterized protein	0.86	6.85E-07
llmg_0651	Putative acetyltransferase	0.86	4.66E-05
llmg_0680	Putative uncharacterized protein	0.86	1.93E-03
rmaA	Transcriptional regulator, MarR family	0.86	9.40E-05
mraW	Ribosomal RNA small subunit methyltransferase H	0.86	3.33E-06
llmg_1257	Putative uncharacterized protein	0.87	2.58E-05
ps508	Hypothetical phage protein predicted by Glimmer/Critica	0.87	1.35E-04
llmg_1159	Putative uncharacterized protein	0.88	1.23E-03
lplL	Lipoate-protein ligase	0.88	2.86E-06
llmg_1772	Putative rhodanese-related sulfurtransferase	0.88	7.31E-05
llmg_0096	Putatve glyoxylase protein	0.88	1.03E-05
llmg_1158	Putative uncharacterized protein	0.88	1.88E-04
llmg_0905	Putative uncharacterized protein	0.89	2.62E-04
llmg_1968	Putative uncharacterized protein	0.89	2.23E-03
llmg_0009	Putative secreted protein	0.89	4.63E-05
llmg_0969	Putative uncharacterized protein	0.89	1.03E-05
llmg_1243	Putative uncharacterized protein	0.89	2.00E-06
tnp946	Transposase for insertion sequence element IS946	0.89	4.93E-06
llmg_1094	Putative secreted protein	0.90	1.66E-04
ps118	Putative uncharacterized protein ps118	0.90	8.98E-04
pdhD	Dihydrolipoyl dehydrogenase	0.90	4.46E-07
ps120	Putative uncharacterized protein ps120	0.91	4.65E-03
pdhC	Pyruvate dehydrogenase complex E2 component	0.91	1.92E-06
glpK	Glycerol kinase	0.91	9.32E-06
llmg_1253	Putative uncharacterized protein	0.92	3.94E-05
llmg_0553	Putative uncharacterized protein	0.92	1.46E-03
llmg_1552	Putative ABC type transport system permease protein	0.92	1.10E-03
llmg_2010	UPF0213 protein llmg_2010	0.92	1.56E-02
xseB	Exodeoxyribonuclease 7 small subunit	0.92	4.87E-04
fruR	Transcriptional regulator of the fructose operon	0.93	9.41E-06
llmg_1205	Putative uncharacterized protein	0.93	1.19E-04
ps121	Putative DNA binding protein	0.93	4.54E-04
llmg_2286	Putative uncharacterized protein	0.93	1.31E-05
llmg_2483	Putative uncharacterized protein	0.93	8.81E-04
llmg_1096	Putative uncharacterized protein	0.94	2.71E-06
llmg_2435	Putative uncharacterized protein	0.94	4.09E-06
fhuC	Ferrichrome ABC transporter fhuC	0.94	1.69E-06
llmg_1236	Putative uncharacterized protein	0.94	3.24E-05
radC	UPF0758 protein llmg_1515	0.96	2.42E-06
rmaB	Transcriptional regulator, MarR family	0.96	6.09E-04
llmg_0432	Similar to transcription regulator	0.96	4.15E-06
hslB	HU-like DNA-binding protein	0.96	7.00E-07
llmg_1857	Putative esterase	0.97	7.00E-07
qor	Quinone oxidoreductase	0.97	4.15E-06
llmg_1095	Putative uncharacterized protein	0.97	3.33E-07
llmg_0089	Putative HTH-type transcriptional regulator	0.97	3.54E-06

tnp1216	Transposase for insertion sequence-like element IS1216	0.97	2.12E-04
llmg_2333	Putative uncharacterized protein	0.98	3.32E-03
dpsA	Non-heme iron-binding ferritin	0.98	2.52E-05
llmg_0589	Putative uncharacterized protein	0.98	4.01E-04
dacA	DacA protein	0.98	6.75E-07
llmg_1210	Multidrug resistance protein	0.98	8.60E-08
ocd	Ornithine cyclodeaminase, mu-crystallin homolog	0.98	5.01E-05
llmg_1079	Putative uncharacterized protein	0.99	2.72E-04
llmg_2218	Predicted transcriptional regulator	0.99	9.68E-07
bglA2	6-phospho-beta-glucosidase	0.99	2.01E-07
llmg_0086	Putative membrane protein	0.99	2.60E-03
ps125	Phage dna replication	1.00	2.36E-04
ps515	Hypothetical phage protein predicted by Glimmer/Critica	1.00	1.14E-04
clpP	ATP-dependent Clp protease proteolytic subunit	1.00	2.37E-06
rmaE	Transcriptional regulator, MarR family	1.01	1.21E-03
llmg_0709	Transcriptional regulator, PadR-like family	1.01	3.94E-05
llmg_1659	Putative uncharacterized protein	1.01	3.92E-05
oppC2	Oligopeptide transport system permease protein oppC2	1.02	1.14E-05
ps509	Hypothetical phage protein predicted by Glimmer/Critica	1.02	5.69E-07
llmg_1052	Putative uncharacterized protein	1.02	2.23E-04
llmg_1091	Putative secreted protein	1.03	1.21E-06
clpB	Chaperone protein ClpB	1.03	1.60E-05
llmg_2438	HTH-type transcriptional regulator	1.03	9.76E-06
ps516	Hypothetical phage protein predicted by Glimmer/Critica	1.03	1.73E-04
llmg_2447	Putative uncharacterized protein	1.04	2.25E-05
ps518	Hypothetical phage protein predicted by Glimmer/Critica	1.04	2.04E-06
llmg_1259	Putative uncharacterized protein	1.04	1.15E-04
llmg_0970	Carboxymuconolactone decarboxylase family protein	1.05	9.47E-07
llmg_1344	Putative uncharacterized protein	1.05	7.32E-07
llmg_1019	Oxidoreductase, short-chain dehydrogenase/reductase family protein	1.05	4.07E-06
llmg_1965	Putative AMP-binding enzyme	1.06	8.22E-07
llmg_1317	Putative N-acetylmannosamine-6-phosphate 2-epimerase	1.06	1.07E-05
cysD	O-acetylhomoserine sulfhydrylase	1.06	3.73E-06
ps511	Putative uncharacterized protein ps511	1.06	4.39E-05
llmg_2146	Putative uncharacterized protein	1.07	1.86E-05
oppA2	Oligopeptide-binding protein oppA2	1.07	4.18E-04
llmg_2441	Putative uncharacterized protein	1.07	4.04E-04
llmg_0685	Putative uncharacterized protein	1.08	1.09E-05
llmg_2249	Putative uncharacterized protein	1.08	2.01E-04
llmg_0866	Putative uncharacterized protein	1.08	1.47E-07
ps609	Putative uncharacterized protein ps609	1.08	4.32E-04
groES	10 kDa chaperonin	1.09	4.07E-06
llmg_1258	Putative uncharacterized protein	1.10	1.27E-06
llmg_0603	HTH-type transcriptional regulator	1.10	3.44E-07
feoA	Ferrous iron transport protein A	1.10	2.10E-07
llmg_1916	Putative electron transport protein	1.10	1.16E-06

ps510	Putative uncharacterized protein ps510	1.10	1.25E-04
llmg_1211	Putative uncharacterized protein	1.10	4.05E-08
galP	Galactose permease	1.11	1.61E-06
chiC	Acidic endochitinase	1.11	4.26E-08
llmg_1130	Putative uncharacterized protein	1.12	1.25E-04
yphI	Putative uncharacterized protein yphI	1.12	1.45E-08
dltE	Oxidoreductase dltE	1.12	8.39E-07
llmg_0245	Putative zinc-binding dehydrogenase	1.13	8.59E-05
ps611	Putative uncharacterized protein ps611	1.13	1.35E-02
llmg_1660	Transcriptional regulator, TetR family	1.13	7.62E-06
fur	Ferric uptake regulation protein	1.14	5.85E-06
arcC2	Carbamate kinase	1.14	3.28E-06
uspA	Universal stress protein A	1.14	2.22E-07
ps122	Putative uncharacterized protein ps122	1.14	2.07E-05
llmg_1800	Putative secreted protein	1.14	2.70E-09
llmg_1920	Putative uncharacterized protein	1.15	2.08E-04
llmg_0439	Transcriptional regulator, LacI family	1.15	1.74E-06
ps340	Putative uncharacterized protein ps340	1.15	5.95E-04
llmg_0354	Putative glyoxylase	1.15	1.00E-07
nadD2	Probable nicotinate-nucleotide adenylyltransferase	1.17	1.19E-05
llmg_0185	Acetyltransferase, GNAT family	1.17	9.20E-08
ps514	Hypothetical phage protein predicted by Glimmer/Critica	1.18	2.36E-07
llmg_1129	Cell wall surface anchor family protein	1.18	3.94E-07
llmg_1563	Putative membrane protein	1.18	8.19E-04
llmg_0248	Transcriptional regulator, TetR family	1.18	1.49E-06
llmg_1461	Putative Di/Tri-peptide binding protein	1.18	7.53E-05
llmg_2145	Putative uncharacterized protein	1.19	4.61E-07
lysQ	Amino-acid permease lysQ	1.19	3.69E-07
llmg_1802	Putative uncharacterized protein	1.19	4.68E-06
ps513	Putative uncharacterized protein ps513	1.19	6.80E-07
glpD	GlpD protein	1.20	4.08E-04
llmg_0972	Putative uncharacterized protein	1.21	2.55E-07
llmg_1092	Putative uncharacterized protein	1.21	4.14E-09
htrA	Serine protease Do-like HtrA	1.21	2.70E-09
llmg_1774	Putative uncharacterized protein	1.22	6.02E-03
llmg_1676	ABC transporter permease protein	1.22	2.71E-07
ps519	Putative Terminase small subunit	1.23	4.54E-06
ps123	Putative uncharacterized protein ps123	1.25	3.68E-05
llmg_1141	Putative transcriptional regulator	1.25	1.92E-05
llmg_1460	Putative Di/Tri-peptide transport ATP-binding protein	1.26	2.22E-07
llmg_1773	Putative uncharacterized protein	1.27	1.15E-04
llmg_0353	Putative transcription regulator	1.29	9.66E-07
llmg_1675	ABC transporter ATP-binding protein	1.29	3.55E-06
llmg_1917	Putative uncharacterized protein	1.30	2.84E-08
llmg_2144	Putative uncharacterized protein	1.30	1.94E-08
llmg_1803	Putative secreted protein	1.30	7.61E-07

comX	Competence regulator ComX	1.31	5.61E-06
ps119	Putative uncharacterized protein ps119	1.32	1.66E-04
ps117	Putative uncharacterized protein ps117	1.32	1.12E-06
uspA2	Universal stress protein A2	1.32	7.88E-07
groEL2	60 kDa chaperonin	1.33	8.70E-08
llmg_0527	Putative uncharacterized protein	1.36	4.40E-08
llmg_1458	Putative Di/Tri-peptide transport system permease protein	1.37	1.57E-06
glpF2	Glycerol uptake facilitator	1.38	7.25E-08
llmg_1139	Putative uncharacterized protein	1.38	3.44E-06
llmg_2436	Putative uncharacterized protein	1.38	5.42E-07
osmC	Osmotically inducible protein C	1.40	3.78E-07
llmg_1454	Putative sugar kinase	1.41	7.61E-07
llmg_1128	Putative uncharacterized protein	1.41	1.02E-05
bglX	Beta-glucosidase	1.43	2.45E-08
llmg_1182	Putative acetyltransferase	1.44	9.63E-06
llmg_2016	Putative uncharacterized protein	1.44	7.00E-07
llmg_2143	Putative 20-kDa protein	1.44	2.95E-09
clpE	ATP-dependent Clp protease ATP-binding subunit ClpE	1.44	4.93E-06
ps407	Putative uncharacterized protein ps407	1.45	2.12E-05
arcA	Arginine deiminase	1.46	2.72E-08
arcD1	Arginine/ornithine antiporter	1.46	4.48E-07
llmg_1050	Putative secreted protein	1.47	1.51E-04
llmg_1904	Putative nitroreductase	1.47	1.74E-06
arcB	Ornithine carbamoyltransferase, catabolic	1.47	2.55E-07
arcC1	Carbamate kinase	1.49	3.74E-09
llmg_1140	Putative uncharacterized protein	1.49	8.80E-08
llmg_1553	Putative ABC transporter ATP-binding protein	1.49	6.31E-10
llmg_1457	Putative Di/Tri-peptide transport ATP-binding protein	1.51	1.34E-06
llmg_0962	Transcriptional regulator, araC family	1.51	1.17E-09
llmg_1127	Cell wall surface anchor family protein	1.53	7.22E-06
llmg_0155	Putative uncharacterized protein	1.54	4.94E-09
llmg_2481	Putative chorismate mutase	1.60	1.22E-05
cbr	Carbonyl reductase cbr	1.62	4.03E-09
llmg_0246	Putative uncharacterized protein	1.68	6.52E-10
comFC	Putative late competence protein	1.74	2.46E-05
llmg_1703	Putative uncharacterized protein	1.79	1.33E-06
llmg_2426	Putative uncharacterized protein	1.79	1.76E-04
llmg_0991	Transcriptional regulator, AcrR family	1.80	1.33E-08
llmg_1915	Putative Fe-S oxidoreductase	1.83	1.20E-10
rmeC	Transcriptional regulator, MerR family	1.85	9.79E-10
llmg_0959	Beta-glucosidase	1.87	2.16E-10
llmg_0247	Putative UDP-glucose 4-epimerase	1.91	2.97E-10
llmg_1235	Cyclic nucleotide-binding domain protein	1.96	8.70E-08
llmg_1761	Putative uncharacterized protein	2.07	1.02E-07
rmeB	Transcriptional regulator, MerR family	2.11	6.93E-08
llmg_0960	Beta-glucosidase	2.12	1.58E-12

llmg_0458	Glucosyltransferase-I	2.16	5.39E-10
llmg_0485	Putative membrane protein	2.24	4.21E-11
rpe2	Ribulose-phosphate 3-epimerase	2.25	4.39E-12
rpiB	Ribose 5-phosphate isomerase B	2.30	2.61E-10
llmg_0486	Putative membrane protein	2.81	1.39E-14
llmg_0489	Sugar transport system permease protein	3.03	4.44E-14
llmg_0490	Sugar transport system permease protein	3.11	1.88E-12
llmg_0488	Multiple sugar-binding protein	3.85	4.57E-15
llmg_0487	Putative trehalose/maltose hydrolase	4.04	8.45E-16

Table S3. Differentially expressed genes at 38°C (TM29 vs. MG1363)

Gene name	Protein name	logFC	adj.P.Val
llmg_1358	Putative uncharacterized protein	-5.72	3.15E-15
llmg_1355	Putative uncharacterized protein	-5.72	2.50E-14
telA	Putative tellurium resistance protein	-5.67	6.29E-14
llmg_1353	Putative tellurite resistance protein	-5.64	5.03E-15
llmg_1357	Putative uncharacterized protein	-5.63	5.03E-15
llmg_1356	Putative uncharacterized protein	-5.62	5.03E-15
llmg_1354	Putative uncharacterized protein	-5.52	1.75E-14
telB	Putative tellurium resistance protein	-5.41	5.21E-14
telC	Putative tellurium resistance protein	-5.31	5.03E-15
llmg_1349	Putative uncharacterized protein	-4.96	6.18E-13
ps431	Putative uncharacterized protein ps431	-3.53	2.11E-09
ribB	RibB protein	-2.68	2.64E-07
ribA	Riboflavin biosynthesis protein ribA	-2.55	9.66E-07
ribD	Riboflavin biosynthesis protein RibD	-2.27	1.34E-08
llmg_0487	Putative trehalose/maltose hydrolase	-2.03	2.05E-11
llmg_0488	Multiple sugar-binding protein	-2.00	3.02E-10
llmg_1529	6,7-dimethyl-8-ribityllumazine synthase	-1.79	7.15E-06
llmg_0486	Putative membrane protein	-1.78	6.49E-11
llmg_0247	Putative UDP-glucose 4-epimerase	-1.72	1.15E-08
llmg_0246	Putative uncharacterized protein	-1.72	4.86E-09
llmg_0760	Putative transglycosylase	-1.64	1.68E-07
llmg_0485	Putative membrane protein	-1.58	3.90E-08
llmg_1019	Oxidoreductase, short-chain dehydrogenase/reductase family protein	-1.58	1.78E-07
llmg_0489	Sugar transport system permease protein	-1.56	4.95E-09
msrA	Peptide methionine sulfoxide reductase MsrA	-1.54	3.84E-06
tpx	Thiol peroxidase	-1.53	1.31E-08
llmg_0490	Sugar transport system permease protein	-1.49	2.85E-07
llmg_0275	Putative uncharacterized protein	-1.45	1.82E-06
argC	N-acetyl-gamma-glutamyl-phosphate reductase	-1.42	1.27E-04
llmg_1269	Putative uncharacterized protein	-1.42	4.50E-05
llmg_0245	Putative zinc-binding dehydrogenase	-1.40	2.65E-05
argD	Acetylornithine aminotransferase	-1.39	1.83E-04
argJ	Arginine biosynthesis bifunctional protein ArgJ	-1.37	1.05E-04
argB	Acetylglutamate kinase	-1.37	3.12E-04
llmg_1135	Putative uncharacterized protein	-1.34	7.15E-06
llmg_0676	Hypothetical acetyltransferase	-1.33	4.49E-07
gpo	Glutathione peroxidase	-1.31	6.57E-07
llmg_1253	Putative uncharacterized protein	-1.31	2.81E-06
llmg_1252	Putative uncharacterized protein	-1.31	4.26E-05
llmg_1675	ABC transporter ATP-binding protein	-1.29	1.20E-05
argG	Argininosuccinate synthase	-1.28	2.07E-06
mapA	Maltose phosphorylase	-1.28	6.60E-07
rmaD	Transcriptional regulator, MarR family	-1.26	8.78E-04

argF	Ornithine carbamoyltransferase, catabolic	-1.24	8.78E-04
noxE	NADH oxidase	-1.24	4.23E-06
llmg_0274	Putative uncharacterized protein	-1.24	1.76E-05
llmg_1676	ABC transporter permease protein	-1.24	1.04E-06
llmg_0568	Putative acylphosphate phosphohydrolase	-1.23	3.10E-06
tnp982	Transposase for insertion sequence element IS982B	-1.23	1.58E-05
thyA	Thymidylate synthase	-1.23	9.66E-07
llmg_1774	Putative uncharacterized protein	-1.23	1.09E-02
llmg_1803	Putative secreted protein	-1.22	5.46E-06
llmg_1093	Putative secreted protein	-1.21	2.98E-08
llmg_0087	Putative short-chain type dehydrogenase	-1.18	2.28E-05
pyrE	Orotate phosphoribosyltransferase	-1.17	4.27E-05
llmg_1089	Carbamoyl-phosphate synthase large chain	-1.16	7.45E-04
llmg_1094	Putative secreted protein	-1.16	3.46E-05
argH	Argininosuccinate lyase	-1.15	2.95E-05
llmg_1773	Putative uncharacterized protein	-1.15	7.11E-04
llmg_0880	Putative oxidoreductase	-1.14	4.70E-06
llmg_1549	Putative uncharacterized protein	-1.14	8.35E-07
llmg_2145	Putative uncharacterized protein	-1.13	3.12E-06
llmg_0089	Putative HTH-type transcriptional regulator	-1.13	2.54E-06
llmg_2547	Putative uncharacterized protein	-1.13	6.38E-07
malE	Maltose ABC transporter substrate binding protein	-1.13	5.93E-06
llmg_0998	Putative hydrolase	-1.12	9.19E-07
llmg_1092	Putative uncharacterized protein	-1.12	1.01E-07
llmg_1977	Putative MecA homolog	-1.10	3.54E-06
llmg_0276	Oxidoreductase, aldo/keto reductase family	-1.09	2.06E-06
ps311	Putative uncharacterized protein ps311	-1.08	2.87E-04
llmg_0865	Transcriptional antiterminator, BglG family	-1.08	2.64E-06
llmg_1095	Putative uncharacterized protein	-1.08	4.33E-07
pyrDB	Dihydroorotate dehydrogenase B (NAD(+)), catalytic subunit	-1.08	3.30E-05
dexC	Neopullulanase	-1.07	3.53E-06
qor	Quinone oxidoreductase	-1.06	4.92E-06
tetR	Similar to transcriptional regulator	-1.06	6.28E-05
pepP	Aminopeptidase P	-1.05	3.61E-06
llmg_1710	Putative glycosyltransferase	-1.05	3.24E-06
purK	Phosphoribosylaminoimidazole carboxylase ATPase subunit	-1.04	6.58E-05
cfa	Cyclopropane-fatty-acyl-phospholipid synthase	-1.03	5.34E-07
llmg_1802	Putative uncharacterized protein	-1.02	7.07E-05
llmg_1240	Putative uncharacterized protein	-1.02	3.28E-06
llmg_1852	Putative uncharacterized protein	-1.01	1.20E-05
purD	Phosphoribosylamine--glycine ligase	-1.01	8.66E-06
llmg_0965	Putative uncharacterized protein	-1.00	7.10E-05
llmg_2548	Putative uncharacterized protein	-0.99	5.62E-07
llmg_0655	Transposase	-0.98	2.65E-06
llmg_1708	Putative glycosyltransferase	-0.97	2.72E-03
llmg_1827	Putative acetyltransferase	-0.97	5.74E-07

ctpE	Cation-transporting ATPase, E1-E2 family	-0.95	5.36E-06
llmg_1090	Putative secreted protein	-0.95	1.80E-06
llmg_0904	Immunogenic secreted protein homolog	-0.95	9.96E-05
llmg_0177	Amidase	-0.95	4.70E-06
llmg_0270	Putative uncharacterized protein	-0.93	9.88E-06
osmC	Osmotically inducible protein C	-0.93	1.02E-04
pyrK	Dihydroorotate dehydrogenase B (NAD(+)), electron transfer subunit	-0.93	7.71E-06
llmg_1826	Putative uncharacterized protein	-0.93	1.11E-05
llmg_2334	Putative uncharacterized protein	-0.92	5.45E-04
llmg_2146	Putative uncharacterized protein	-0.92	2.24E-04
maa2	Maltose O-acetyltransferase	-0.92	1.99E-04
llmg_1800	Putative secreted protein	-0.92	2.92E-07
dnaD	DNA replication protein dnaD	-0.92	3.10E-07
llmg_1548	Putative phosphohydrolase	-0.92	2.63E-06
llmg_1148	Putative uncharacterized protein	-0.91	2.19E-04
rluA	Pseudouridine synthase	-0.91	2.72E-03
llmg_2144	Putative uncharacterized protein	-0.91	6.02E-06
uspA2	Universal stress protein A2	-0.91	1.38E-04
fhuC	Ferrichrome ABC transporter fhuC	-0.90	9.25E-06
llmg_1919	Putative uncharacterized protein	-0.89	7.37E-05
dexA	Oligo-1,6-alpha-glucosidase	-0.89	2.64E-04
srtC	Sortase SrtC	-0.89	2.73E-06
rpe2	Ribulose-phosphate 3-epimerase	-0.89	4.35E-06
asnB	Asparagine synthetase	-0.89	3.37E-03
rrmA	Putative rRNA (Guanine-N1-)-methyltransferase	-0.89	3.55E-05
nth	Putative endonuclease III	-0.88	4.03E-06
pyrC	Dihydroorotase	-0.88	5.91E-05
purE	N5-carboxyaminoimidazole ribonucleotide mutase	-0.88	1.12E-05
llmg_0328	Putative uncharacterized protein	-0.88	1.08E-03
hslO	33 kDa chaperonin	-0.88	3.66E-06
malF	Maltose transport system permease protein malF	-0.87	1.86E-05
ssbA	Single-stranded DNA-binding protein	-0.86	4.52E-04
pyrF	Orotidine 5'-phosphate decarboxylase	-0.86	1.83E-04
llmg_1597	Putative uncharacterized protein	-0.86	6.28E-05
llmg_0883	Putative uncharacterized protein	-0.86	2.56E-04
llmg_2143	Putative 20-kDa protein	-0.86	7.93E-06
rnhA	Ribonuclease HIII	-0.85	1.30E-06
llmg_0548	Putative uncharacterized protein	-0.85	1.18E-05
llmg_0552	Putative glyoxylase	-0.85	2.72E-03
malG	Maltose ABC transporter permease protein malG	-0.84	3.62E-04
llmg_1205	Putative uncharacterized protein	-0.84	7.03E-04
tnp946	Transposase for insertion sequence element IS946	-0.84	3.06E-05
llmg_1707	Putative glycosyltransferase	-0.84	4.30E-04
pyrP	Uracil permease	-0.84	2.04E-05
pcp	Pyrrolidone-carboxylate peptidase	-0.84	3.36E-03
llmg_1147	Putative transcription regulator	-0.84	5.91E-05

rlrG	Transcriptional regulator, LysR family	-0.83	6.17E-05
llmg_2015	Putative uncharacterized protein	-0.83	7.93E-06
llmg_1091	Putative secreted protein	-0.83	3.84E-05
carA	Carbamoyl-phosphate synthase small chain	-0.83	2.38E-05
llmg_0394	Putative permease protein	-0.83	3.46E-05
llmg_0160	Oxygen-insensitive NAD(P)H nitroreductase	-0.82	3.36E-03
llmg_0866	Putative uncharacterized protein	-0.81	1.45E-05
llmg_1432	Putative secreted protein	-0.81	3.23E-04
llmg_1242	Putative uncharacterized protein	-0.80	3.44E-03
llmg_0317	Putative uncharacterized protein	-0.80	1.31E-03
llmg_0431	FMN-dependent NADH-azoreductase	-0.80	1.14E-04
eraL	GTPase Era	-0.80	1.23E-04
llmg_0422	Putative uncharacterized protein	-0.80	7.16E-04
llmg_1825	Putative uncharacterized protein	-0.80	2.65E-05
ps429	Putative uncharacterized protein ps429	-0.80	1.43E-03
llmg_0551	Putative membrane protein	-0.79	1.06E-04
llmg_0992	Putative nucleoside-diphosphate-sugar epimerases	-0.79	7.50E-04
amyY	Alpha-amylase	-0.79	9.15E-05
llmg_2211	Putative uncharacterized protein	-0.77	9.89E-03
llmg_0432	Similar to transcription regulator	-0.77	1.22E-04
cmhR	Putative HTH-type transcriptional regulator cmhR	-0.77	7.88E-06
llmg_1462	Putative HTH-type transcriptional regulator	-0.77	1.14E-04
llmg_1257	Putative uncharacterized protein	-0.77	2.29E-04
kdgR	GntR family transcription regulator	-0.77	3.84E-04
llmg_1914	Putative uncharacterized protein	-0.76	4.73E-03
llmg_1243	Putative uncharacterized protein	-0.76	3.44E-05
llmg_1306	Putative uncharacterized protein	-0.76	7.70E-05
vacB2	Ribonuclease R	-0.76	2.40E-03
azlC	Branched-chain amino acid transport protein azlC	-0.76	4.40E-06
llmg_2014	Putative uncharacterized protein	-0.75	4.23E-04
llmg_1908	Putative methyltransferase	-0.75	2.49E-04
pyrR	Pyrimidine operon regulatory protein	-0.75	1.04E-03
llmg_1096	Putative uncharacterized protein	-0.74	9.71E-05
folD	Bifunctional protein FolD	-0.74	6.77E-04
mutT	Putative mutator protein	-0.74	3.12E-04
llmg_0114	Glycosyl transferase, family 2	-0.74	6.14E-04
llmg_1553	Putative ABC transporter ATP-binding protein	-0.74	1.65E-05
hdiR	HTH-type transcriptional regulator HdiR	-0.73	6.87E-04
llmg_1911	Putative uncharacterized protein	-0.73	9.92E-04
llmg_2326	Putative membrane protein	-0.73	7.06E-06
murM	Putative peptidoglycan branched peptide synthesis protein	-0.73	1.14E-02
dppD	Dipeptide transport ATP-binding protein dppD	-0.73	1.28E-03
llmg_0991	Transcriptional regulator, AcrR family	-0.73	8.94E-04
llmg_0329	ABC transporter ATP binding and permease protein	-0.72	1.71E-04
agl	Exo-alpha-1,4-glucosidase	-0.72	9.60E-03
aroE	Shikimate dehydrogenase	-0.72	3.99E-05

llmg_1425	Transcriptional regulator, RpiR family	-0.72	2.65E-05
pA	Lipoprotein signal peptidase	-0.72	8.26E-06
pbp2A	Penicillin-binding protein 2a	-0.72	2.26E-05
llmg_0708	Putative uncharacterized protein	-0.72	2.17E-04
llmg_1241	Putative uncharacterized protein	-0.71	7.58E-04
llmg_1677	Putative secreted protein	-0.71	3.66E-04
llmg_0945	Putative glycerol dehydrogenase	-0.71	1.58E-05
llmg_0882	Putative membrane protein	-0.71	7.17E-05
menX	Menaquinone biosynthesis related protein	-0.71	3.52E-04
pepO2	Putative neutral endopeptidase O2	-0.71	3.52E-04
llmg_1500	Putative methyltransferase	-0.71	3.22E-05
llmg_1128	Putative uncharacterized protein	-0.70	9.74E-03
llmg_1984	Transcriptional regulator	-0.70	1.65E-03
mycA	Myosin-crossreactive streptococcal antigen homologue	-0.70	9.18E-03
llmg_1316	Putative transcriptional regulator	-0.70	4.60E-03
ps407	Putative uncharacterized protein ps407	-0.69	1.83E-02
llmg_1746	Putative hydrolase	-0.69	8.86E-06
purS	Phosphoribosylformylglycinamidine synthetase PurS	-0.69	4.98E-04
llmg_1127	Cell wall surface anchor family protein	-0.69	1.49E-02
maa	Maltose O-acetyltransferase	-0.69	1.93E-03
pmsR	Peptide methionine sulfoxide reductase MsrA	-0.69	1.50E-03
glgC	Glucose-1-phosphate adenylyltransferase	-0.69	2.48E-03
fhuB	Ferrichrome ABC transporter permease protein	-0.69	3.95E-03
dapA	Dihydrodipicolinate synthase	-0.68	5.24E-03
mleS	Malolactic enzyme	-0.68	1.65E-04
guaB	Inosine-5'-monophosphate dehydrogenase	-0.68	4.58E-03
hsdR	Type I restriction-modification system restriction subunit	-0.68	3.17E-04
rluD	Pseudouridine synthase	-0.67	6.18E-03
acmC	N-acetylglucosaminidase	-0.67	2.33E-04
ctsR	Transcriptional regulator ctsR	-0.67	2.18E-04
llmg_1769	Putative uncharacterized protein	-0.67	2.24E-04
hemN	Coproporphyrinogen III oxidase	-0.67	1.88E-03
llmg_0458	Glucosyltransferase-I	-0.66	1.28E-03
llmg_1772	Putative rhodanese-related sulfurtransferase	-0.66	2.23E-03
smpB	SsrA-binding protein	-0.65	6.41E-04
llmg_1903	Putative transcriptional regulator	-0.65	3.09E-04
llmg_1771	Putative rhodanese-related sulfurtransferase	-0.65	1.39E-03
llmg_2424	Putative uncharacterized protein	-0.65	4.68E-05
llmg_2471	Putative uncharacterized protein	-0.64	4.81E-04
llmg_1108	Putative membrane protein	-0.64	1.14E-02
llmg_0471	Putative uncharacterized protein	-0.64	3.66E-02
llmg_0903	Putative serine/threonine phosphatase	-0.64	2.09E-03
nadD2	Probable nicotinate-nucleotide adenylyltransferase	-0.64	5.75E-03
epsR	Transcriptional regulator	-0.64	6.85E-04
llmg_1467	Putative ABC transporter permease protein	-0.64	2.58E-03
argE	Acetylornithine deacetylase	-0.64	6.67E-04

llmg_1937	Putative uncharacterized protein	-0.63	1.95E-03
llmg_1594	Gamma-glutamyl-diamino acid-endopeptidase	-0.63	7.52E-04
llmg_1468	Putative ABC transporter ATP binding protein	-0.63	9.01E-05
llmg_1853	ABC transporter ATP-binding and permease protein	-0.63	8.31E-05
llmg_0867	Putative uncharacterized protein	-0.63	3.45E-04
llmg_1745	Putative membrane protein	-0.63	1.94E-04
llmg_1554	Transcriptional regulator, TetR family	-0.63	1.60E-03
llmg_1766	Putative acetyltransferase	-0.63	4.41E-04
llmg_1629	Putative methyltransferase	-0.63	2.94E-02
ubiE	Demethylmenaquinone methyltransferase	-0.63	3.92E-04
llmg_1348	Putative uncharacterized protein	-0.63	7.53E-03
tkt	Tkt protein	-0.62	3.55E-03
llmg_1235	Cyclic nucleotide-binding domain protein	-0.62	1.31E-02
glgD	Glucose-1-phosphate adenylyltransferase	-0.62	1.30E-02
llmg_0959	Beta-glucosidase	-0.62	3.62E-04
llmg_0564	Putative soluble lytic murein transglycosylase	-0.62	8.46E-03
llmg_1160	Putative uncharacterized protein	-0.62	2.64E-05
llmg_0938	Putative uncharacterized protein	-0.62	9.81E-04
ps434	Putative uncharacterized protein ps434	-0.62	9.06E-03
cspC	Cold shock protein cspC	-0.62	2.92E-02
llmg_2280	UPF0346 protein llmg_2280	-0.61	3.47E-04
llmg_0995	Hydrolase, haloacid dehalogenase-like family protein	-0.61	9.92E-04
llmg_0884	Putative uncharacterized protein	-0.61	9.01E-05
pepF	PepF protein	-0.61	1.48E-03
thiN	Thiamin pyrophosphokinase	-0.61	3.96E-05
llmg_2498	Putative uncharacterized protein	-0.61	2.32E-03
llmg_1976	Polysaccharide biosynthesis protein	-0.61	6.82E-05
sunL	Ribosomal RNA small subunit methyltransferase B	-0.61	7.60E-04
gyrB	DNA gyrase subunit B	-0.60	6.67E-04
llmg_0146	Aryl-alcohol dehydrogenase	-0.60	7.10E-03
llmg_1245	Putative uncharacterized protein	-0.60	1.39E-02
llmg_1635	Putative ABC transporter ATP-binding protein	-0.60	1.03E-04
purC	Phosphoribosylaminoimidazole-succinocarboxamide synthase	-0.60	1.86E-03
ps437	Putative uncharacterized protein ps437	-0.60	2.39E-03
llmg_1369	Putative uncharacterized protein	-0.59	2.86E-04
llmg_1526	Putative uncharacterized protein	-0.59	1.28E-02
llmg_0380	Putative uncharacterized protein	-0.59	1.05E-02
llmg_0279	Putative uncharacterized protein	-0.59	3.37E-03
murD	UDP-N-acetylmuramoylalanine--D-glutamate ligase	-0.59	1.41E-04
dapB	Dihydrodipicolinate reductase	-0.59	3.12E-04
amd	Amidase/aminoacylase	-0.59	1.83E-04
llmg_0162	Putative uncharacterized protein	-0.59	3.53E-04
gltS	Arginine-binding periplasmic protein 1	-0.59	2.68E-03
hipO1	N-acetyldiaminopimelate deacetylase	0.59	5.90E-04
llmg_0173	Putative uncharacterized protein	0.59	5.14E-04
llmg_1956	Putative uncharacterized protein	0.59	1.18E-04

rnhB	Ribonuclease HII	0.59	3.66E-03
thrB	Homoserine kinase	0.59	1.42E-02
llmg_1747	Amino acid permease	0.59	1.88E-02
llmg_1665	Putative membrane protein	0.59	3.53E-04
llmg_0398	Putative uncharacterized protein	0.59	1.67E-04
leuS	Leucine--tRNA ligase	0.59	1.60E-04
plpA	Lipoprotein	0.59	9.98E-05
llmg_0097	Putative flavoprotein oxygenase	0.60	3.72E-03
mutS	DNA mismatch repair protein MutS	0.60	4.15E-04
llmg_1397	Putative uncharacterized protein	0.60	2.93E-04
llmg_2065	Oxidoreductase, aldo/keto reductase family	0.60	1.19E-03
uxuA	Mannonate dehydratase	0.60	2.55E-02
pcaC	Gamma-carboxymuconolactone decarboxylase	0.60	4.42E-03
llmg_2452	Putative uncharacterized protein	0.60	1.32E-03
frdC	Fumarate reductase flavoprotein subunit	0.60	2.19E-04
llmg_2249	Putative uncharacterized protein	0.61	2.65E-02
llmg_0493	Putative esterase/lipase	0.61	3.69E-04
llmg_1581	UPF0210 protein llmg_1581	0.61	6.85E-05
gidA	tRNA uridine 5-carboxymethylaminomethyl modification enzyme MnmG	0.61	4.93E-04
ackA1	Acetate kinase	0.61	2.58E-03
llmg_0877	Putative secreted protein	0.61	1.31E-03
llmg_1667	Glycosyltransferase	0.61	1.43E-03
rgpE	Glycosyltransferase RgpE	0.61	1.89E-03
llmg_0198	Putative uncharacterized protein	0.61	5.90E-04
llmg_2461	Putative uncharacterized protein	0.61	3.95E-05
llmg_1155	Glutaminase	0.61	2.29E-02
llmg_2232	Putative uncharacterized protein	0.61	4.41E-03
llmg_0076	High confidence in function and specificity	0.62	1.16E-03
llmg_0695	Putative uncharacterized protein	0.62	2.79E-02
llmg_2500	Putative uncharacterized protein	0.62	5.67E-04
llmg_0284	Putative uncharacterized protein	0.62	1.09E-03
lmrC	Multidrug resistance protein C	0.62	1.01E-03
rgpC	Capsule polysaccharide export inner-membrane protein RgpC	0.62	1.06E-04
pgsA	CDP-diacylglycerol--glycerol-3-phosphate 3-phosphatidyltransferase	0.62	5.24E-03
llmg_2297	Putative uncharacterized protein	0.62	3.60E-04
copA	Copper/potassium-transporting ATPase	0.62	1.33E-02
llmg_1174	Putative uncharacterized protein	0.62	2.29E-04
llmg_1379	Predicted Zn peptidase	0.62	2.21E-03
llmg_1395	Putative uncharacterized protein	0.64	5.45E-05
llmg_1076	Putative acetyltransferase	0.64	3.55E-04
llmg_1613	Putative secreted protein	0.64	1.98E-02
polC	DNA polymerase III PolC-type	0.64	1.99E-04
wefC	Putative uncharacterized protein wefC	0.64	5.28E-04
nagZ	Putative beta-N-acetylglucosaminidase	0.64	6.17E-05
ps603	Putative uncharacterized protein ps603	0.64	1.29E-03
ps512	Putative uncharacterized protein ps512	0.64	5.14E-04

llmg_0592	Putative uncharacterized protein	0.64	2.29E-04
llmg_0687	Putative uncharacterized protein	0.64	3.06E-05
llmg_2036	Putative uncharacterized protein	0.64	7.84E-03
llmg_2439	Putative uncharacterized protein	0.64	1.81E-02
llmg_1201	Putative uncharacterized protein	0.64	9.89E-05
ps604	Putative uncharacterized protein ps604	0.64	2.95E-05
dukB	Deoxynucleoside kinase	0.64	1.25E-04
llmg_0510	Putative Zn-dependent hydrolase of metallo-beta-lactamase superfamily	0.64	1.21E-05
llmg_2340	Putative uncharacterized protein	0.65	4.58E-03
nifZ	Pyridoxal-phosphate dependent aminotransferase	0.65	1.70E-04
oxaA2	Membrane protein insertase YidC	0.65	7.27E-05
ps119	Putative uncharacterized protein ps119	0.65	4.34E-02
dnaE	DNA polymerase III alpha subunit	0.65	6.29E-04
pgmA	Alpha-phosphoglucomutase	0.66	1.64E-03
ceo	N5-carboxyethyl-ornithine synthase	0.66	2.47E-03
llmg_0688	Putative uncharacterized protein	0.66	1.16E-03
ptnD	PTS system, mannose-specific IID component	0.66	4.72E-02
llmg_1200	Putative uncharacterized protein	0.66	1.06E-02
xpt	Xanthine phosphoribosyltransferase	0.66	9.43E-05
ps117	Putative uncharacterized protein ps117	0.66	2.58E-03
glnP	Glutamine ABC transporter permease and substrate binding protein	0.66	4.89E-04
llmg_0680	Putative uncharacterized protein	0.66	2.07E-02
llmg_0009	Putative secreted protein	0.66	1.60E-03
ywjF	Putative 3-hydroxyisobutyrate dehydrogenase	0.67	6.55E-05
ps612	Putative uncharacterized protein ps612	0.67	4.50E-04
llmg_0381	Putative membrane protein	0.67	2.51E-03
ps120	Putative uncharacterized protein ps120	0.67	4.39E-02
thiD1	Phosphomethylpyrimidine kinase	0.68	5.94E-04
llmg_1236	Putative uncharacterized protein	0.68	1.54E-03
llmg_2294	Putative uncharacterized protein	0.68	8.62E-04
mntH	Putative proton-dependent manganese transporter group C beta	0.69	1.45E-02
rcfB	Transcriptional regulator	0.69	6.54E-05
ps510	Putative uncharacterized protein ps510	0.69	1.04E-02
llmg_0220	Putative glycosyl transferase	0.70	2.01E-04
llmg_0526	Putative membrane protein	0.70	2.93E-03
llmg_2563	Putative uncharacterized protein	0.70	5.15E-04
ps509	Hypothetical phage protein predicted by Glimmer/Critica	0.70	1.06E-04
poxL	Pyruvate oxidase	0.71	4.69E-04
oppC2	Oligopeptide transport system permease protein oppC2	0.71	9.81E-04
trpS	Tryptophan--tRNA ligase	0.71	6.15E-05
llmg_1198	Putative uncharacterized protein	0.71	3.22E-05
ps605	Putative uncharacterized protein ps605	0.71	2.62E-04
llmg_0689	Putative uncharacterized protein	0.72	1.86E-04
ps123	Putative uncharacterized protein ps123	0.72	8.50E-03
gpmB	Phosphoglycerate mutase	0.72	9.01E-05
llmg_2184	Putative uncharacterized protein	0.72	1.83E-04

thiM	Hydroxyethylthiazole kinase	0.72	7.03E-04
gapA	Glyceraldehyde 3-phosphate dehydrogenase	0.72	2.56E-04
glf1	Glf1 protein	0.73	2.26E-05
llmg_1512	Putative ABC transporter ATP-binding protein	0.73	2.01E-04
eriC	Putative chloride channel protein	0.73	2.14E-04
menC	O-succinylbenzoate synthase	0.73	1.97E-02
rbsB	Ribose ABC transporter substrate binding protein RbsB	0.73	7.39E-04
llmg_0899	Putative uncharacterized protein	0.73	3.72E-05
pepC	PepC protein	0.73	7.64E-04
llmg_1497	Putative hydrolase	0.73	1.33E-05
hadL	Cryptic haloacid dehalogenase 1	0.73	8.94E-04
llmg_2163	Putative uncharacterized protein	0.73	3.98E-04
ps121	Putative DNA binding protein	0.74	5.86E-03
llmg_2456	Putative membrane protein	0.74	8.92E-05
llmg_0522	Putative transcriptional regulator	0.74	3.33E-06
ps508	Hypothetical phage protein predicted by Glimmer/Critica	0.74	1.39E-03
llmg_2531	Putative uncharacterized protein	0.74	2.01E-04
rbsD	D-ribose pyranase	0.75	1.18E-04
nagB	Glucosamine-6-phosphate deaminase	0.75	3.36E-03
metB1	Cystathionine gamma-synthase	0.75	8.92E-05
llmg_0683	Putative transposase helper protein for IS712A	0.75	3.11E-03
llmg_1407	Putative uncharacterized protein	0.75	1.31E-04
ps357	Putative uncharacterized protein ps357	0.75	6.36E-03
llmg_1086	Similar to cation (Calcium) transporting ATPase	0.75	1.10E-03
llmg_1029	Putative membrane protein	0.76	1.16E-04
nhoA	Arylamine N-acetyltransferase 2	0.76	6.80E-05
llmg_2324	Putative uncharacterized protein	0.76	3.15E-05
llmg_2325	Putative uncharacterized protein	0.76	4.04E-04
llmg_1480	Putative membrane protein	0.77	5.36E-06
llmg_0221	Similar to glycosyl transferase	0.77	5.47E-05
arcA	Arginine deiminase	0.77	1.25E-04
pepQ	Proline dipeptidase	0.78	6.88E-03
llmg_1920	Putative uncharacterized protein	0.78	9.11E-03
oppB2	Peptide transport system permease protein oppB2	0.78	3.98E-04
metA	Homoserine O-succinyltransferase	0.78	3.22E-05
llmg_1196	Putative uncharacterized protein	0.78	2.26E-04
ps606	Putative uncharacterized protein ps606	0.78	2.65E-05
radC	UPF0758 protein llmg_1515	0.79	6.06E-05
llmg_0168	Deoxyribonuclease	0.79	5.32E-05
cpdC	2',3'-cyclic-nucleotide 2'-phosphodiesterase	0.79	2.94E-05
llmg_0062	Putative membrane protein	0.79	5.02E-04
llmg_1390	Putative uncharacterized protein	0.79	7.65E-04
dinF	Damage-inducible protein DinF	0.79	3.53E-05
xerD	Integrase-recombinase	0.80	1.06E-02
ps511	Putative uncharacterized protein ps511	0.80	1.43E-03
mesJ	tRNA(Ile)-lysidine synthase	0.80	3.23E-05

llmg_0096	Putatve glyoxylase protein	0.81	7.17E-05
ps513	Putative uncharacterized protein ps513	0.81	1.39E-04
nifS	Putative iron-sulfur cofactor synthesis protein	0.82	5.38E-05
pbp2B	Penicillin-binding protein 2B	0.82	8.20E-04
pbpX	Penicillin-binding protein	0.82	2.85E-04
llmg_1834	Putative uncharacterized protein	0.82	2.34E-05
llmg_1016	Cationic transporter	0.82	1.71E-05
llmg_1180	Putative uncharacterized protein	0.83	1.15E-04
secY	Protein translocase subunit SecY	0.83	3.66E-05
llmg_0495	Putative uncharacterized protein	0.83	1.58E-05
ps122	Putative uncharacterized protein ps122	0.83	9.94E-04
llmg_1406	Putative uncharacterized protein	0.84	1.05E-04
pyrG	CTP synthase	0.84	2.23E-03
llmg_2541	Cation transporting ATPase	0.84	2.26E-05
llmg_1723	Putative secreted protein	0.84	5.59E-06
llmg_2323	Proteinase	0.85	5.33E-06
llmg_1475	UPF0291 protein llmg_1475	0.85	1.04E-05
dnaX	DNA polymerase III subunits gamma / tau	0.85	1.74E-06
llmg_0119	Putative Acyltransferase	0.85	1.04E-03
rbsC	Ribose transport system permease protein RbsC	0.85	1.91E-02
llmg_2209	tRNA-dihydrouridine synthase	0.85	1.60E-03
llmg_1612	Putative transposase helper protein for IS712A	0.86	2.26E-03
llmg_0476	Putative membrane protein	0.86	1.26E-06
llmg_2458	Putative fibronectin-binding protein	0.86	2.71E-06
llmg_0008	Putative HTH-type transcriptional regulator	0.86	2.73E-06
llmg_0295	Putative membrane protein	0.86	8.46E-03
ps610	Putative uncharacterized protein ps610	0.87	1.02E-05
llmg_1760	Queuosine precursor transporter QueT	0.87	1.34E-06
thiE	Thiamine-phosphate synthase	0.87	5.78E-05
llmg_0018	Similar to Beta-lactamase A	0.88	3.44E-06
llmg_0605	Oxidoreductase	0.88	6.94E-07
llmg_1540	Glycerol-3-phosphate acyltransferase	0.88	5.34E-04
llmg_1944	Putative uncharacterized protein	0.89	9.19E-07
ps124	Putative uncharacterized protein ps124	0.89	1.14E-04
lysQ	Amino-acid permease lysQ	0.89	3.15E-05
llmg_2457	Putative uncharacterized protein	0.89	3.18E-04
llmg_2562	Putative uncharacterized protein	0.90	4.33E-07
ftsX	Cell division protein ftsX homolog	0.90	1.65E-03
llmg_1411	Putative uncharacterized protein	0.91	1.09E-04
ps608	Putative uncharacterized protein ps608	0.91	7.93E-06
llmg_2516	Putative uncharacterized protein	0.91	4.86E-04
llmg_1101	Putative secreted protein	0.91	4.56E-04
llmg_0227	Putative membrane protein	0.92	4.92E-06
phnD	Phosphonate ABC transporter, phosphonate-binding protein phnD	0.93	6.30E-06
llmg_0294	Putative uncharacterized protein	0.94	1.47E-04
llmg_2369	Hypothetical secreted protein predicted by Glimmer/Critica	0.95	1.18E-05

dacB	DacB protein	0.96	2.81E-06
coaE	Dephospho-CoA kinase	0.96	4.91E-05
llmg_0856	Putative uncharacterized protein	0.97	2.20E-04
ps514	Hypothetical phage protein predicted by Glimmer/Critica	0.97	8.28E-06
llmg_2275	Putative uncharacterized protein	0.97	7.37E-05
ps609	Putative uncharacterized protein ps609	0.97	2.39E-03
ps517	Putative Dna Primase	0.97	4.97E-06
llmg_1074	Putative transposase helper protein for IS712A	0.98	1.52E-03
llmg_0736	Putative membrane protein	0.98	4.68E-05
ps518	Hypothetical phage protein predicted by Glimmer/Critica	0.98	1.31E-05
ps125	Phage dna replication	0.99	6.41E-04
llmg_0604	Ribonuclease Z	0.99	4.33E-05
mraY	Phospho-N-acetylmuramoyl-pentapeptide-transferase	0.99	4.12E-06
xerD	Putative integrase/recombinase	0.99	1.58E-05
mvaA	MvaA protein	0.99	2.00E-07
llmg_1748	Putative membrane protein	1.00	2.73E-05
rpoC	DNA-directed RNA polymerase subunit beta'	1.00	3.87E-03
pacL	Cation-transporting ATPase, E1-E2 family	1.01	3.92E-04
cydD	Cytochrome D ABC transporter ATP binding and permease protein	1.01	9.66E-07
llmg_0734	Putative amidase	1.01	5.20E-06
rgpF	Alpha-L-Rha alpha-1,2-L-rhamnosyltransferase RgpF	1.01	2.38E-05
llmg_1408	Putative uncharacterized protein	1.02	2.56E-07
rodA	Rod shape-determining protein RodA	1.02	2.23E-05
llmg_1749	Putative membrane protein	1.03	2.93E-06
llmg_0223	Putative galactofuranose transferase	1.03	2.60E-07
llmg_2514	Putative uncharacterized protein	1.03	1.18E-05
llmg_0375	Amino acid permease	1.03	5.36E-05
llmg_2222	Putative membrane protein	1.03	3.22E-05
rpmGA	50S ribosomal protein L33 2	1.04	2.04E-05
llmg_0705	Putative uncharacterized protein	1.04	2.01E-06
lacX	Galactose mutarotase related enzyme	1.04	5.69E-08
llmg_0439	Transcriptional regulator, LacI family	1.04	1.68E-05
llmg_2402	Putative uncharacterized protein	1.05	2.64E-06
ps516	Hypothetical phage protein predicted by Glimmer/Critica	1.06	3.51E-04
cydC	Cytochrome D ABC transporter ATP binding and permease protein	1.06	2.66E-06
llmg_1409	Putative uncharacterized protein	1.06	1.70E-06
llmg_2109	Putative transposase helper protein for IS712A	1.06	2.20E-04
llmg_0268	ABC transporter ATP binding protein	1.07	3.48E-06
llmg_2410	Putative uncharacterized protein	1.08	1.32E-05
pmrA	Multidrug resistance efflux pump	1.09	2.26E-05
ps515	Hypothetical phage protein predicted by Glimmer/Critica	1.10	1.14E-04
llmg_1577	Putative membrane protein	1.12	4.33E-07
ahpF	Alkyl hydroperoxide reductase subunit F	1.12	2.20E-06
llmg_2404	Putative uncharacterized protein	1.13	3.77E-06
kinC	Sensor histidine kinase	1.14	8.66E-06
llmg_0989	ABC transporter ATP binding and permease protein	1.14	8.81E-06

llmg_0870	Transporter	1.14	1.64E-05
ileS	Isoleucine--tRNA ligase	1.14	1.28E-05
llmg_0823	Putative transposase helper protein for IS712A	1.14	1.24E-04
llmg_2515	Putative uncharacterized protein	1.15	2.85E-07
llmg_1410	Putative uncharacterized protein	1.15	9.57E-05
llmg_2223	Putative uncharacterized protein	1.15	5.74E-07
mutY	A/G-specific adenine glycosylase	1.15	3.66E-07
llmg_2210	None	1.15	2.71E-06
llmg_0050	Putative transposase helper protein for IS712A	1.17	8.87E-04
llmg_1261	Putative transposase helper protein for IS712A	1.18	2.28E-05
llmg_2221	Putative membrane protein	1.18	8.92E-08
llmg_1066	Putative membrane protein	1.19	3.17E-06
llmg_0228	Putative membrane protein	1.19	4.72E-07
ps611	Putative uncharacterized protein ps611	1.20	1.79E-02
ypbC	Putative membrane protein	1.21	5.34E-05
llmg_2011	Putative amino acid permease	1.21	3.80E-05
llmg_0706	Putative uncharacterized protein	1.22	1.02E-05
rfbX	Putative O-antigen transporter	1.23	1.48E-06
llmg_2220	Putative membrane protein	1.23	2.92E-07
llmg_0704	Putative uncharacterized protein	1.25	9.66E-07
llmg_1229	Putative uncharacterized protein	1.25	2.99E-05
llmg_2513	Putative transport protein	1.26	9.66E-07
llmg_2049	Putative uncharacterized protein	1.26	3.54E-08
llmg_1084	Putative membrane protein	1.27	2.78E-07
pbuX	Xanthine/uracil permease	1.27	2.26E-05
llmg_2368	Putative uncharacterized protein	1.27	9.46E-07
llmg_1173	Putative uncharacterized protein	1.28	1.24E-08
glcU	Putative glucose uptake protein glcU	1.29	2.16E-07
llmg_1448	Putative membrane protein	1.30	2.10E-07
llmg_0269	ABC transporter ATP-binding and permease protein	1.31	5.26E-05
arcB	Ornithine carbamoyltransferase, catabolic	1.31	3.91E-06
llmg_1428	Putative uncharacterized protein	1.31	4.77E-06
llmg_1230	Putative uncharacterized protein	1.31	7.04E-08
cbiO	Putative cobalt ABC transporter ATP-binding protein	1.31	5.69E-08
llmg_2403	Putative uncharacterized protein	1.32	1.48E-07
acpD	FMN-dependent NADH-azoreductase	1.33	4.33E-07
comGB	Putative competence protein ComGB	1.34	1.64E-07
ubiA	Putative prenyltransferase, UbiA family	1.34	8.35E-07
menA	Prenyltransferase, UbiA family	1.35	1.66E-07
llmg_0226	Possible surface protein	1.35	2.07E-06
llmg_2219	Putative membrane protein	1.36	3.54E-07
llmg_1427	N-acetylmuramic acid 6-phosphate etherase	1.38	2.64E-06
llmg_1474	Putative voltage gated chloride channel	1.42	9.88E-06
llmg_2172	Putative nitroreductase	1.43	2.12E-05
comGC	Putative competence protein ComGC	1.44	2.57E-08
spoU	Putative tRNA (cytidine(34)-2'-O)-methyltransferase	1.46	1.19E-08

valS	Valine--tRNA ligase	1.46	4.95E-09
llmg_2290	Putative uncharacterized protein	1.46	2.10E-07
llmg_1447	Putative membrane protein	1.47	7.05E-08
llmg_1426	Sucrose-specific PTS system IIBC component	1.48	3.00E-06
llmg_1141	Putative transcriptional regulator	1.50	8.64E-06
llmg_0241	UPF0348 protein llmg_0241	1.54	2.28E-08
comGD	Putative competence protein ComGD	1.55	2.78E-07
arcC1	Carbamate kinase	1.57	1.67E-08
comGA	Putative competence protein ComGA	1.59	5.50E-09
llmg_2322	Putative uncharacterized protein	1.62	2.24E-07
cbiQ	Putative cobalt ABC transporter permease protein	1.63	3.31E-09
arcD1	Arginine/ornithine antiporter	1.65	4.72E-07
llmg_0240	Putative uncharacterized protein	1.68	1.81E-09
tnp1675	Putative transposase	1.83	1.78E-07
arcC2	Carbamate kinase	1.84	5.83E-08
nha	Na+/H+ antiporter	1.95	3.88E-06
llmg_1993	Hypothetical transporter	2.09	1.78E-07
llmg_1195	Riboflavin transporter RibU	2.37	7.28E-10

Table S4. The bacterial strains and plasmids used in this study

Strains or plasmids	Characteristics	Reference or source
Strains		
L. lactis MG1363	Prophage-cured and plasmid-free derivative of *L. lactis* subsp. *cremoris* NCDO 712	(18)
TM29	The endpoint high-temperature adapted mutant isolated from MG1363 (860 generations)	This study
JC022	MG1363 598,835 C>T (*pabC*)	This study
JC023	MG1363 905,914 G>A (*trmD*)	This study
JC024	MG1363 2,434,453 G>T (*llmg_2477*)	This study
JC026	MG1363 Δ*llmg_0962*	This study
JC027	MG1363 403,714 C>T (preceding *groESL*)	This study
JC028	MG1363 1,164,619 C>G (preceding *ribU*)	This study
JC029	MG1363 Δ(*llmg_1349-llmg1358*)	This study
JC030	MG1363 1,966,122 C>T 1,966,323C>T (*rpoC*)	This study
JC031	MG1363 2,497,054 G>A (*llmg_2541*)	This study
JC032	MG1363 230,889 G>A (*llmg_0242*)	This study
JC033	MG1363 208,637 A>G (*llmg_0219*)	This study
JC038	MG1363 2,358,022 G>A (*cdsA*)	This study
JC078	JC030 403,714 C>T (preceding *groESL*)	This study
JC081	JC078 2,358,022 G>A (*cdsA*)	
JC043	MG1363 with *groESL* promoter region from MG1363 fused to *gusA* and integrated into the TP901-1 attB site; Erm^r	This study
JC044	MG1363 with *groESL* promoter region from TM29 fused to *gusA* and integrated into the TP901-1 attB site; Erm^r	This study
JC045	MG1363 with *ribU* promoter region from MG1363 fused to *gusA* and integrated into the TP901-1 attB site; Erm^r	This study
JC046	MG1363 with *ribU* promoter region from TM29 fused to *gusA* and integrated into the TP901-1 attB site; Erm^r	This study
Plasmids		
pCS1966	Nonreplicating integration vector in *L. lactis*; Erm^r	(68)
pLB85	Promoter-reporter integration vector for *L. lactis*; Erm^r	(74)
pLB65	pCI372 derivative expressing bacteriophage TP901-1 integrase; Cat^r	(74)
pLP712	The protease/lactose plasmid isolated from *L. lactis* NCDO 712	(18)

Table S5. Oligos for RT-qPCR used in this study

Oligo name	Sequence
probe_pheS	5'-/56-FAM/TTG CGT ATG /ZEN/ATT GCT CCT GGT CGT /3IABkFQ/-3'
fwd_pheS	5'-GAC TGT GTG TCG CAT CAT CT-3'
rev_pheS	5'-CAA TGC AAG CTC GGA CAA TG-3'
probe_fabT	5'-/56-FAM/ACT TGA GTA /ZEN/TGG AAG GGC GAG CAA /3IABkFQ/-3'
fwd_fabT	5'-TTC AGC AGC AAG GTC AGA TT-3'
rev_fabT	5'-CCT CTG TTT CAT ACG AGG GTT C-3'
probe_accC	5'-/56-FAM/TTT GCT GGA /ZEN/CGT CGT GTC ATC GAA /3IABkFQ/-3'
fwd_accC	5'-GTA AAG TTT GAC GGA CGG TTT G-3'
rev_accC	5'-TCG CCA GTC TGG GAG ATA TTA-3'
probe_fabZ1	5'-/56-FAM/TGG CGG AAT /ZEN/TTC GCC TTA TCA ATT GC/3IABkFQ/-3'
fwd_fabZ1	5'-GAC ATC ACC AGG AGT CAC TTT-3'
rev_fabZ1	5'-TCG CTC AAG CAG GTT CAA T-3'
probe_fabI	5'-/56-FAM/AGC GAA CAG /ZEN/TAG ATT CAA GGG CAG C/3IABkFQ/-3'
fwd_fabI	5'-ATT GCT CCT GCA GAG ATT CC-3'
rev_fabI	5'-TTC GCG CCA TTC CTA ACT AC-3'
probe_alaS	5'-/56-FAM/TCG TTT ACT /ZEN/TCG TCG CGC GGT TAT /3IABkFQ/-3'
fwd_alaS	5'-TGG CAC GAG TGA AGC TAA A-3'
rev_alaS	5'-GAA ATG AAG GGC GTG GTT ATG-3'
probe_fabD	5'-/56-FAM/ATG GCT TCT /ZEN/GGG ATT GGT GGA CTT /3IABkFQ/-3'
fwd_fabD	5'-CCC TTT ACG AGG GCC TTT AG-3'
rev_fabD	5'-TGA CCG TTT GGG CTG TAT C-3'
probe_nha	5'-/56-FAM/TTG GTG ATC /ZEN/TCT GCC ATT GGT GGG /3IABkFQ/-3'
fwd_nha	5'-CGG GCA TCG ACA TCT TCT AAA-3
rev_nha	5'-GTC GCA CTG GTT ACA GGT TAT-3'
probe_arcD1	5'-/56-FAM/AGG TGT GTC /ZEN/AGA TTA TGC ACG TGC T/3IABkFQ/-3'
fwd_arcD1	5'-GCC CAA GCA GAT AAC CAA TAA C-3'
rev_arcD1	5'-TTG GTG GTA AAC AAA CCT GAA C-3
probe_cbiOQ	5'-/56-FAM/TGA AAT GGT /ZEN/CAG CAA GGT CGC CTA /3IABkFQ/-3'
fwd_cbiOQ	5'-CAG GTC TTG AGG ACG ATG ATT C-3'
rev_cbiOQ	5'-CCT AAG CGT AGC TGA GGA TAT TG-3'
probe_llmg_1993	5'-/56-FAM/ATG GGC TAC /ZEN/GGT GCT ATT CCT TGG /3IABkFQ/-3'
fwd_llmg_1993	5'-CTG ATC CCA TTT CCG TTA CCA-3'
rev_llmg_1993	5'-GGA ATA TCC CAG TTG CCT TCT AT-3'
probe_glcU	5'-/56-FAM/TCA GGA AGC /ZEN/CAA TTA GTC ATT GGT GGT /3IABkFQ/-3'
fwd_glcU	5'-ACT AAG ACA GCC ATC GCA ATA A-3'
rev_glcU	5'-GGT GTT TCT GTA GCA AGT CCA-3'
probe_pacL	5'-/56-FAM/CGT GCG TAC /ZEN/CAC CCT CAC ATT CTC /3IABkFQ/-3'
fwd_pacL	5'-CCT GCT ACT GCT AAA GGG AAG-3
rev_pacL	5'-GAA ATT GCT CTC ACA GTT CTT GG-3'

Table S6. Primers used in this study

Name	Sequence
Allelic replacement or knockout	
pabC_fwd	CGCGGATCCCCATTATTAAAAATGGAATG
pabC_rev	ACCGCTCGAGTTGCCAGATAAATAATCTTG
trmD_fwd	CGCGGATCCTTATGATTTTATATTTGCTG
trmD_rev	ACCGCTCGAGTTCCCATTCATAATTACGAG
llmg_2477_fwd	CGCGGATCCTTTTCTAAACTAAACAGGAG
llmg_2477_rev	CGCGGATCCTTTTCTAAACTAAACAGGAG
llmg_0962_up_fwd	CTAGTCTAGAGTTTAAAAGTCCAATAGATAAGG
llmg_0962_up_rev	CGCGGATCCAGTTTTCTCCGTTTTTGTG
llmg_0962_down_fwd	CGCGGATCCAATTAAAAAAAGACCTAGAA
llmg_0962_down_rev	GGTACTCGAGCTTAGTGAATCTACAACCA
groESL_fwd	ACGCGGATCCCAACGATAGTGTGAAAATTC
groESL_rev	ACCGCTCGAGGCGATTGCTGATTTATCGTG
ribU_fwd	ACGCGGATCCGATGATAAAGGACGTCAAAC
ribU_rev	ACCGCTCGAGAATAAAAATGTTTCTTTAGG
llmg_1349-1358_up_fwd	CTAGTCTAGAGATAGAAATCATCGAACTTA
llmg_1349-1358_up_rev	AAAACTGCAGAAATTTACTGTTATAGTAATACTA
llmg_1349-1358_down_fwd	AAAACTGCAGTCGTTACTTTCCACCAGACT
llmg_1349-1358_down_rev	ACCGCTCGAGATTTATATTGACATTAAGTCAAGT
rpoC_fwd	GGTACTCGAGAATGACAGAGATTTTATGTT
rpoC_rev	CTAGTCTAGAAGTTGGTACGTAGTCCAGGT
llmg_2541_fwd	ACGCGGATCCCCAGAGTAAGTCCTGAAGAC
llmg_2541_rev	ACCGCTCGAGTATTGGTTCTTGATTTGACA
llmg_0242_fwd	ACGCGGATCCAGCTTATTTTCCACTTTTGC
llmg_0242_rev	ACCGCTCGAGAATCAGGCATTCTAAGCATC
llmg_0219_fwd	ACGCGGATCCAGAACTTGGAACAGTGTCCG
llmg_0219_rev	ACCGCTCGAGATAAGAAACAGCTGGGCAAC
cdsA_fwd	TTTCCTTTATGAGTATGGTT
cdsA_rev	AGCTTGATTTGACAGAGTTT
GusA activity	
groESL_P_fwd	CTAGTCTAGACTCCCTTTCTGATTTATTTG
groESL_P_rev	AAAACTGCAGAACTACGCGATTCTCTAAAG
ribU_P_fwd	CTAGTCTAGATGCGCAAGGAAAATAGTTTA
ribU_P_rev	AAAACTGCAGAATTAATACCATCCGACGTG
